# How Older Indigenous Women Living in High-Income Countries Use Digital Health Technology: Systematic Review

**DOI:** 10.2196/41984

**Published:** 2023-04-18

**Authors:** Connie Henson, Felicity Chapman, Gina Shepherd, Bronwyn Carlson, Boe Rambaldini, Kylie Gwynne

**Affiliations:** 1 Department of Health Sciences Faculty of Medicine, Health and Human Sciences Macquarie University Sydney Australia; 2 Djurali Aboriginal and Torres Strait Islander Research and Education Macquarie University Sydney Australia; 3 Centre for Global Indigenous Futures Macquarie University Sydney Australia; 4 Department of Indigenous Studies Faculty of Arts Macquarie University Sydney Australia

**Keywords:** digital health, Aboriginal and Torres Strait Islander, Indigenous, Aboriginal, health technology, engagement, co-design, cultural safety, older Ingenuous women, older women, older adult, gerontology, lived experience, patient experience, ethic, minority, minorities, elder, usability, acceptability, preference, PRISMA, systematic review, review methodology, social media, wearable

## Abstract

**Background:**

Research associated with digital health technologies similar to the technologies themselves has proliferated in the last 2 decades. There are calls for these technologies to provide cost-effective health care for underserved populations. However, the research community has also underserved many of these populations. Older Indigenous women are one such segment of the population.

**Objective:**

Our objective is to systematically review the literature to consolidate and document what we know about how older Indigenous women living in high-income countries use digital health technology to enhance their health.

**Methods:**

We analyzed the peer-reviewed literature by systematically searching 8 databases in March 2022. We included studies published between January 2006 and March 2022 with original data specific to older Indigenous women from high-income countries that reported on the effectiveness, acceptability, and usability of some user-focused digital health technology. We incorporated 2 measures of quality for each study. We also conducted a thematic analysis and a lived experience analysis, which examined each paper from the perspectives of older Indigenous women. We followed the PRISMA (Preferred Reporting Items for Systematic Reviews and Meta-Analyses) guidelines in this study.

**Results:**

Three papers met the inclusion criteria. The key findings were that older Indigenous women do not see themselves reflected in mainstream health messaging or other digital health offerings. They prefer an approach that considers their uniqueness and diversity. We also identified 2 significant gaps in the literature. First, research reporting on older Indigenous women from high-income countries’ experiences with digital health technology is minimal. Second, the limited research related to older Indigenous women has not consistently engaged Indigenous people in the research process or governance.

**Conclusions:**

Older Indigenous women want digital health technologies to respond to their needs and preferences. Research is needed to understand their requirements and preferences to ensure equity as we move toward greater adoption of digital health technology. Engaging older Indigenous women throughout the research is essential to ensuring that digital health products and services are safe, usable, effective, and acceptable for older Indigenous women.

## Introduction

Digital technology is changing how we care for our health. A proliferation of new platforms, smartphone apps, and devices promises new ways to detect, monitor, and communicate health indicators and impact behaviors. The uptake of these new technologies for health purposes has grown steadily since the early days of static websites [1,2]. It strengthened with the advent of social media and during the ongoing COVID-19 pandemic [3,4]. The World Health Organization (WHO) has asserted that digital health technology has the potential to provide cost-effective services for underserved populations [5]. However, evidence-based assessment of efficacy and safety is essential, particularly for underserved populations.

Many academic and industry research papers examine how and why people use digital health technologies for the overall population in most high-income countries. Research for smaller segments of the population is sparser, particularly for those categorized as vulnerable and underserved populations. Although Indigenous health care providers were early adopters of digital health technologies [6], the peer-reviewed research on the efficacy and cultural safety initially lagged behind implementation programs. For example, across this continent now colonially known as Australia, a 2014 scoping review found that while there were numerous social media and app-based health promotion programs for Aboriginal and Torres Strait Islander people, there was limited peer-reviewed literature on social media and no peer-reviewed research on the efficacy of mobile apps for this population [7]. However, research on digital health for Indigenous populations has increased in the last few years. Both original research and systematic reviews published in the previous few years examine a range of digital health technologies used by Indigenous people. This growing body of research demonstrates that Indigenous people use digital health tools to seek help to address various health issues, including nutrition, smoking, antenatal education, cancer, heart health, diabetes, and mental health [7-20]. Moreover, Carlson et al [21] suggested that social media may be a particularly effective medium for health promotion for Indigenous people, as it is in keeping with Indigenous ways of being, including principles of reciprocity, self-determination, and relationism. Likewise, Maxwell et al [22] found that wearable digital health devices can be a helpful tool for Indigenous women, for example, contributing to achieving personal health and fitness goals and increasing accountability and empowerment. As this literature base matures, it is important to focus research efforts on different age and gender cohorts within the broader population of Indigenous people, who are likely to have different needs and requirements than the overall population of Indigenous people. Older Indigenous women are one such group. Our team acknowledges the dynamic nature of language and respect the importance of language and meaning. We have developed a statement about our use of language related to gender and age and this is provided at the end of the paper.

Older Indigenous women in high-income countries are vulnerable to discrimination and increased risk of chronic diseases and cancer because of their age and the health-damaging impacts of colonization and racism experienced by Indigenous people [23-26]. Older Indigenous women have different life experiences, responsibilities, opportunities, and strengths compared to other people. Our definition of “older” is in keeping with a person’s age when internet usage became common. We, therefore, defined “older” as the generation known as seniors, baby boomers, and Generation X, who did not grow up with the internet [27]. Millennials born after 1980 (<41 years old) are generally considered more comfortable with technology, especially social or interactive media, than older generations [28]. Older women’s diverse experiences will likely impact their perspectives, capabilities, interest, and comfort in using digital health technology. Therefore, it is essential to create an evidence base for how, why, and for what purposes older Indigenous women use digital health technology. Creating a body of evidence is a time-sensitive critical concern as we incorporate more digital health services and programs into our health systems and practices. Understanding what technologies are effective and acceptable, as well as the barriers and enablers for older Indigenous women in high-income countries, is a critical first step in designing an equitable digital health research and health care strategy for all citizens. This evidence base is also vital for health care providers and older Indigenous women to make informed decisions about using digital health technologies to enhance or communicate about their health.

This study aimed to consolidate and document what we know about how older Indigenous women living in high-income countries use digital health technology to enhance their health or health education for themselves or their communities. The focus of this study is high-income countries because Indigenous people from different high-income countries (eg, the United States and Canada) are diverse but have some similarities in terms of the availability of personal resources. Likewise, they have similarities in terms of disparities in health and life expectancy between themselves and the wider populations of their countries. Moreover, public resources, including health resources, differ significantly between high- and low-income countries. We identified 6 specific questions for this review (Textbox 1).

**Review questions.**

**Review questions**

1. What digital technology do older Indigenous women in high-income countries use to promote health?
2. Which aspects of older Indigenous women’s health, well-being, or disease have these digital technologies addressed?
3. What is the evidence for the effectiveness of digital technology–based health programs for older Indigenous women?
4. What factors (barriers and enablers) impact the use of digital technologies by older Indigenous women to promote health?
5. What issues related to cultural safety or sensitivity for older Indigenous women’s use of digital health programs have been evaluated or observed?
6. What does the evidence from this review tell us about designing future digital technology–based health programs with older Aboriginal and Torres Strait Islander women.

## Methods

### Design

We conducted this systematic review of peer-reviewed literature according to the PRISMA (Preferred Reporting Items for Systematic Reviews and Meta-Analyses) guidelines [29-31]. This study is registered with the PROSPERO international prospective register of systematic reviews (CRD42022309623).

### Aboriginal Governance

Health research methodology has historically minimized or excluded Indigenous ways of thinking, learning, and doing science. To empower and amplify Indigenous perspectives, we incorporated Aboriginal and Torres Strait Islander governance into this research by establishing an Aboriginal Project Governance (APG) group to oversee and participate in this study. The APG comprises 3 citizen scientists who reflected the research population of older Indigenous women. The review questions, search terms, and inclusion criteria were codeveloped and implemented in collaboration with the APG. Two members worked alongside the academic researchers throughout all components of this research, including thematic data analysis and providing perspective from their lived experiences, and are listed as contributing authors for this paper.

### Ethics Approval

The study adhered to the guidelines for ethical research with Aboriginal and Torres Strait Islander populations. We obtained approvals from the Aboriginal Health and Medical Research Council Ethics Committee (reference 1862/21) on December 1, 2021. Our APG and the Aboriginal Health and Medical Research Council Ethics Committee approved this manuscript.

### Search Strategy

We conducted the search between March 8 and 14, 2022. It included 8 databases. We searched the following electronic bibliographic databases: MEDLINE, Embase, Scopus, CINAHL, PubMed, PsycINFO, and Web of Science Aboriginal and Torres Strait Islander Health Informit. We limited the search to peer-reviewed studies published in English since January 2006, when Facebook was made available to the public.

The searches were conducted using the following search terms: Aboriginal OR Indigenous OR “First Nations” OR “Torres Strait Islander” OR “Native American” OR Maori OR Sami OR Inuit OR Ainu OR “American Indian.”

AND

“Mobile application” OR Smartphone OR Internet OR “Social Media” OR Facebook OR Snapchat OR Instagram OR Twitter OR TicToc OR Pinterest OR youtube OR LinkedIn OR blogging OR email OR “social networking” OR “internet use” OR “internet application”

AND

“Consumer health information” OR “health literacy” OR “health promotion” OR “Health Education” OR “User Health” OR Disease OR “Chronic Disease” OR Illness OR Cancer OR neoplasms OR “cardiovascular disease” OR Diabetes OR “Healthy people programs” OR “Weight reduction” OR “Sex education” OR “Smoking prevention” OR “tobacco use” OR Smoking OR Vaping OR Selfcare OR “Quality of Life” OR Stress OR Wellbeing OR Coping OR Alcoholism.

We adapted the terms for each of the 8 databases (Multimedia Appendix 1). 

### Eligibility Criteria

The inclusion criteria for studies included in this review were (1) original data, (2) data specific to older Indigenous women from high-income countries, and (3) the study reported on the effectiveness, acceptability, or usability of some user-focused digital health technology. We excluded papers not focused on Indigenous women living in high-income countries. Likewise, studies that included older Indigenous women in the sample but did not report results specifically about this cohort were excluded. This exclusion criterion is necessary because this cohort (older Indigenous women living in high-income countries) is an important subpopulation within a larger population of Indigenous people, with unique needs and desires, potentially needing or wanting unique digital interventions and tools. Reporting on what is relevant for the overall Indigenous population runs the risk of obscuring the needs and requirements of different age and gender cohorts. Concomitantly, papers were excluded if the study did not evaluate some user-based health technology’s effectiveness, acceptability, or usability.

### Screening

We removed duplicates using EndNote (version 20; Clarivate) and Rayyan.ai [32-34]. All remaining titles were screened, and abstracts or full texts were reviewed when the abstract did not include sufficient information to assess against the inclusion and exclusion criteria. Discrepancies were resolved by consensus by the first and last authors.

### Extraction Strategy

An extraction template was designed by 1 author, CH, and reviewed by a second author, KG. Extracted data included title, year, authors, publication date, study design or type, the aim of the study, sample size, community type (urban, rural, and remote), technology type, program features and components, and information related to the engagement of Indigenous people in the research design and implementation, outcome measures, results, and main conclusions. The data was extracted by CH and checked by KG. We resolved disagreements through discussion.

### Analysis

We incorporated 2 measures of quality for each study. We also conducted a thematic analysis (TA) [35] and a lived experience analysis (LEA), which examined each paper from the perspectives of older Indigenous women.

#### Quality Assessment

We assessed the quality of each study via 2 methods. We evaluated the qualitative studies with the Critical Appraisal Skills Program (CASP) tool [36]. We assessed the cross-sectional study with the Appraisal Tool for Cross-sectional Studies (AXIS) [37]. The CASP and the AXIS quality evaluation tools focus on the reliability and replicability of the methods and reporting of the results. These and other typical measures of research quality usually do not consider the cultural safety and intensity of cultural engagement. Given the ongoing impacts of colonization and systemic racism, as well as a history of health research protocols that objectified, deceived, and discredited Indigenous people, a high level of cultural engagement is an essential component of research quality involving Indigenous people. Moreover, cultural engagement and Indigenous leadership contribute to empowerment, greater trust, and the sustainability of health programs for Indigenous people [38-43]. Therefore, we also appraised the 3 included studies to determine the intensity of cultural engagement using the Cultural Engagement Intensity (CEI) [44]. The CEI tool is based on the criteria set out by the Australian National Health and Medical Research Council’s ethical guidelines for any research involving Aboriginal and Torres Strait Islanders [45]. The five criteria are (1) the issue identified by the community, (2) Indigenous governance, (3) capacity building, (4) cultural consideration in the design, and (5) respecting community experience. Studies scoring 4 or 5 are considered strong, those scoring 2-3 are moderate, and those scoring 0-2 are considered weak.

A comment from an APG member, who is also an artist, sums up our purpose and approach to measuring cultural intensity.

a weaver will take the time to choose the most appropriate fibers to ensure a resilient and enduring weaving. As part of the selection process, fiber that is seen as weak or inflexible, will be discarded as using these unsuitable fibers is contrary to the desired outcome. This is the same as a critical literature review. Many articles may be gathered, reviewed and assessed for the suitability to help strengthen and shape the weaving; writing that is focused on amplifying the deficit discourse will be acknowledged and set aside as it provides no value to progressing the outcome, except as a reminder to avoid the camouflaged rabbit traps intent on keeping the Western discourse intact

Felicity Chapman, April 6, 2022

Four authors, CH, FC, GS, and KG, rated each of the studies according to the criteria for the quality tools. The differences were resolved by discussion, privileging the perspectives of the Aboriginal researchers (GS and FC) when applying the CEI.

#### Thematic Analysis

In TA [46], if we identified an issue or factor in 2 or more studies, we considered it a theme. To determine the themes, 3 authors (CH, FC, and GS) independently analyzed and subsequently discussed the included papers. Finally, a fourth author (KG) reviewed the analysis, and we reached a consensus through discussion.

#### Lived Experience Analysis

Two of the APG members examined the papers’ findings with respect to the value the study provided for older Indigenous women. Likewise, they evaluated the presentation of results in terms of deficit- or strength-based framing. They also considered the extent to which the presentation of the findings showed respect for Indigenous peoples, values, and cultures. This analysis was facilitated by recording and transcribing discussions between the lead researcher, CH, and APG members (GS and FC), whose perspectives were privileged throughout the study.

APG members consented to participate, including the recording of meetings, and gave explicit permission for their specific quotes to be used throughout this paper.

## Results

### Overview

**Figure 1:**
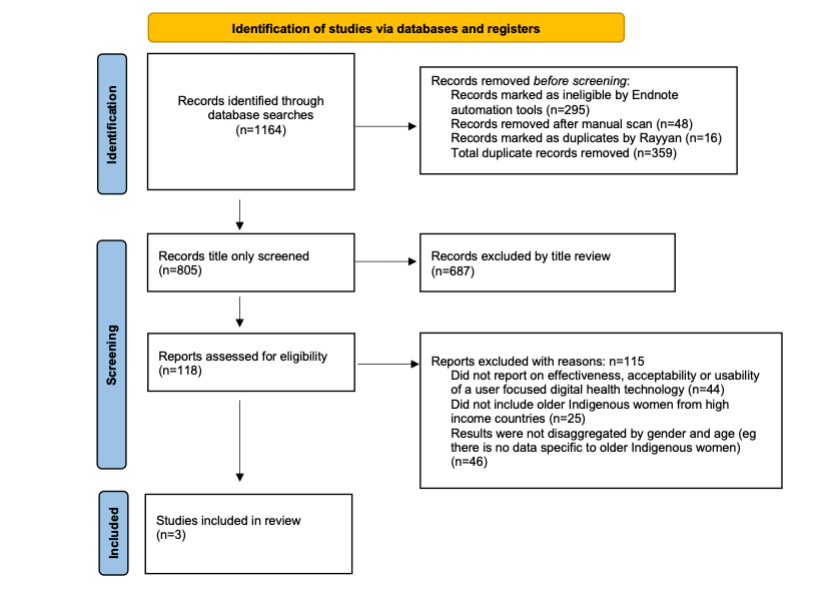
PRISMA (Preferred Reporting Items for Systematic Reviews and Meta-Analyses) flow diagram of study selection [30].

We retrieved 1164 articles and removed 359 duplicates, leaving 805 papers. We used Rayyan.ai [32] to screen these studies. We then excluded 687 articles based on reviewing the title, leaving 118. Next, we examined the abstract and the full text when insufficient detail was not included in the abstract, thus excluding an additional 115 articles with reasons for exclusion recorded (Figure 1).

This review includes 3 studies, of which 2 are with older Canadian Aboriginal women. The same team authored these 2 papers and used the same qualitative data set (interviews with 25 women). The third paper is with Australian Aboriginal women and is a descriptive quantitative survey study with 132 participants (Table 1).

**Table 1:** Summary of included papers.

Publication	Study summary	Findings or conclusions	Assessment of quality
Hoffman-Goetz and Friedman [47], 2007	A qualitative interview study included 25 older Canadian Aboriginal women in determining women’s beliefs about what constitutes credible and high-quality cancer prevention web-based resources	Involve community in developing web-based health resources to ensure that the information includes: cues for action, allopathic and traditional medical options or contacts, and references from credible sources, for example, Elders and respected community members.	CASP^a^=8 (good quality); CEI^b^=0 (weak engagement)
Friedman and Hoffman-Goetz [48], 2007	A qualitative interview study included 25 older Canadian Aboriginal women in determining women’s opinions of the usefulness and relevance of breast cancer information on the internet.	Collaborate with cancer agencies to improve cultural sensitivity, language, and tone of cancer information for Aboriginal women, and attend to spiritual and cultural beliefs about cancer and traditional medicines.	CASP=8 (good quality); CEI=0 (weak engagement)
Gould et al [49], 2020	A cross-sectional survey included 132 women to identify supports Aboriginal women use for their health; the health of a baby or child, or specifically for smoking cessation, and to determine predictors for health-seeking for self, baby or child or smoking	Age, smoking status, and having a child at home impacted different aspects of channels for health information seeking. This information could assist in targeting approaches for health promotion.	AXIS^c^=15 (good quality); CEI=2 (moderate engagement)

^a^CASP: Critical Appraisal Skills Program.

^b^CEI: Cultural Engagement Intensity.

^c^AXIS: Appraisal Tool for Cross-sectional Studies.

### Quality Appraisal

We rated the 3 papers in this review as of good quality based on assessment with either the CASP or the AXIS. Regarding cultural engagement, the 2 qualitative studies in this review did not report any aspect of Indigenous engagement in the design or governance of the research. The more recently published cross-sectional survey study only met 2 of the 5 criteria as assessed by the CEI: capacity building and cultural consideration in the design. Respecting community experience was implied but not described. There was no mention of the research communities’ involvement in identifying the research issue or aims, nor was there any description of Indigenous governance. The less intense cultural engagement in these studies suggests the need for caution in relying on these studies’ findings.

### Thematic Analysis

We identified 1 overarching theme and 3 subthemes. We titled the overarching theme “See me.” Older Indigenous women know they have different needs and preferences and do not see themselves reflected in mainstream health messaging or digital health offerings. Subthemes emerging from this analysis are all related to a preference for a personalized approach that considers their gender and Indigeneity.

The 3 subthemes we identified are cultural integration, relationships, and practicality.

1. Cultural integration signifies credibility: Women perceive health information from a culturally recognized source as more relevant and credible. Cultural integration includes spiritual and cultural beliefs and practices that address holistic health needs. Their preferences included culture integration, including the source, content, and style (eg, a culturally relevant person conveying culturally relevant information, specific to Indigenous women, in a narrative or storytelling style).
2. Relationships matter: Elders, family, and professionals are considered important sources of health information. In 1 study examining web-based and “in-real-life” supports, a conversation with a health provider was valued over social media or the internet for health-related information for older women. Family or community relationships came up in all 3 studies.
3. Practicality: Action-oriented, clear, and specific health information is preferred; generalized information is insufficient. Older Indigenous women prefer clear messages, including risks and specific alternatives. They also like simple language, with illustrations favored over words.

### Lived Experience Analysis

The APG members identified 3 themes related to research relevance and framing: acknowledging Indigenous people’s contributions, the necessity of moving to strength-based framing, and that research needs to be accessible.

#### Acknowledge Indigenous People’s Contribution

APG members observed that when Indigenous assistants were involved in the research but were not authors or not acknowledged by name, they wondered why they were left out. The APG members suggested that naming the Indigenous assistants or other contributors would add credibility to the study and show a more profound respect for Indigenous people’s cultural contribution to the research.

…they're specifically being really vague. In using a positions title, rather than actually mentioning who actually did the work

…it's easy enough to say, Oh, look, we have Aboriginal research assistants, but nowhere do they name them…

#### Move to Strength-Based Framing

The APG noted that it is time for researchers working with Indigenous people to move away from focusing on what is wrong or framing difference as less than. Instead, the members want to see health research that emphasizes strengths and resilience, suggesting that this framing will show respect and ensure the study feels relevant and are valued by Indigenous people.

…I still feel that they've very much focused on the deficit discourse through these summaries, which really doesn't lead to respecting your community experience and actually focusing on what they're actually doing really well…

#### Research Needs to Be Accessible

The APG members indicated that presenting health research results in a way that is accessible to nonacademics would engender trust. More accessible language would also ensure that practitioners and the people that the study is about can understand the results and determine the value of the findings.

…*When they use all these very highly technical terms, it’s so they can keep it at the upper echelon. Like they are the only for the ones who really need to know, or are actually meant to read this, because it's not really for normal people…*

…*So as soon as I start to see a lot of that very big wordy stuff, and I have to google search to find out what they mean, I tend to go, Yeah, I don't trust you. And it is instant - I don't trust what I'm reading…*

#### Answering Our Specific Research Questions

Addressing some of the specific research questions set out for this study (Table 2) is challenging given the dearth of research on older Indigenous women’s use of health technology and the limited cultural intensity described in the research processes for the included studies. Nevertheless, we have gleaned some helpful information from these studies.

**Table 2:** Specific questions.

Question	Evidence
1. What digital technology do older Indigenous women in high-income countries use to promote health?	Based on the limited data available older women appear to use internet websites and Facebook to access health information. They are more likely to seek help from health care providers than younger Indigenous women There is no data related to how they share health-related information
2. Which aspects of older Indigenous women’s health, well-being, or diseases have the digital technologies addressed?	Cancer, general health, smoking, and a child’s health are the only aspects of health addressed
3. What is the evidence for the effectiveness of digital technology–based health programs for older Indigenous women?	None of the studies measured the impact of a digital health program on women’s health
4. What factors (barriers and enablers) impact the use of digital technologies by older Indigenous women to promote health?	In addition to avoiding cultural insensitivity, older women appreciated illustrations Older women assessed complex, overly wordy presentations as a barrier Two of the 3 studies directly compared women’s perspectives on mainstream health information and a tailored presentation of information related to breast cancer. The themes arising from these studies indicate that mainstream health information is not adequate
5. What issues related to cultural safety and sensitivity for older Indigenous women’s use of digital health programs have been evaluated or observed?	Older Indigenous women find health information tailored to their culture more accessible
6. What does the evidence from this review tell us about designing future digital technology–based health programs with older Aboriginal and Torres Strait Islander women?	A key finding is that we don’t know much about what older Indigenous women consider essential in digital health technologies, what health concerns they would like addressed, or what information and programs they need

## Discussion

### Principal Findings

Only 3 relevant studies published between 2006 and 2022 were identified in our co-designed PRISMA-based systematic review of the peer-reviewed literature, which investigated the evidence for how older Indigenous women use digital health technology. Still, our co-design methodology, including the LEA combined with the TA, illuminated several themes and uncovered gaps in the evidence-based literature.

There are 2 significant gaps in the literature. First, digital health research, specifically with older Indigenous women, is minimal. Second, research with this cohort does not consistently engage (or at least does not report on) governance or engagement with Indigenous people in research design or implementation.

A key finding is that older Indigenous women from high-income countries do not see themselves reflected in the digital health offerings. Older Indigenous women understand the diversity of different cohorts of Indigenous people. They want to be acknowledged and have their unique health and cultural needs addressed by digital health technologies. The overarching theme emerging from the TA, titled “See Me,” is also relevant to describe the lack of research on older Indigenous women’s preferences and use of digital health technologies.

While research in the last few years has contributed to our understanding of how and for what reasons Indigenous people in general access digital health technologies, we do not know much about the perspectives and experiences of older Indigenous women. This lack of attention may be an artifact of the relative maturity of the literature base and points to the importance of this review to highlight the pressing need for more research.

Research that recognizes the immense diversity of Indigenous people and specifically focuses on and engages older Indigenous women will contribute to health equity and potentially play a part in improving community health more broadly. From an equity standpoint, older Indigenous women have specific health needs, such as certain cancers and chronic health conditions, compared to other people and thus require particular research attention. It is also essential to remember that Elders and older Indigenous women are often family caregivers. Moreover, they are respected and trusted influencers within their communities [50-52]. So they are well-positioned to share health-related information more broadly and to become role models and mentors for using digital health technologies, multiplying the benefit to the community.

### Recommendations

Our chief recommendation is to urgently produce more culturally intense digital health research with older Indigenous women. Future digital health research should address the health interests and health conditions relevant to older Indigenous women and report results that segment this cohort. Data specific to older Indigenous women will provide a foundation for designing tailored digital health programs, addressing issues most relevant for older Indigenous women, which consider the intersection of their gender, age, and Indigeneity. Socioeconomics and access are also important factors to be included. Moreover, digital health research should be co-designed to build on strengths within this cohort, including Indigenous people’s propensity for use and innovation with technology and older Indigenous women’s position of respect and influence within their communities.

The LEA highlighted 3 specific recommendations for health research reporting. Following these recommendations will help ensure Indigenous peoples and cultures are respected and confirm that the research is perceived as relevant and credible. First, explicitly acknowledge Indigenous people’s contributions to the study. Second, present findings in a strength-based frame, and finally, report findings in a way that is accessible for potential users of the results. We recommend that all health research include some LEA to ensure the methods and findings are relevant and presented in a way that is respectful and engaging for users. We further recommend that future research explicitly report on Indigenous governance, engagement, and leadership from the project’s inception through implementation and dissemination of results. This inclusion is vital to a modern, robust research methodology and reflects current ethical guidelines and societal expectations for research involving Indigenous people. Moreover, inclusive research that deeply engages Indigenous people will contribute to safe research programs, produce desired knowledge, and be more likely to contribute insights that lead to sustainable positive health outcomes.

### Limitations and Strengths

The review may be affected by the tight inclusion criteria, thereby excluding studies that included older Indigenous women and potentially reducing the knowledge we can glean for this important population. It is possible that, despite differences, older Indigenous women do not experience digital health technology differently from other people, and thus, aggregated samples are sufficient. However, we believe this is a question that needs to be addressed.

Indigenous governance and privileging the perspectives of co-researchers with lived experience is a strength of this research and contributed to a more rich and more relevant analysis. In addition to providing valuable views on the content of the reviewed studies, this approach provided insights about research methods and reporting of findings not typically surfaced in systematic reviews. Moreover, the LEA resulted in specific recommendations for researchers that, if followed, can contribute to strengthened credibility and trust in science.

### Conclusions

People of all demographics are keen to use digital health technologies. However, they also want those technologies to respond to their needs and preferences. Given the unique experiences, responsibilities, strengths, health needs, and interests of older Indigenous women, understanding their perspectives is essential for tailoring digital health programs and messaging.

While commercial “realities” make tailoring for a smaller population less appealing, health is a fundamental right. Equity must be built into the system, including the research that underpins new digital health programs and systems. Building equity for this cohort requires engagement and prioritizing older Indigenous women’s perspectives. This engagement is essential to ensuring that digital health products and services are safe, usable, and effective. High-income countries have the means to be inclusive to ensure that all citizens can access what is necessary to achieve and maintain their health.

While historically, we have developed health tools, techniques, and services that suit the mainstream without consideration for the more vulnerable and less visible members of our society, the advent of digital health allows us to rethink and reboot our approach. There is a role for many stakeholders. For example, developers of apps, user-focused devices, and platforms can purposefully include a diverse group of users in the early stages of development to ensure safety and equity. In addition, researchers can intentionally sample and report on cohorts we know have different needs and requirements. Finally, there is a vital role for the government. Because policies and regulations governing health technology are only emerging in most high-income countries, now is the time to create policy and regulation to reshape our health systems and practices. Building inclusiveness at every step will ultimately create greater equity and a more robust health system.

### Statement Related to Gender and Age Language in the Manuscript

This research focused on how older Indigenous women use digital health. Our definition of “older” is in keeping with a person’s age when internet usage became common. We are therefore defining “older” as generations known as seniors, baby boomers, and Generation X, who did not grow up with the internet [27]. Millennials born after 1980 (<41 years old) are generally thought to be more comfortable with technology and especially social or interactive media compared with older generations [28].

Participants in our studies self-identify their gender. Therefore, an Indigenous person who identifies themselves as a woman some or all of the time is included in the studies focused on women. For our studies that include people other than women, we provide various gender options. People are encouraged to self-identify with any description or choose not to describe their gender.

## Appendix

Multimedia Appendix 1 Detailed search strategy for all databases.

## Abbreviations

APG: Aboriginal Project Governance
AXIS: Appraisal Tool for Cross-sectional Studies
CASP: Critical Appraisal Skills Program
CEI: Cultural Engagement Intensity
LEA: lived experience analysis
PRISMA: Preferred Reporting Items for Systematic Reviews and Meta-Analyses
TA: thematic analysis
WHO: World Health Organization
