# Supplementary material for: How Older Indigenous Women Living in High-Income Countries Use Digital Health Technology: Systematic Review
Source: J Med Internet Res. 2023 Apr 18;25:e41984. doi: 10.2196/41984 (PMC10155089; doi:10.2196/41984)
Supplement: Multimedia Appendix 1 [file jmir_v25i1e41984_app1.docx]

This document is the history of Systematic Review: How older Indigenous women use digital tech to improve health. The document includes information on:

- Databases searched an all results recorded in including notes on the process. All searches were copy/pasted from the databases
- Screening History Articles retrieved and screening process

**Table of Contents**

[PubMed 2022.03.14 2](#_Toc112325380)

[Notes about this database search 2](#_Toc112325381)

[Actual search terms for PubMed 2](#_Toc112325382)

[Link to search strategy for PubMed 4](#_Toc112325383)

[References for PubMed 4](#_Toc112325384)

[Web of Science 2022.03.09 10](#_Toc112325385)

[Notes about this database search 10](#_Toc112325386)

[Actual search terms for Web of Science 10](#_Toc112325387)

[Link to search strategy for Web of Science 11](#_Toc112325388)

[References found by Web of Science 11](#_Toc112325389)

[SCOPUS. 2022.03.09 17](#_Toc112325390)

[Notes about this database search 17](#_Toc112325391)

[Actual search terms for SCOPUS 17](#_Toc112325392)

[Link to saved search for SCOPUS 17](#_Toc112325393)

[References found by SCOPUS 17](#_Toc112325394)

[CINHAL 2022.03.08 24](#_Toc112325395)

[Notes about this database search 24](#_Toc112325396)

[Actual search terms for CINHAL 24](#_Toc112325397)

[Link to saved search for CINHAL 42](#_Toc112325398)

[References found by CINHAL 2022.03.08 42](#_Toc112325399)

[PsychoInfo-Ovid 2022.03.08 52](#_Toc112325400)

[Notes about this database search 52](#_Toc112325401)

[Actual search terms for PsychoInfo-Ovid 52](#_Toc112325402)

[Link to saved search for PsychoInfo-Ovid 53](#_Toc112325403)

[References found by PsychInfo 2022.03.08 53](#_Toc112325404)

[Embase – Ovid 2022.03.08 59](#_Toc112325405)

[Notes about this database search 59](#_Toc112325406)

[Actual search terms Embase – Ovid 2022.03.08 59](#_Toc112325407)

[Link to saved search 60](#_Toc112325408)

[References found by Embase-Ovid 2022.03.08 60](#_Toc112325409)

[Medline Ovid 2022.03.08 70](#_Toc112325410)

[Notes about this database search 70](#_Toc112325411)

[Actual search terms for Medline Ovid 70](#_Toc112325412)

[Link to saved search for Medline Ovid 71](#_Toc112325413)

[References found by Medline_Ovid 2022.03.08 71](#_Toc112325414)

[Informit 2022.03.14 78](#_Toc112325415)

[Notes about this database search 78](#_Toc112325416)

[Actual search terms for Informit 79](#_Toc112325417)

[Link to saved search for Informit 79](#_Toc112325418)

[References found by Informit 2022.03.07 80](#_Toc112325419)

[Screening History 83](#_Toc112325420)

[1164 total retrieved 83](#_Toc112325421)

[805 papers screened via title only 83](#_Toc112325422)

[118 Papers reviewed abstract or full text 83](#_Toc112325423)

# PubMed 2022.03.14

## Notes about this database search

Exactly what I did:

1. I created three searches by first adding the MeSH terms, and adding with OR the TW then searching the entire grouping as per below.
2. Then combined all three with AND
3. Than added filters for age and years

I tried using filters for the type of article but it ended up reducing the number of articles found too much. Same with the young adults, which I ultimately left in the search

MeSH: American Native Continental Ancestry Group/ OR Indian, North American/ OR American Indians/ OR Alaska Natives/ OR Indigenous Canadians/ OR Inuits/ OR Native Hawaiian/ OR Other Pacific Islander/ OR

**TW**: Aboriginal OR “Torres Strait” OR Maori OR Ainu OR Sami

AND

**MeSH:** Blogging/ OR social media/ OR electronic mail/ OR internet/ access OR internet use/ OR internet-based intervention/ OR social networking/ OR online social networking/ OR Smartphone/ OR

**TW:** Facebook OR SnapChat OR Instagram OR Twitter OR TicToc OR Linkedin OR Pinterest OR youtube

AND

**MeSH:** consumer health information/ OR health literacy/ OR health promotion/ or healthy people programs/ OR weight reduction programs/ OR sex education/ OR /smoking prevention/ OR Quality of Life/ OR Self-Care/ OR neoplasmsn OR lung diseases/ OR chronic disease/ Or multiple chronic conditions OR Cardiovascular Diseases/ OR diabetes mellitus/ OR **Stress, Psychological/** OR alcoholism/ OR smoking reduction/ OR cigarette smoking/ OR vaping OR Substance-Related Disorders/

**TW:** wellbeing OR well-being or wellness

## Actual search terms for PubMed

"american native continental ancestry group"[MeSH Terms] OR ("american"[All Fields] AND "native"[All Fields] AND "continental"[All Fields] AND "ancestry"[All Fields] AND "group"[All Fields]) OR "american native continental ancestry group"[All Fields] OR ("indians, north american"[MeSH Terms] OR ("indians"[All Fields] AND "north"[All Fields] AND "american"[All Fields]) OR "north american indians"[All Fields] OR ("indian"[All Fields] AND "north"[All Fields] AND "american"[All Fields]) OR "indian north american"[All Fields]) OR ("american indians or alaska natives"[MeSH Terms] OR ("american"[All Fields] AND "indians"[All Fields] AND "or"[All Fields] AND "alaska"[All Fields] AND "natives"[All Fields]) OR "american indians or alaska natives"[All Fields] OR ("american"[All Fields] AND "indians"[All Fields]) OR "american indians"[All Fields]) OR ("alaskan natives"[MeSH Terms] OR ("alaskan"[All Fields] AND "natives"[All Fields]) OR "alaskan natives"[All Fields] OR ("alaska"[All Fields] AND "natives"[All Fields]) OR "alaska natives"[All Fields]) OR ("indigenous canadians"[MeSH Terms] OR ("indigenous"[All Fields] AND "canadians"[All Fields]) OR "indigenous canadians"[All Fields]) OR ("inuits"[MeSH Terms] OR "inuits"[All Fields] OR "inuit"[All Fields]) OR ("native hawaiian or other pacific islander"[MeSH Terms] OR ("native"[All Fields] AND "hawaiian"[All Fields] AND "or"[All Fields] AND "Other"[All Fields] AND "pacific"[All Fields] AND "islander"[All Fields]) OR "native hawaiian or other pacific islander"[All Fields] OR ("native"[All Fields] AND "hawaiian"[All Fields]) OR "native hawaiian"[All Fields]) OR ("Other"[All Fields] AND "native hawaiian or other pacific islander"[MeSH Terms]) OR ("Aboriginal"[Text Word] OR "Torres Strait"[Text Word] OR "Maori"[Text Word] OR "Ainu"[Text Word] OR "Sami"[Text Word]) = 44,547

AND

"blogged"[All Fields] OR "blogging"[MeSH Terms] OR "blogging"[All Fields] OR "blogs"[All Fields] OR ("social media"[MeSH Terms] OR ("social"[All Fields] AND "media"[All Fields]) OR "social media"[All Fields]) OR ("electronic mail"[MeSH Terms] OR ("electronic"[All Fields] AND "mail"[All Fields]) OR "electronic mail"[All Fields]) OR ("internet access"[MeSH Terms] OR ("internet"[All Fields] AND "access"[All Fields]) OR "internet access"[All Fields]) OR ("internet use"[MeSH Terms] OR "internet"[All Fields] OR "internet use"[All Fields]) OR ("internet based intervention"[MeSH Terms] OR ("internet based"[All Fields] AND "intervention"[All Fields]) OR "internet based intervention"[All Fields] OR ("internet"[All Fields] AND "based"[All Fields] AND "intervention"[All Fields]) OR "internet based intervention"[All Fields]) OR ("social networking"[MeSH Terms] OR ("social"[All Fields] AND "networking"[All Fields]) OR "social networking"[All Fields]) OR ("online social networking"[MeSH Terms] OR ("online"[All Fields] AND "social"[All Fields] AND "networking"[All Fields]) OR "online social networking"[All Fields]) OR "smartphone"[MeSH Terms] OR ("Facebook"[Text Word] OR "SnapChat"[Text Word] OR "Instagram"[Text Word] OR "Twitter"[Text Word] OR "TicToc"[Text Word] OR "Linkedin"[Text Word] OR "Pinterest"[Text Word] OR "youtube"[Text Word]) = 218,370

AND

"consumer health information"[MeSH Terms] OR ("consumer"[All Fields] AND "health"[All Fields] AND "information"[All Fields]) OR "consumer health information"[All Fields] OR ("health literacy"[MeSH Terms] OR ("health"[All Fields] AND "literacy"[All Fields]) OR "health literacy"[All Fields]) OR ("health promotion"[MeSH Terms] OR ("health"[All Fields] AND "promotion"[All Fields]) OR "health promotion"[All Fields]) OR ("healthy people programmes"[All Fields] OR "healthy people programs"[MeSH Terms] OR ("healthy"[All Fields] AND "people"[All Fields] AND "programs"[All Fields]) OR "healthy people programs"[All Fields]) OR ("weight reduction programs"[MeSH Terms] OR ("weight"[All Fields] AND "reduction"[All Fields] AND "programs"[All Fields]) OR "weight reduction programs"[All Fields]) OR ("sex education"[MeSH Terms] OR ("sex"[All Fields] AND "education"[All Fields]) OR "sex education"[All Fields]) OR ("smoking prevention"[MeSH Terms] OR ("smoking"[All Fields] AND "prevention"[All Fields]) OR "smoking prevention"[All Fields]) OR ("quality of life"[MeSH Terms] OR ("quality"[All Fields] AND "life"[All Fields]) OR "quality of life"[All Fields]) OR ("self care"[MeSH Terms] OR ("self"[All Fields] AND "care"[All Fields]) OR "self care"[All Fields]) OR "neoplasmsn"[All Fields] OR ("lung diseases"[MeSH Terms] OR ("lung"[All Fields] AND "diseases"[All Fields]) OR "lung diseases"[All Fields]) OR (("chronic disease"[MeSH Terms] OR ("chronic"[All Fields] AND "disease"[All Fields]) OR "chronic disease"[All Fields]) AND "Or"[All Fields] AND ("multiple chronic conditions"[MeSH Terms] OR ("multiple"[All Fields] AND "chronic"[All Fields] AND "conditions"[All Fields]) OR "multiple chronic conditions"[All Fields])) OR ("cardiovascular diseases"[MeSH Terms] OR ("cardiovascular"[All Fields] AND "diseases"[All Fields]) OR "cardiovascular diseases"[All Fields]) OR ("diabetes mellitus"[MeSH Terms] OR ("diabetes"[All Fields] AND "mellitus"[All Fields]) OR "diabetes mellitus"[All Fields]) OR ("stress, psychological"[MeSH Terms] OR ("stress"[All Fields] AND "psychological"[All Fields]) OR "psychological stress"[All Fields] OR ("stress"[All Fields] AND "psychological"[All Fields]) OR "stress psychological"[All Fields]) OR ("alcohol s"[All Fields] OR "alcoholate"[All Fields] OR "alcoholates"[All Fields] OR "alcoholic s"[All Fields] OR "alcoholics"[MeSH Terms] OR "alcoholics"[All Fields] OR "alcoholic"[All Fields] OR "alcoholism"[MeSH Terms] OR "alcoholism"[All Fields] OR "alcoholisms"[All Fields] OR "alcoholism s"[All Fields] OR "alcoholization"[All Fields] OR "alcohols"[MeSH Terms] OR "alcohols"[All Fields] OR "ethanol"[MeSH Terms] OR "ethanol"[All Fields] OR "alcohol"[All Fields]) OR ("smoking reduction"[MeSH Terms] OR ("smoking"[All Fields] AND "reduction"[All Fields]) OR "smoking reduction"[All Fields]) OR ("cigarette smoking"[MeSH Terms] OR ("cigarette"[All Fields] AND "smoking"[All Fields]) OR "cigarette smoking"[All Fields]) OR ("vaped"[All Fields] OR "vaping"[MeSH Terms] OR "vaping"[All Fields] OR "vapes"[All Fields]) OR "substance related disorders"[MeSH Terms] OR ("wellbeing"[Text Word] OR "well-being"[Text Word] OR "wellness"[Text Word]) = 6,163,878

AND

Filters: Adult: 19-44 years, Middle Aged + Aged: 45+ years, Middle Aged: 45-64 years, Aged: 65+ years, 80 and over: 80+ years, from 2005/1/1 - 2022/12/31 = 126

## Link to search strategy for PubMed

**https://pubmed.ncbi.nlm.nih.gov/searches/6395640/?mode=full&sort=date**

## References for PubMed

1. Wright M, Brown A, Dudgeon P, et al. Our journey, our story: a study protocol for the evaluation of a co-design framework to improve services for Aboriginal youth mental health and well-being. *BMJ Open* 2021; 11: e042981. 20210519. DOI: 10.1136/bmjopen-2020-042981.

2. Wong G, Glover M, McPherson M, et al. Boosting efficacy of nurse-led stop smoking interventions with a quit and win contest: pilot study results. *Contemp Nurse* 2018; 54: 395-408. 20181101. DOI: 10.1080/10376178.2018.1539337.

3. Wilkins C, Romeo JS, Rychert M, et al. Determinants of high availability of methamphetamine, cannabis, LSD and ecstasy in New Zealand: Are drug dealers promoting methamphetamine rather than cannabis? *Int J Drug Policy* 2018; 61: 15-22. 20181019. DOI: 10.1016/j.drugpo.2018.09.007.

4. Whittaker R, Dorey E, Bramley D, et al. A theory-based video messaging mobile phone intervention for smoking cessation: randomized controlled trial. *J Med Internet Res* 2011; 13: e10. 20110121. DOI: 10.2196/jmir.1553.

5. Whittaker R, Bramley D, Wells S, et al. Will a web-based cardiovascular disease (CVD) risk assessment programme increase the assessment of CVD risk factors for Maori? *N Z Med J* 2006; 119: U2077. 20060721.

6. Whealin JM, Nelson D, Kawasaki MM, et al. Factors impacting rural Pacific Island veterans' access to care: A qualitative examination. *Psychol Serv* 2017; 14: 279-288. DOI: 10.1037/ser0000161.

7. Wells S, Kerr A, Broad J, et al. The impact of New Zealand CVD risk chart adjustments for family history and ethnicity on eligibility for treatment (PREDICT CVD-5). *N Z Med J* 2007; 120: U2712. 20070907.

8. Wan N, Wen M, Fan JX, et al. Physical Activity Barriers and Facilitators Among US Pacific Islanders and the Feasibility of Using Mobile Technologies for Intervention: A Focus Group Study With Tongan Americans. *J Phys Act Health* 2018; 15: 287-294. 20171204. DOI: 10.1123/jpah.2017-0014.

9. Walker SL, Saltman DL, Colucci R, et al. Awareness of risk factors among persons at risk for lung cancer, chronic obstructive pulmonary disease and sleep apnea: a Canadian population-based study. *Can Respir J* 2010; 17: 287-294. DOI: 10.1155/2010/426563.

10. Walker N, Smith B, Barnes J, et al. Cytisine versus varenicline for smoking cessation in New Zealand indigenous Māori: a randomized controlled trial. *Addiction* 2021; 116: 2847-2858. 20210504. DOI: 10.1111/add.15489.

11. Volkova E, Michie J, Corrigan C, et al. Effectiveness of recruitment to a smartphone-delivered nutrition intervention in New Zealand: analysis of a randomised controlled trial. *BMJ Open* 2017; 7: e016198. 20170702. DOI: 10.1136/bmjopen-2017-016198.

12. Tumiel-Berhalter LM, Kahn L, Watkins R, et al. The implementation of Good For The Neighborhood: a participatory community health program model in four minority underserved communities. *J Community Health* 2011; 36: 669-674. DOI: 10.1007/s10900-011-9358-6.

13. Tran JH, Mouttapa M, Ichinose TY, et al. Sources of information that promote breast and cervical cancer knowledge and screening among native Hawaiians in Southern California. *J Cancer Educ* 2010; 25: 588-594. DOI: 10.1007/s13187-010-0078-x.

14. Tomayko EJ, Prince RJ, Cronin KA, et al. Healthy Children, Strong Families 2: A randomized controlled trial of a healthy lifestyle intervention for American Indian families designed using community-based approaches. *Clin Trials* 2017; 14: 152-161. 20170109. DOI: 10.1177/1740774516685699.

15. Titov N, Dear BF, Staples LG, et al. The first 30 months of the MindSpot Clinic: Evaluation of a national e-mental health service against project objectives. *Aust N Z J Psychiatry* 2017; 51: 1227-1239. 20161012. DOI: 10.1177/0004867416671598.

16. Thomas DP, Davey ME, van der Sterren AE, et al. Social networks and quitting in a national cohort of Australian Aboriginal and Torres Strait Islander smokers. *Drug Alcohol Rev* 2019; 38: 82-91. 20190113. DOI: 10.1111/dar.12891.

17. Stotz S, Brega AG, Lockhart S, et al. An online diabetes nutrition education programme for American Indian and Alaska Native adults with type 2 diabetes: perspectives from key stakeholders. *Public Health Nutr* 2021; 24: 1449-1459. 20200717. DOI: 10.1017/s1368980020001743.

18. Stockman JK, Wood BA and Anderson KM. Racial and Ethnic Differences in COVID-19 Outcomes, Stressors, Fear, and Prevention Behaviors Among US Women: Web-Based Cross-sectional Study. *J Med Internet Res* 2021; 23: e26296. 20210712. DOI: 10.2196/26296.

19. Stewart J, Krows ML, Schaafsma TT, et al. Comparison of Racial, Ethnic, and Geographic Location Diversity of Participants Enrolled in Clinic-Based vs 2 Remote COVID-19 Clinical Trials. *JAMA Netw Open* 2022; 5: e2148325. 20220201. DOI: 10.1001/jamanetworkopen.2021.48325.

20. Snyder M and Wilson K. "Too much moving...there's always a reason": Understanding urban Aboriginal peoples' experiences of mobility and its impact on holistic health. *Health Place* 2015; 34: 181-189. 20150606. DOI: 10.1016/j.healthplace.2015.05.009.

21. Smith JA, Merlino A, Christie B, et al. 'Dudes Are Meant to be Tough as Nails': The Complex Nexus Between Masculinities, Culture and Health Literacy From the Perspective of Young Aboriginal and Torres Strait Islander Males - Implications for Policy and Practice. *Am J Mens Health* 2020; 14: 1557988320936121. DOI: 10.1177/1557988320936121.

22. Smartlowit-Briggs L, Pearson C, Whitefoot P, et al. Community-Based Assessment to Inform a Chlamydia Screening Program for Women in a Rural American Indian Community. *Sex Transm Dis* 2016; 43: 390-395. DOI: 10.1097/olq.0000000000000456.

23. Sinka V, Lopez-Vargas P, Tong A, et al. Chronic disease prevention programs offered by Aboriginal Community Controlled Health Services in New South Wales, Australia. *Aust N Z J Public Health* 2021; 45: 59-64. DOI: 10.1111/1753-6405.13069.

24. Shiels MS, Chernyavskiy P, Anderson WF, et al. Trends in premature mortality in the USA by sex, race, and ethnicity from 1999 to 2014: an analysis of death certificate data. *Lancet* 2017; 389: 1043-1054. 20170126. DOI: 10.1016/s0140-6736(17)30187-3.

25. Saxton PJW, Azariah S, Franklin RA, et al. Baseline characteristics of gay and bisexual men in a HIV pre-exposure prophylaxis demonstration project with equity quotas in Auckland, New Zealand. *Sex Health* 2019; 16: 47-55. DOI: 10.1071/sh18056.

26. Sacca L, Craig Rushing S, Markham C, et al. Assessment of the Reach, Usability, and Perceived Impact of "Talking Is Power": A Parental Sexual Health Text-Messaging Service and Web-Based Resource to Empower Sensitive Conversations with American Indian and Alaska Native Teens. *Int J Environ Res Public Health* 2021; 18 20210830. DOI: 10.3390/ijerph18179126.

27. Running Bear U, Beals J, Novins DK, et al. Alcohol detoxification completion, acceptance of referral to substance abuse treatment, and entry into substance abuse treatment among Alaska Native people. *Addict Behav* 2017; 65: 25-32. 20160924. DOI: 10.1016/j.addbeh.2016.09.009.

28. Running Bear U, Anderson H, Manson SM, et al. Impact of adaptive functioning on readmission to alcohol detoxification among Alaska Native People. *Drug Alcohol Depend* 2014; 140: 168-174. 20140428. DOI: 10.1016/j.drugalcdep.2014.04.018.

29. Rucas SL, Gurven M, Winking J, et al. Social aggression and resource conflict across the female life-course in the Bolivian Amazon. *Aggress Behav* 2012; 38: 194-207. DOI: 10.1002/ab.21420.

30. Robinson JD, Turner JW, Levine B, et al. Expanding the walls of the health care encounter: support and outcomes for patients online. *Health Commun* 2011; 26: 125-134. DOI: 10.1080/10410236.2010.541990.

31. Riddell T, Jackson RT, Wells S, et al. Assessing Māori/non-Māori differences in cardiovascular disease risk and risk management in routine primary care practice using web-based clinical decision support: (PREDICT CVD-2). *N Z Med J* 2007; 120: U2445. 20070302.

32. Reti SR, Feldman HJ and Safran C. Online access and literacy in Maori New Zealanders with diabetes. *J Prim Health Care* 2011; 3: 190-191. 20110901.

33. Ramraj C, Shahidi FV, Darity W, Jr., et al. Equally inequitable? A cross-national comparative study of racial health inequalities in the United States and Canada. *Soc Sci Med* 2016; 161: 19-26. 20160518. DOI: 10.1016/j.socscimed.2016.05.028.

34. Ram FS and McNaughton W. Giving Asthma Support to Patients (GASP): a novel online asthma education, monitoring, assessment and management tool. *J Prim Health Care* 2014; 6: 238-244. 20140901.

35. Povey J, Mills PP, Dingwall KM, et al. Acceptability of Mental Health Apps for Aboriginal and Torres Strait Islander Australians: A Qualitative Study. *J Med Internet Res* 2016; 18: e65. 20160311. DOI: 10.2196/jmir.5314.

36. Pokhrel P, Ing C, Kawamoto CT, et al. Social media's influence on e-cigarette use onset and escalation among young adults: What beliefs mediate the effects? *Addict Behav* 2021; 112: 106617. 20200819. DOI: 10.1016/j.addbeh.2020.106617.

37. Palen LA, Ashley OS, Gard JC, et al. Effects of media campaign messages targeting parents on adolescent sexual beliefs: a randomized controlled trial with a national sample. *Fam Community Health* 2011; 34: 28-38. DOI: 10.1097/FCH.0b013e3181fdecc3.

38. Okamoto SK, Helm S, Delp JA, et al. A community stakeholder analysis of drug resistance strategies of rural native Hawaiian youth. *J Prim Prev* 2011; 32: 185-193. DOI: 10.1007/s10935-011-0247-7.

39. Ni Mhurchu C, Te Morenga L, Tupai-Firestone R, et al. A co-designed mHealth programme to support healthy lifestyles in Māori and Pasifika peoples in New Zealand (OL@-OR@): a cluster-randomised controlled trial. *Lancet Digit Health* 2019; 1: e298-e307. 20190917. DOI: 10.1016/s2589-7500(19)30130-x.

40. Ni Mhurchu C, Eyles H, Dixon R, et al. Economic incentives to promote healthier food purchases: exploring acceptability and key factors for success. *Health Promot Int* 2012; 27: 331-341. 20110708. DOI: 10.1093/heapro/dar042.

41. Ni Mhurchu C, Blakely T, Wall J, et al. Strategies to promote healthier food purchases: a pilot supermarket intervention study. *Public Health Nutr* 2007; 10: 608-615. 20070305. DOI: 10.1017/s136898000735249x.

42. Nguyen AB, Robinson J, O'Brien EK, et al. Racial and Ethnic Differences in Tobacco Information Seeking and Information Sources: Findings From the 2015 Health Information National Trends Survey. *J Health Commun* 2017; 22: 743-752. 20170801. DOI: 10.1080/10810730.2017.1347216.

43. Nghiem N, Leung W, Cleghorn C, et al. Mass media promotion of a smartphone smoking cessation app: modelled health and cost-saving impacts. *BMC Public Health* 2019; 19: 283. 20190308. DOI: 10.1186/s12889-019-6605-8.

44. Nghiem N, Cleghorn CL, Leung W, et al. A national quitline service and its promotion in the mass media: modelling the health gain, health equity and cost-utility. *Tob Control* 2018; 27: 434-441. 20170724. DOI: 10.1136/tobaccocontrol-2017-053660.

45. Newby JM, O'Moore K, Tang S, et al. Acute mental health responses during the COVID-19 pandemic in Australia. *PLoS One* 2020; 15: e0236562. 20200728. DOI: 10.1371/journal.pone.0236562.

46. Minde GT and Sæterstrand TM. What is important in the surroundings in order to extend the healthy life period? A regional study of 19 older women in a northern part of Norway. *Int J Circumpolar Health* 2013; 72 20130805. DOI: 10.3402/ijch.v72i0.21189.

47. Miller BL and Stogner JM. Not-so-clean fun: a profile of bath salt users among a college sample in the United States. *J Psychoactive Drugs* 2014; 46: 147-153. DOI: 10.1080/02791072.2013.876520.

48. Mikhail H, Kelly SE and Davison CM. Reproductive health interventions for Inuit youth in the north: a scoping review. *Reprod Health* 2021; 18: 65. 20210320. DOI: 10.1186/s12978-021-01119-6.

49. Merculieff ZT, Koller KR, Sinicrope PS, et al. Developing a Social Media Intervention to Connect Alaska Native People Who Smoke with Resources and Support to Quit Smoking: The Connecting Alaska Native Quit Study. *Nicotine Tob Res* 2021; 23: 1002-1009. DOI: 10.1093/ntr/ntaa253.

50. Mead EL, Gittelsohn J, Roache C, et al. A community-based, environmental chronic disease prevention intervention to improve healthy eating psychosocial factors and behaviors in indigenous populations in the Canadian Arctic. *Health Educ Behav* 2013; 40: 592-602. 20121213. DOI: 10.1177/1090198112467793.

51. McElfish PA, Rowland B, Riklon S, et al. Development and Evaluation of a Blood Glucose Monitoring YouTube Video for Marshallese Patients Using a Community-Based Participatory Research Approach. *Policy Polit Nurs Pract* 2019; 20: 205-215. 20190919. DOI: 10.1177/1527154419872834.

52. McDonald E, Cunningham T and Slavin N. Evaluating a handwashing with soap program in Australian remote Aboriginal communities: a pre and post intervention study design. *BMC Public Health* 2015; 15: 1188. 20151127. DOI: 10.1186/s12889-015-2503-x.

53. Mathieson K, Leafman JS and Horton MB. Access to Digital Communication Technology and Perceptions of Telemedicine for Patient Education among American Indian Patients with Diabetes. *J Health Care Poor Underserved* 2017; 28: 1522-1536. DOI: 10.1353/hpu.2017.0131.

54. Malerbi FK, Dal Fabbro AL, Moises RCS, et al. High Frequency of Asteroid Hyalosis Precludes Diabetic Retinopathy Screening With Smartphone-Based Retinal Camera in Brazilian Xavante Indians. *J Diabetes Sci Technol* 2020; 14: 974-975. 20200529. DOI: 10.1177/1932296820929673.

55. Lorig K, Ritter PL, Laurent DD, et al. Online diabetes self-management program: a randomized study. *Diabetes Care* 2010; 33: 1275-1281. 20100318. DOI: 10.2337/dc09-2153.

56. Lopez JM, Bailey RA, Rupnow MF, et al. Characterization of type 2 diabetes mellitus burden by age and ethnic groups based on a nationwide survey. *Clin Ther* 2014; 36: 494-506. 20140205. DOI: 10.1016/j.clinthera.2013.12.016.

57. Lofti-Jam KL, O'Reilly CL, Feng CS, et al. Increasing bowel cancer screening participation: integrating population-wide, primary care and more targeted approaches. *Public Health Res Pract* 2019; 29 20190731. DOI: 10.17061/phrp2921916.

58. Lemelin H, Matthews D, Mattina C, et al. Climate change, wellbeing and resilience in the Weenusk First Nation at Peawanuck: the Moccasin Telegraph goes global. *Rural Remote Health* 2010; 10: 1333. 20100618.

59. Lee KSK, Fitts MS, Conigrave JH, et al. Recruiting a representative sample of urban South Australian Aboriginal adults for a survey on alcohol consumption. *BMC Med Res Methodol* 2020; 20: 183. 20200706. DOI: 10.1186/s12874-020-01067-y.

60. Lapointe-Shaw L, Rader B, Astley CM, et al. Web and phone-based COVID-19 syndromic surveillance in Canada: A cross-sectional study. *PLoS One* 2020; 15: e0239886. 20201002. DOI: 10.1371/journal.pone.0239886.

61. Lai HT, Kung PT, Su HP, et al. Examining related influential factors for dental calculus scaling utilization among people with disabilities in Taiwan, a nationwide population-based study. *Res Dev Disabil* 2014; 35: 2231-2240. 20140611. DOI: 10.1016/j.ridd.2014.05.014.

62. Kypri K, McCambridge J, Vater T, et al. Web-based alcohol intervention for Māori university students: double-blind, multi-site randomized controlled trial. *Addiction* 2013; 108: 331-338. 20121107. DOI: 10.1111/j.1360-0443.2012.04067.x.

63. Kypri K, Langley J and Stephenson S. Episode-centred analysis of drinking to intoxication in university students. *Alcohol Alcohol* 2005; 40: 447-452. 20050704. DOI: 10.1093/alcalc/agh178.

64. Kyoon-Achan G, Schroth RJ, Sanguins J, et al. Early childhood oral health promotion for First Nations and Métis communities and caregivers in Manitoba. *Health Promot Chronic Dis Prev Can* 2021; 41: 14-24. DOI: 10.24095/hpcdp.41.1.02.

65. Koziol-McLain J, McLean C, Rohan M, et al. Participant Recruitment and Engagement in Automated eHealth Trial Registration: Challenges and Opportunities for Recruiting Women Who Experience Violence. *J Med Internet Res* 2016; 18: e281. 20161025. DOI: 10.2196/jmir.6515.

66. Korn Malerbi F, Lelis Dal Fabbro A, Botelho Vieira Filho JP, et al. The feasibility of smartphone based retinal photography for diabetic retinopathy screening among Brazilian Xavante Indians. *Diabetes Res Clin Pract* 2020; 168: 108380. 20200821. DOI: 10.1016/j.diabres.2020.108380.

67. Knox M, Skan J, Benowitz NL, et al. Recruitment best practices of a cardiovascular risk reduction randomised control trial in rural Alaska Native communities. *Int J Circumpolar Health* 2020; 79: 1806639. DOI: 10.1080/22423982.2020.1806639.

68. Kira A, Glover M, Walker N, et al. Recruiting Pregnant Indigenous Women Who Smoke into a High Contact Incentivized Cessation Trial: A Feasibility Study. *Nicotine Tob Res* 2016; 18: 2036-2040. 20160506. DOI: 10.1093/ntr/ntw106.

69. Khalil Zadeh N, Robertson K and Green JA. 'At-risk' individuals' responses to direct to consumer advertising of prescription drugs: a nationally representative cross-sectional study. *BMJ Open* 2017; 7: e017865. 20171206. DOI: 10.1136/bmjopen-2017-017865.

70. Kerestes C, Delafield R, Elia J, et al. "It was close enough, but it wasn't close enough": A qualitative exploration of the impact of direct-to-patient telemedicine abortion on access to abortion care. *Contraception* 2021; 104: 67-72. 20210430. DOI: 10.1016/j.contraception.2021.04.028.

71. Kelley A, Steinberg R, McCoy TP, et al. Exploring recovery: Findings from a six-year evaluation of an American Indian peer recovery support program. *Drug Alcohol Depend* 2021; 221: 108559. 20210129. DOI: 10.1016/j.drugalcdep.2021.108559.

72. Kearns N, Shortt N, Kearns C, et al. How big is your bubble? Characteristics of self-isolating household units ('bubbles') during the COVID-19 Alert Level 4 period in New Zealand: a cross-sectional survey. *BMJ Open* 2021; 11: e042464. 20210128. DOI: 10.1136/bmjopen-2020-042464.

73. Kay-Lambkin FJ, White A, Baker AL, et al. Assessment of function and clinical utility of alcohol and other drug web sites: an observational, qualitative study. *BMC Public Health* 2011; 11: 277. 20110505. DOI: 10.1186/1471-2458-11-277.

74. Kamimura A, Ashby J, Jess A, et al. Impact of Neighborhood Environments on Health Consciousness, Information Seeking, and Attitudes among US-Born and Non-US-Born Free Clinic Patients. *South Med J* 2015; 108: 703-709. DOI: 10.14423/smj.0000000000000379.

75. Jongbloed K, Thomas V, Pearce ME, et al. The Cedar Project: Residential transience and HIV vulnerability among young Aboriginal people who use drugs. *Health Place* 2015; 33: 125-131. 20150402. DOI: 10.1016/j.healthplace.2015.02.008.

76. Jones EJ, Peercy M, Woods JC, et al. Identifying postpartum intervention approaches to reduce cardiometabolic risk among American Indian women with prior gestational diabetes, Oklahoma, 2012-2013. *Prev Chronic Dis* 2015; 12: E45. 20150402. DOI: 10.5888/pcd12.140566.

77. Jernigan VB and Lorig K. The internet diabetes self-management workshop for American Indians and Alaska Natives. *Health Promot Pract* 2011; 12: 261-270. 20100609. DOI: 10.1177/1524839909335178.

78. Jeffs E, Williman J, Brunton C, et al. Pregnant women's knowledge of, and adherence to, New Zealand Food Safety in Pregnancy guidelines. *N Z Med J* 2020; 133: 41-52. 20201120.

79. Imlach F, McKinlay E, Middleton L, et al. Telehealth consultations in general practice during a pandemic lockdown: survey and interviews on patient experiences and preferences. *BMC Fam Pract* 2020; 21: 269. 20201213. DOI: 10.1186/s12875-020-01336-1.

80. Howe LD, Ellison-Loschmann L, Pearce N, et al. Ethnic differences in risk factors for obesity in New Zealand infants. *J Epidemiol Community Health* 2015; 69: 516-522. 20150320. DOI: 10.1136/jech-2014-204464.

81. Hoffman-Goetz L and Donelle L. Chat room computer-mediated support on health issues for aboriginal women. *Health Care Women Int* 2007; 28: 397-418. DOI: 10.1080/07399330601180057.

82. Hildebrand J, Burns S, Zhao Y, et al. Potential and Challenges in Collecting Social and Behavioral Data on Adolescent Alcohol Norms: Comparing Respondent-Driven Sampling and Web-Based Respondent-Driven Sampling. *J Med Internet Res* 2015; 17: e285. 20151224. DOI: 10.2196/jmir.4762.

83. Henderson JA, Chubak J, O'Connell J, et al. Design of a randomized controlled trial of a web-based intervention to reduce cardiovascular disease risk factors among remote reservation-dwelling American Indian adults with type 2 diabetes. *J Prim Prev* 2012; 33: 209-222. DOI: 10.1007/s10935-012-0276-x.

84. Hefler M, Kerrigan V, Henryks J, et al. Social media and health information sharing among Australian Indigenous people. *Health Promot Int* 2019; 34: 706-715. DOI: 10.1093/heapro/day018.

85. Hefler M, Kerrigan V, Freeman B, et al. Using Facebook to reduce smoking among Australian Aboriginal and Torres Strait Islander people: a participatory grounded action study. *BMC Public Health* 2019; 19: 615. 20190521. DOI: 10.1186/s12889-019-6918-7.

86. Hanson JD, Winberg A and Elliott A. Development of a media campaign on fetal alcohol spectrum disorders for Northern Plains American Indian communities. *Health Promot Pract* 2012; 13: 842-847. 20111212. DOI: 10.1177/1524839911404232.

87. Hamby S, Schultz K and Elm J. Understanding the burden of trauma and victimization among American Indian and Alaska native elders: historical trauma as an element of poly-victimization. *J Trauma Dissociation* 2020; 21: 172-186. 20191121. DOI: 10.1080/15299732.2020.1692408.

88. Gwynne K, Flaskas Y, O'Brien C, et al. Opportunistic screening to detect atrial fibrillation in Aboriginal adults in Australia. *BMJ Open* 2016; 6: e013576. 20161115. DOI: 10.1136/bmjopen-2016-013576.

89. Gu Y, Warren J, Kennelly J, et al. Cardiovascular disease risk management for Māori in New Zealand general practice. *J Prim Health Care* 2014; 6: 286-294. 20141201.

90. Grigg M, Waa A and Bradbrook SK. Response to an indigenous smoking cessation media campaign - it's about whānau. *Aust N Z J Public Health* 2008; 32: 559-564. DOI: 10.1111/j.1753-6405.2008.00310.x.

91. Graham AL and Papandonatos GD. Reliability of internet- versus telephone-administered questionnaires in a diverse sample of smokers. *J Med Internet Res* 2008; 10: e8. 20080326. DOI: 10.2196/jmir.987.

92. Gould GS, Zeev YB, Tywman L, et al. Do Clinicians Ask Pregnant Women about Exposures to Tobacco and Cannabis Smoking, Second-Hand-Smoke and E-Cigarettes? An Australian National Cross-Sectional Survey. *Int J Environ Res Public Health* 2017; 14 20171216. DOI: 10.3390/ijerph14121585.

93. Gorman JR, Clapp JD, Calac D, et al. Creating a culturally appropriate web-based behavioral intervention for American Indian/Alaska Native women in Southern California: the healthy women healthy Native nation study. *Am Indian Alsk Native Ment Health Res* 2013; 20: 1-15. DOI: 10.5820/aian.2001.2013.1.

94. Glover M, Kira A, Gentles D, et al. The WERO group stop smoking competition: main outcomes of a pre- and post- study. *BMC Public Health* 2014; 14: 599. 20140613. DOI: 10.1186/1471-2458-14-599.

95. Gittelsohn J, Jock B, Poirier L, et al. Implementation of a multilevel, multicomponent intervention for obesity control in Native American communities (OPREVENT2): challenges and lessons learned. *Health Educ Res* 2020; 35: 228-242. DOI: 10.1093/her/cyaa012.

96. Gilmour J, Strong A, Hawkins M, et al. Nurses and heart failure education in medical wards. *Nurs Prax N Z* 2013; 29: 5-17.

97. Gillis J, Bayoumi AM, Burchell AN, et al. Factors associated with the frequency of monitoring of liver enzymes, renal function and lipid laboratory markers among individuals initiating combination antiretroviral therapy: a cohort study. *BMC Infect Dis* 2015; 15: 453. 20151026. DOI: 10.1186/s12879-015-1206-3.

98. Geana MV, Greiner KA, Cully A, et al. Improving health promotion to American Indians in the midwest United States: preferred sources of health information and its use for the medical encounter. *J Community Health* 2012; 37: 1253-1263. DOI: 10.1007/s10900-012-9564-x.

99. Friedman DB, Laditka JN, Hunter R, et al. Getting the message out about cognitive health: a cross-cultural comparison of older adults' media awareness and communication needs on how to maintain a healthy brain. *Gerontologist* 2009; 49 Suppl 1: S50-60. DOI: 10.1093/geront/gnp080.

100. Foulds HJA, Bredin SSD and Warburton DER. Ethnic differences in vascular function and factors contributing to blood pressure. *Can J Public Health* 2018; 109: 316-326. 20180511. DOI: 10.17269/s41997-018-0076-z.

101. Fahrenwald NL and Stabnow W. Sociocultural perspective on organ and tissue donation among reservation-dwelling American Indian adults. *Ethn Health* 2005; 10: 341-354. DOI: 10.1080/13557850500168826.

102. Duncan KC, Salters K, Forrest JI, et al. Cohort Profile: Longitudinal Investigations into Supportive and Ancillary health services. *Int J Epidemiol* 2013; 42: 947-955. 20120329. DOI: 10.1093/ije/dys035.

103. Dowsett M, Islam MM, Ganora C, et al. Asking young Aboriginal people who use illicit drugs about their healthcare preferences using audio-computer-assisted self-interviewing. *Drug Alcohol Rev* 2019; 38: 482-493. DOI: 10.1111/dar.12957.

104. Dotson JA, Nelson LA, Young SL, et al. Use of cell phones and computers for health promotion and tobacco cessation by American Indian college students in Montana. *Rural Remote Health* 2017; 17: 4014. 20170322. DOI: 10.22605/rrh4014.

105. Desrosiers A, Vine V and Kershaw T. "R U Mad?": Computerized text analysis of affect in social media relates to stress and substance use among ethnic minority emerging adult males. *Anxiety Stress Coping* 2019; 32: 109-123. 20181029. DOI: 10.1080/10615806.2018.1539964.

106. D'Amico EJ, Dickerson DL, Rodriguez A, et al. Integrating traditional practices and social network visualization to prevent substance use: study protocol for a randomized controlled trial among urban Native American emerging adults. *Addict Sci Clin Pract* 2021; 16: 56. 20210926. DOI: 10.1186/s13722-021-00265-3.

107. Cueva M, Kuhnley R, Revels LJ, et al. Bridging storytelling traditions with digital technology. *Int J Circumpolar Health* 2013; 72 20130805. DOI: 10.3402/ijch.v72i0.20717.

108. Cooley ME, Blonquist TM, Catalano PJ, et al. Feasibility of using algorithm-based clinical decision support for symptom assessment and management in lung cancer. *J Pain Symptom Manage* 2015; 49: 13-26. 20140529. DOI: 10.1016/j.jpainsymman.2014.05.003.

109. Chepulis L, Morison B, Tamatea J, et al. Midwifery awareness of diabetes in pregnancy screening guidelines in Aotearoa New Zealand. *Midwifery* 2022; 106: 103230. 20211222. DOI: 10.1016/j.midw.2021.103230.

110. Cheng VWS, Piper SE, Ottavio A, et al. Recommendations for Designing Health Information Technologies for Mental Health Drawn From Self-Determination Theory and Co-design With Culturally Diverse Populations: Template Analysis. *J Med Internet Res* 2021; 23: e23502. 20210210. DOI: 10.2196/23502.

111. Campbell AN, Turrigiano E, Moore M, et al. Acceptability of a web-based community reinforcement approach for substance use disorders with treatment-seeking American Indians/Alaska Natives. *Community Ment Health J* 2015; 51: 393-403. 20140715. DOI: 10.1007/s10597-014-9764-1.

112. Callander D, Schneider JA, Radix A, et al. Longitudinal cohort of HIV-negative transgender women of colour in New York City: protocol for the TURNNT ('Trying to Understand Relationships, Networks and Neighbourhoods among Transgender women of colour') study. *BMJ Open* 2020; 10: e032876. 20200401. DOI: 10.1136/bmjopen-2019-032876.

113. Byrne N. Internet images of the speech pathology profession. *Aust Health Rev* 2018; 42: 420-428. DOI: 10.1071/ah17033.

114. Burhansstipanov L, Krebs LU, Petereit D, et al. Reality Versus Grant Application Research "Plans". *Health Promot Pract* 2018; 19: 566-572. 20170702. DOI: 10.1177/1524839917700892.

115. Buchthal OV, Doff AL, Hsu LA, et al. Avoiding a knowledge gap in a multiethnic statewide social marketing campaign: is cultural tailoring sufficient? *J Health Commun* 2011; 16: 314-327. DOI: 10.1080/10810730.2010.535111.

116. Brodt E, Bruegl A, Miller MF, et al. The Indigenous Faculty Forum: A Longitudinal Professional Development Program to Promote the Advancement of Indigenous Faculty in Academic Medicine. *Hawaii J Health Soc Welf* 2019; 78: 8-13.

117. Brennan DJ, Lachowsky NJ, Georgievski G, et al. Online Outreach Services Among Men Who Use the Internet to Seek Sex With Other Men (MISM) in Ontario, Canada: An Online Survey. *J Med Internet Res* 2015; 17: e277. 20151209. DOI: 10.2196/jmir.4503.

118. Boyd AD, Fyfe-Johnson AL, Noonan C, et al. Communication With American Indians and Alaska Natives About Cardiovascular Disease. *Prev Chronic Dis* 2020; 17: E160. 20201217. DOI: 10.5888/pcd17.200189.

119. Bartholomew K, Zhou L, Crengle S, et al. A targeted promotional DVD fails to improve Māori and Pacific participation rates in the New Zealand bowel screening pilot: results from a pseudo-randomised controlled trial. *BMC Public Health* 2019; 19: 1245. 20190909. DOI: 10.1186/s12889-019-7582-7.

120. Bartgis J and Albright G. Online role-play simulations with emotionally responsive avatars for the early detection of Native youth psychological distress, including depression and suicidal ideation. *Am Indian Alsk Native Ment Health Res* 2016; 23: 1-27. DOI: 10.5820/aian.2302.2016.1.

121. Bar-Zeev Y, Bovill M, Bonevski B, et al. Improving smoking cessation care in pregnancy at Aboriginal Medical Services: 'ICAN QUIT in Pregnancy' step-wedge cluster randomised study. *BMJ Open* 2019; 9: e025293. 20190604. DOI: 10.1136/bmjopen-2018-025293.

122. Bannink L, Wells S, Broad J, et al. Web-based assessment of cardiovascular disease risk in routine primary care practice in New Zealand: the first 18,000 patients (PREDICT CVD-1). *N Z Med J* 2006; 119: U2313. 20061117.

123. Bagshaw P, Bagshaw S, Frampton C, et al. Pilot study of methods for assessing unmet secondary health care need in New Zealand. *N Z Med J* 2017; 130: 23-38. 20170324.

124. Atlas A, Muru-Lanning M, Moyes S, et al. Cell phone and technology use by octogenarians. *J Prim Health Care* 2020; 12: 35-40. DOI: 10.1071/hc19042.

125. Ali-Hassan H, Eloulabi R and Keethakumar A. Internet non-use among Canadian indigenous older adults: Aboriginal Peoples Survey (APS). *BMC Public Health* 2020; 20: 1554. 20201015. DOI: 10.1186/s12889-020-09659-5.

126. Albright CL, Steffen AD, Wilkens LR, et al. Effectiveness of a 12-month randomized clinical trial to increase physical activity in multiethnic postpartum women: results from Hawaii's Nā Mikimiki Project. *Prev Med* 2014; 69: 214-223. 20141005. DOI: 10.1016/j.ypmed.2014.09.019.

# Web of Science 2022.03.09

## Notes about this database search

Terms I inputted:

Aboriginal OR Indigenous OR "First Nations" OR "Torres Strait Islander" OR "Native American" OR Maori OR Sami OR Inuit OR Ainu OR "American Indian"

“Mobile application” OR Smartphone OR “Social Media” OR Facebook OR SnapChat OR Instagram OR Twitter OR TicToc OR Pinterest OR youtube OR blogging OR email OR “social networking” OR “internet use” OR “internet application” OR LinkedIn

“Consumer health information” OR “health literacy” OR “health promotion” OR “Health Education” OR “User Health” OR Disease OR “Chronic Disease” OR Illness OR Cancer OR neoplasms OR “cardiovascular disease” OR Diabetes OR “Healthy people programs” OR “Weight reduction” OR “Sex education” OR “Smoking prevention” OR “tobacco use” OR Smoking OR Vaping OR Selfcare OR “Quality of Life” OR Stress OR Wellbeing OR Coping OR Alcoholism

## Actual search terms for Web of Science

**(TS=(Aboriginal OR Indigenous OR "First Nations" OR "Torres Strait Islander" OR "Native American" OR Maori OR Sami OR Inuit OR Ainu OR "American Indian") AND TS=(“Mobile application” OR Smartphone OR “Social Media” OR Facebook OR SnapChat OR Instagram OR Twitter OR TicToc OR Pinterest OR youtube OR blogging OR email OR “social networking” OR “internet use” OR “internet application” OR LinkedIn) AND TS=(“Consumer health information” OR “health literacy” OR “health promotion” OR “Health Education” OR “User Health” OR Disease OR “Chronic Disease” OR Illness OR Cancer OR neoplasms OR “cardiovascular disease” OR Diabetes OR “Healthy people programs” OR “Weight reduction” OR “Sex education” OR “Smoking prevention” OR “tobacco use” OR Smoking OR Vaping OR Selfcare OR “Quality of Life” OR Stress OR Wellbeing OR Coping OR Alcoholism )) AND (DT==("ARTICLE" OR "REVIEW") AND CU==("AUSTRALIA" OR "USA" OR "NEW ZEALAND" OR "CANADA") AND LA==("ENGLISH")) = 116**

## Link to search strategy for Web of Science

https://www.webofscience.com/wos/woscc/summary/068ceb0e-8387-4166-8f06-3fd929c444b2-28a82954/recently-added/1

## References found by Web of Science

1. Witten K, Kearns R, Opit S, et al. Facebook as soft infrastructure: producing and performing community in a mixed tenure housing development. *HOUSING STUDIES* 2021; 36: 1345-1363. DOI: 10.1080/02673037.2020.1769035.

2. Walsh L, Hyett N, Juniper N, et al. The use of social media as a tool for stakeholder engagement in health service design and quality improvement: A scoping review. *DIGITAL HEALTH* 2021; 7. DOI: 10.1177/2055207621996870.

3. Walker T, Palermo C and Klassen K. Considering the Impact of Social Media on Contemporary Improvement of Australian Aboriginal Health: Scoping Review. *JMIR PUBLIC HEALTH AND SURVEILLANCE* 2019; 5: 64-71. DOI: 10.2196/11573.

4. Walker T, Molenaar A and Palermo C. A qualitative study exploring what it means to be healthy for young Indigenous Australians and the role of social media in influencing health behaviour. *HEALTH PROMOTION JOURNAL OF AUSTRALIA* 2021; 32: 532-540. DOI: 10.1002/hpja.391.

5. Verbiest MEA, Corrigan C, Dalhousie S, et al. Using codesign to develop a culturally tailored, behavior change mHealth intervention for indigenous and other priority communities: A case study in New Zealand. *TRANSLATIONAL BEHAVIORAL MEDICINE* 2019; 9: 720-736. DOI: 10.1093/tbm/iby093.

6. Vave R. Urban-Rural Compliance Variability to COVID-19 Restrictions of Indigenous Fijian (iTaukei) Funerals in Fiji. *ASIA-PACIFIC JOURNAL OF PUBLIC HEALTH* 2021; 33: 767-774. DOI: 10.1177/10105395211005921.

7. Trees K. Mobile media: communicating with and by Indigenous youth about alcohol. *AUSTRALIAN ABORIGINAL STUDIES* 2015: 97-106.

8. Tonkin E, Jeffs L, Wycherley TP, et al. A Smartphone App to Reduce Sugar-Sweetened Beverage Consumption Among Young Adults in Australian Remote Indigenous Communities: Design, Formative Evaluation and User-Testing. *JMIR MHEALTH AND UHEALTH* 2017; 5. DOI: 10.2196/mhealth.8651.

9. Tomayko EJ, Webber EJ, Cronin KA, et al. Use of Text Messaging and Facebook Groups to Support the Healthy Children, Strong Families 2 Healthy Lifestyle Intervention for American Indian Families. *CURRENT DEVELOPMENTS IN NUTRITION* 2021; 5: 32-39. DOI: 10.1093/cdn/nzaa110.

10. Tomayko EJ, Prince RJ, Cronin KA, et al. Healthy Children, Strong Families 2: A randomized controlled trial of a healthy lifestyle intervention for American Indian families designed using community-based approaches. *CLINICAL TRIALS* 2017; 14: 152-161. DOI: 10.1177/1740774516685699.

11. Sweet M, Geia L, Dudgeon P, et al. #IHMayDay: tweeting for empowerment and social and emotional wellbeing. *AUSTRALASIAN PSYCHIATRY* 2015; 23: 636-640. DOI: 10.1177/1039856215609762.

12. Stotz S, Brega AG, Lockhart S, et al. An online diabetes nutrition education programme for American Indian and Alaska Native adults with type 2 diabetes: perspectives from key stakeholders. *PUBLIC HEALTH NUTRITION* 2021; 24: 1449-1459. DOI: 10.1017/S1368980020001743.

13. Stockman JK, Wood BA and Anderson KM. Racial and Ethnic Differences in COVID-19 Outcomes, Stressors, Fear, and Prevention Behaviors Among US Women: Web-Based Cross-sectional Study. *JOURNAL OF MEDICAL INTERNET RESEARCH* 2021; 23. DOI: 10.2196/26296.

14. Solomon TGA, Jones D, Laurila K, et al. Using the Community Readiness Model to Assess American Indian Communities Readiness to Address Cancer Prevention and Control Programs. *JOURNAL OF CANCER EDUCATION*. DOI: 10.1007/s13187-021-02100-4.

15. Snyder T, Ravenhurst J, Cramer EY, et al. Serological surveys to estimate cumulative incidence of SARS-CoV-2 infection in adults (Sero-MAss study), Massachusetts, July-August 2020: a mail-based cross-sectional study. *BMJ OPEN* 2021; 11. DOI: 10.1136/bmjopen-2021-051157.

16. Smith JA, Merlino A, Christie B, et al. Using social media in health literacy research: A promising example involving Facebook with young Aboriginal and Torres Strait Islander males from the Top End of the Northern Territory. *HEALTH PROMOTION JOURNAL OF AUSTRALIA* 2021; 32: 186-191. DOI: 10.1002/hpja.421.

17. Smith JA, Merlino A, Christie B, et al. 'Dudes Are Meant to be Tough as Nails': The Complex Nexus Between Masculinities, Culture and Health Literacy From the Perspective of Young Aboriginal and Torres Strait Islander Males - Implications for Policy and Practice. *AMERICAN JOURNAL OF MENS HEALTH* 2020; 14. DOI: 10.1177/1557988320936121.

18. Smartlowit-Briggs L, Pearson C, Whitefoot P, et al. Community-Based Assessment to Inform a Chlamydia Screening Program for Women in a Rural American Indian Community. *SEXUALLY TRANSMITTED DISEASES* 2016; 43: 390-395. DOI: 10.1097/OLQ.0000000000000456.

19. Slater JJ and Mudryj AN. Are we really "eating well with Canada's food guide"? *BMC PUBLIC HEALTH* 2018; 18. DOI: 10.1186/s12889-018-5540-4.

20. Skinner J, Dimitropoulos Y, Moir R, et al. A graduate oral health therapist program to support dental service delivery and oral health promotion in Aboriginal communities in New South Wales, Australia. *RURAL AND REMOTE HEALTH* 2021; 21. DOI: 10.22605/RRH5789.

21. Sinka V, Lopez-Vargas P, Tong A, et al. Chronic disease prevention programs offered by Aboriginal Community Controlled Health Services in New South Wales, Australia. *AUSTRALIAN AND NEW ZEALAND JOURNAL OF PUBLIC HEALTH* 2021; 45: 59-64. DOI: 10.1111/1753-6405.13069.

22. Shibasaki S, Gardner K and Sibthorpe B. Using Knowledge Translation to Craft "Sticky" Social Media Health Messages That Provoke Interest, Raise Awareness, Impart Knowledge, and Inspire Change. *JMIR MHEALTH AND UHEALTH* 2016; 4. DOI: 10.2196/mhealth.5987.

23. Serlachius A, Schache K, Boggiss A, et al. Coping Skills Mobile App to Support the Emotional Well-Being of Young People During the COVID-19 Pandemic: Protocol for a Mixed Methods Study. *JMIR RESEARCH PROTOCOLS* 2020; 9. DOI: 10.2196/23716.

24. Scarton LJ, Bakas T, Miller WR, et al. Development and Psychometric Testing of the Diabetes Caregiver Activity and Support Scale. *DIABETES EDUCATOR* 2017; 43: 465-475. DOI: 10.1177/0145721717726280.

25. Rushing SC, Kelley A, Bull S, et al. Efficacy of an mHealth Intervention (BRAVE) to Promote Mental Wellness for American Indian and Alaska Native Teenagers and Young Adults: Randomized Controlled Trial. *JMIR MENTAL HEALTH* 2021; 8. DOI: 10.2196/26158.

26. Romero S. Bill Gates speaks K'ichee'! The corporatization of linguistic revitalization in Guatemala. *LANGUAGE & COMMUNICATION* 2016; 47: 154-166. DOI: 10.1016/j.langcom.2015.08.001.

27. Robinson RF, Dillard DA, Hiratsuka VY, et al. Formative Evaluation to Assess Communication Technology Access and Health Communication Preferences of Alaska Native People. *INTERNATIONAL JOURNAL OF INDIGENOUS HEALTH* 2015; 10: 88-101. DOI: 10.18357/ijih.102201515042.

28. Robards F, Kang M, Steinbeck K, et al. Health care equity and access for marginalised young people: a longitudinal qualitative study exploring health system navigation in Australia. *INTERNATIONAL JOURNAL FOR EQUITY IN HEALTH* 2019; 18. DOI: 10.1186/s12939-019-0941-2.

29. Rice ES, Haynes E, Royce P, et al. Social media and digital technology use among Indigenous young people in Australia: a literature review. *INTERNATIONAL JOURNAL FOR EQUITY IN HEALTH* 2016; 15. DOI: 10.1186/s12939-016-0366-0.

30. Reti SR, Feldman HJ and Safran C. Online access and literacy in Maori New Zealanders with diabetes. *JOURNAL OF PRIMARY HEALTH CARE* 2011; 3: 190-191. DOI: 10.1071/HC11190.

31. Reid P, Paine SJ, Te Ao B, et al. Estimating the economic costs of ethnic health inequities: protocol for a prevalence-based cost-of-illness study in New Zealand (2003-2014). *BMJ OPEN* 2018; 8. DOI: 10.1136/bmjopen-2017-020763.

32. Piette JD, Valverde H, Marinec N, et al. Establishing an independent mobile health program for chronic disease self-management support in Bolivia. *FRONTIERS IN PUBLIC HEALTH* 2014; 2. DOI: 10.3389/fpubh.2014.00095.

33. Peters M. How Bell Canada Capitalises on the Millennial: Affective Labour, Intersectional Identity, and Mental Health. *OPEN CULTURAL STUDIES* 2017; 1: 395-405. DOI: 10.1515/culture-2017-0037.

34. Peiris D, Wright L, News M, et al. A Smartphone App to Assist Smoking Cessation Among Aboriginal Australians: Findings From a Pilot Randomized Controlled Trial. *JMIR MHEALTH AND UHEALTH* 2019; 7. DOI: 10.2196/12745.

35. Paquin V, Sandy G, Perrault-Sullivan G, et al. Twenty "must-read" research articles for primary care providers in Nunavik: scoping study and development of an information tool. *INTERNATIONAL JOURNAL OF CIRCUMPOLAR HEALTH* 2019; 78. DOI: 10.1080/22423982.2019.1578638.

36. O'Halloran RA and Turner AW. Evaluating the impact of optical coherence tomography in diabetic retinopathy screening for an Aboriginal population. *CLINICAL AND EXPERIMENTAL OPHTHALMOLOGY* 2018; 46: 116-121. DOI: 10.1111/ceo.13018.

37. Norris P, Cousins K, Churchward M, et al. Recruiting people facing social disadvantage: the experience of the Free Meds study. *INTERNATIONAL JOURNAL FOR EQUITY IN HEALTH* 2021; 20. DOI: 10.1186/s12939-021-01483-6.

38. Nghiem N, Leung W, Cleghorn C, et al. Mass media promotion of a smartphone smoking cessation app: modelled health and cost-saving impacts. *BMC PUBLIC HEALTH* 2019; 19. DOI: 10.1186/s12889-019-6605-8.

39. Morris M. Inuit involvement in developing a participatory action research project on youth, violence prevention, and health promotion. *ETUDES INUIT STUDIES* 2016; 40: 105-125.

40. Mhurchu CN, Morenga LT, Tupai-Firestone R, et al. A co-designed mHealth programme to support healthy lifestyles in Maori and Pasifika peoples in New Zealand (OL@-OR@): a cluster-randomised controlled trial. *LANCET DIGITAL HEALTH* 2019; 1: E298-E307. DOI: 10.1016/52589-7500(19)30130-X.

41. Metheny N and Stephenson R. Structural Stressors and Intimate Partner Violence in Indigenous Men Who Have Sex With Men in the United States. *STIGMA AND HEALTH* 2020; 5: 492-496. DOI: 10.1037/sah0000220.

42. Melvin GA, Gresham D, Beaton S, et al. Evaluating the Feasibility and Effectiveness of an Australian Safety Planning Smartphone Application: A Pilot Study Within a Tertiary Mental Health Service. *SUICIDE AND LIFE-THREATENING BEHAVIOR* 2019; 49: 846-858. DOI: 10.1111/sltb.12490.

43. McPhail-Bell K, Appo N, Haymes A, et al. Deadly Choices empowering Indigenous Australians through social networking sites. *HEALTH PROMOTION INTERNATIONAL* 2018; 33: 770-780. DOI: 10.1093/heapro/dax014.

44. McConnell S. Culturally tailored postsecondary nutrition and health education curricula for indigenous populations. *INTERNATIONAL JOURNAL OF CIRCUMPOLAR HEALTH* 2013; 72: 781-786. DOI: 10.3402/ijch.v72i0.21144.

45. Mattingly JA. Fostering Cultural Safety in Nursing Education: Experiential Learning on an American Indian Reservation. *CONTEMPORARY NURSE*. DOI: 10.1080/10376178.2021.2013124.

46. Mathiyazhagan S and Wang ZM. N'KaNa-my dream: Community action towards the holistic child development in India. *CHILDREN AND YOUTH SERVICES REVIEW* 2021; 122. DOI: 10.1016/j.childyouth.2021.105924.

47. Mathieson K, Leafman JS and Horton MB. Access to Digital Communication Technology and Perceptions of Telemedicine for Patient Education among American Indian Patients with Diabetes. *JOURNAL OF HEALTH CARE FOR THE POOR AND UNDERSERVED* 2017; 28: 1522-1536. DOI: 10.1353/hpu.2017.0131.

48. Margolies DS and Strub JA. Music Community, Improvisation, and Social Technologies in COVID-Era Musica Huasteca. *FRONTIERS IN PSYCHOLOGY* 2021; 12. DOI: 10.3389/fpsyg.2021.648010.

49. Li J and Brar A. The use and impact of digital technologies for and on the mental health and wellbeing of Indigenous people: A systematic review of empirical studies. *COMPUTERS IN HUMAN BEHAVIOR* 2022; 126. DOI: 10.1016/j.chb.2021.106988.

50. Leong N, Laughter L and Rowe DJ. Challenges of Dental Assisting Students in Their Pursuit of Academic Success. *JOURNAL OF DENTAL EDUCATION* 2017; 81: 433-441. DOI: 10.21815/JDE.016.007.

51. Kyoon-Achan G, Schroth RJ, Sanguins J, et al. Early childhood oral health promotion for First Nations and Metis communities and caregivers in Manitoba. *HEALTH PROMOTION AND CHRONIC DISEASE PREVENTION IN CANADA-RESEARCH POLICY AND PRACTICE* 2021; 41: 14-24. DOI: 10.24095/hpcdp.41.1.02.

52. Kohlhagen J, Massey PD, Taylor KA, et al. Describing meningococcal disease: understanding, perceptions and feelings of people in a regional area of NSW, Australia. *PUBLIC HEALTH RESEARCH & PRACTICE* 2016; 26. DOI: 10.17061/phrp2651658.

53. Koch T, Mann S, Kralik D, et al. Reflection Look, think and act cycles in participatory action research. *JOURNAL OF RESEARCH IN NURSING* 2005; 10: 261-278. DOI: 10.1177/174498710501000304.

54. Kirkham R, MacKay D, Barzi F, et al. Improving postpartum screening after diabetes in pregnancy: Results of a pilot study in remote Australia. *AUSTRALIAN & NEW ZEALAND JOURNAL OF OBSTETRICS & GYNAECOLOGY* 2019; 59: 430-435. DOI: 10.1111/ajo.12894.

55. Kira A, Glover M, Walker N, et al. Recruiting Pregnant Indigenous Women Who Smoke into a High Contact Incentivized Cessation Trial: A Feasibility Study. *NICOTINE & TOBACCO RESEARCH* 2016; 18: 2036-2040. DOI: 10.1093/ntr/ntw106.

56. Kidd S, Davidson L, Frederick T, et al. Reflecting on Participatory, Action-Oriented Research Methods in Community Psychology: Progress, Problems, and Paths Forward. *AMERICAN JOURNAL OF COMMUNITY PSYCHOLOGY* 2018; 61: 76-87. DOI: 10.1002/ajcp.12214.

57. Kerrigan V, Herdman RM, Thomas DP, et al. 'I still remember your post about buying smokes': a case study of a remote Aboriginal community-controlled health service using Facebook for tobacco control. *AUSTRALIAN JOURNAL OF PRIMARY HEALTH* 2019; 25: 443-448. DOI: 10.1071/PY19008.

58. Kerr B, Stephens D, Pham D, et al. Assessing the Usability, Appeal, and Impact of a Web-Based Training for Adults Responding to Concerning Posts on Social Media: Pilot Suicide Prevention Study. *JMIR MENTAL HEALTH* 2020; 7. DOI: 10.2196/14949.

59. Kennedy M, Kumar R, Ryan NM, et al. Codeveloping a multibehavioural mobile phone app to enhance social and emotional well-being and reduce health risks among Aboriginal and Torres Strait Islander women during preconception and pregnancy: a three-phased mixed-methods study. *BMJ OPEN* 2021; 11. DOI: 10.1136/bmjopen-2021-052545.

60. Kamis K, Janevic MR, Marinec N, et al. A study of mobile phone use among patients with noncommunicable diseases in La Paz, Bolivia: implications for mHealth research and development. *GLOBALIZATION AND HEALTH* 2015; 11. DOI: 10.1186/s12992-015-0115-y.

61. Jeffs E, Williman J, Brunton C, et al. Pregnant women's knowledge of, and adherence to, New Zealand Food Safety in Pregnancy guidelines. *NEW ZEALAND MEDICAL JOURNAL* 2020; 133: 41-52.

62. Janca A, Lyons Z and Gaspar J. Here and Now Aboriginal Assessment (HANAA): a follow-up survey of users. *AUSTRALASIAN PSYCHIATRY* 2017; 25: 288-289. DOI: 10.1177/1039856217700806.

63. Immanuel J, Eagleton C, Baker J, et al. Pregnancy outcomes among multi-ethnic women with different degrees of hyperglycaemia during pregnancy in an urban New Zealand population and their association with postnatal HbA1c uptake. *AUSTRALIAN & NEW ZEALAND JOURNAL OF OBSTETRICS & GYNAECOLOGY* 2021; 61: 69-77. DOI: 10.1111/ajo.13231.

64. Hutt-MacLeod D, Rudderham H, Sylliboy A, et al. Eskasoni First Nation's transformation of youth mental healthcare: Partnership between a Mi'kmaq community and the ACCESS Open Minds research project in implementing innovative practice and service evaluation. *EARLY INTERVENTION IN PSYCHIATRY* 2019; 13: 42-47. DOI: 10.1111/eip.12817.

65. Hume A, Wetten A, Feeney C, et al. Remote school gardens: exploring a cost-effective and novel way to engage Australian Indigenous students in nutrition and health. *AUSTRALIAN AND NEW ZEALAND JOURNAL OF PUBLIC HEALTH* 2014; 38: 235-240. DOI: 10.1111/1753-6405.12236.

66. Hui A, Philips-Beck W, Campbell R, et al. Impact of remote prenatal education on program participation and breastfeeding of women in rural and remote Indigenous communities. *ECLINICALMEDICINE* 2021; 35. DOI: 10.1016/j.eclinm.2021.100851.

67. Hswen Y, Naslund JA and Bickham DS. Differences in media access and use between rural Native American and White children. *RURAL AND REMOTE HEALTH* 2014; 14.

68. Helitzer DL, Hathorn G, Benally J, et al. Culturally Relevant Model Program to Prevent and Reduce Agricultural Injuries. *JOURNAL OF AGRICULTURAL SAFETY AND HEALTH* 2014; 20: 175-198. DOI: 10.13031/jash.20.10333.

69. Hefler M, Kerrigan V, Henryks J, et al. Social media and health information sharing among Australian Indigenous people. *HEALTH PROMOTION INTERNATIONAL* 2019; 34: 706-715. DOI: 10.1093/heapro/day018.

70. Hefler M, Kerrigan V, Grunseit A, et al. Facebook-Based Social Marketing to Reduce Smoking in Australia's First Nations Communities: An Analysis of Reach, Shares, and Likes. *JOURNAL OF MEDICAL INTERNET RESEARCH* 2020; 22. DOI: 10.2196/16927.

71. Hefler M, Kerrigan V, Freeman B, et al. Using Facebook to reduce smoking among Australian Aboriginal and Torres Strait Islander people: a participatory grounded action study. *BMC PUBLIC HEALTH* 2019; 19. DOI: 10.1186/s12889-019-6918-7.

72. Heaney E, Hunter L, Clulow A, et al. Efficacy of Communication Techniques and Health Outcomes of Bushfire Smoke Exposure: A Scoping Review. *INTERNATIONAL JOURNAL OF ENVIRONMENTAL RESEARCH AND PUBLIC HEALTH* 2021; 18. DOI: 10.3390/ijerph182010889.

73. Hausmann JS, Kennedy K, Simard JF, et al. Immediate effect of the COVID-19 pandemic on patient health, health-care use, and behaviours: results from an international survey of people with rheumatic diseases. *LANCET RHEUMATOLOGY* 2021; 3: E707-E714. DOI: 10.1016/S2665-9913(21)00175-2.

74. Harris R, Van Dyke ER, Ton TGN, et al. Assessing Needs for Cancer Education and Support in American Indian and Alaska Native Communities in the Northwestern United States. *HEALTH PROMOTION PRACTICE* 2016; 17: 891-898. DOI: 10.1177/1524839915611869.

75. Hanrahan SJ and Schinke R. *USING PSYCHOLOGICAL SKILLS TRAINING FROM SPORT PSYCHOLOGY TO ENHANCE THE LIFE SATISFACTION OF ADOLESCENT MEXICAN ORPHANS*. 2009, p.171-180.

76. Hamilton SJ, Mills B, Birch EM, et al. Smartphones in the secondary prevention of cardiovascular disease: a systematic review. *BMC CARDIOVASCULAR DISORDERS* 2018; 18. DOI: 10.1186/s12872-018-0764-x.

77. Hale L, Devan H, Davies C, et al. Clinical and cost-effectiveness of an online-delivered group-based pain management programme in improving pain-related disability for people with persistent pain-protocol for a non-inferiority randomised controlled trial (iSelf-help trial). *BMJ OPEN* 2021; 11. DOI: 10.1136/bmjopen-2020-046376.

78. Gwynne K, Flaskas Y, O'Brien C, et al. Opportunistic screening to detect atrial fibrillation in Aboriginal adults in Australia. *BMJ OPEN* 2016; 6. DOI: 10.1136/bmjopen-2016-013576.

79. Gwynn J, Gwynne K, Rodrigues R, et al. Atrial Fibrillation in Indigenous Australians: A Multisite Screening Study Using a Single-Lead ECG Device in Aboriginal Primary Health Settings. *HEART LUNG AND CIRCULATION* 2021; 30: 267-274. DOI: 10.1016/j.hlc.2020.06.009.

80. Gunasekaran B, Jayasinghe Y, Fenner Y, et al. Knowledge of human papillomavirus and cervical cancer among young women recruited using a social networking site. *SEXUALLY TRANSMITTED INFECTIONS* 2013; 89: 327-329. DOI: 10.1136/sextrans-2012-050612.

81. Gunasekaran B, Jayasinghe Y, Brotherton JML, et al. Asking about human papillomavirus vaccination and the usefulness of registry validation: A study of young women recruited using Facebook. *VACCINE* 2015; 33: 826-831. DOI: 10.1016/j.vaccine.2014.11.002.

82. Grout L, Telfer K, Wilson N, et al. Prescribing Smartphone Apps for Physical Activity Promotion in Primary Care: Modeling Study of Health Gain and Cost Savings. *JOURNAL OF MEDICAL INTERNET RESEARCH* 2021; 23. DOI: 10.2196/31702.

83. Graham AL and Papandonatos GD. Reliability of Internet- versus telephone-administered questionnaires in a diverse sample of smokers. *JOURNAL OF MEDICAL INTERNET RESEARCH* 2008; 10. DOI: 10.2196/jmir.987.

84. Gould GS, Holder C, Oldmeadow C, et al. Supports Used by Aboriginal and Torres Strait Islander Women for Their Health, including Smoking Cessation, and a Baby's Health: A Cross-Sectional Survey in New South Wales, Australia. *INTERNATIONAL JOURNAL OF ENVIRONMENTAL RESEARCH AND PUBLIC HEALTH* 2020; 17. DOI: 10.3390/ijerph17217766.

85. Gould CC. Solidarity and the problem of structural injustice in healthcare. *BIOETHICS* 2018; 32: 541-552. DOI: 10.1111/bioe.12474.

86. Gittelsohn J, Jock B, Redmond L, et al. OPREVENT2: Design of a multi-institutional intervention for obesity control and prevention for American Indian adults. *BMC PUBLIC HEALTH* 2017; 17. DOI: 10.1186/s12889-017-4018-0.

87. Geia L, Pearson L and Sweet M. Narratives of Twitter as a Platform for Professional Development, Innovation, and Advocacy. *AUSTRALIAN PSYCHOLOGIST* 2017; 52: 280-287. DOI: 10.1111/ap.12279.

88. Gaugler JE, McCarron HR and Mitchell LL. Perceptions of precision medicine among diverse dementia caregivers and professional providers. *ALZHEIMERS & DEMENTIA-TRANSLATIONAL RESEARCH & CLINICAL INTERVENTIONS* 2019; 5: 468-474. DOI: 10.1016/j.trci.2019.07.005.

89. Gantz L, Calvo A, Hess-Holtz M, et al. Predictors of HPV Knowledge and HPV Vaccine Awareness Among Women in Panama City, Panama. *WORLD MEDICAL & HEALTH POLICY* 2019; 11: 95-118. DOI: 10.1002/wmh3.293.

90. Foulds HJA, Rodgers CD, Duncan V, et al. A systematic review and meta-analysis of screen time behaviour among North American indigenous populations. *OBESITY REVIEWS* 2016; 17: 455-466. DOI: 10.1111/obr.12389.

91. Forbes D and Wongthongtham P. Ontology based intercultural patient practitioner assistive communications from qualitative gap analysis. *INFORMATION TECHNOLOGY & PEOPLE* 2016; 29: 280-317. DOI: 10.1108/ITP-08-2014-0166.

92. Flemington T, La Hera-Fuentes G, Bovill M, et al. Smoking Cessation Messages for Pregnant Aboriginal and Torres Strait Islander Women: A Rapid Review of Peer-Reviewed Literature and Assessment of Research Translation of Media Content. *INTERNATIONAL JOURNAL OF ENVIRONMENTAL RESEARCH AND PUBLIC HEALTH* 2021; 18. DOI: 10.3390/ijerph18179341.

93. Filippi MK, McCloskey C, Williams C, et al. Perceptions, Barriers, and Suggestions for Creation of a Tobacco and Health Website Among American Indian/Alaska Native College Students. *JOURNAL OF COMMUNITY HEALTH* 2013; 38: 486-491. DOI: 10.1007/s10900-012-9634-0.

94. Davies J, Bukulatjpi S, Sharma S, et al. Development of a Culturally Appropriate Bilingual Electronic App About Hepatitis B for Indigenous Australians: Towards Shared Understandings. *JMIR RESEARCH PROTOCOLS* 2015; 4. DOI: 10.2196/resprot.4216.

95. Daley CM, James AS, Ulrey E, et al. Using Focus Groups in Community-Based Participatory Research: Challenges and Resolutions. *QUALITATIVE HEALTH RESEARCH* 2010; 20: 697-706. DOI: 10.1177/1049732310361468.

96. Cleghorn C, Wilson N, Nair N, et al. Health Benefits and Cost-Effectiveness From Promoting Smartphone Apps for Weight Loss: Multistate Life Table Modeling. *JMIR MHEALTH AND UHEALTH* 2019; 7. DOI: 10.2196/11118.

97. Christie V, Rice M, Dracakis J, et al. Improving breast cancer outcomes for Aboriginal women: a mixed-methods study protocol. *BMJ OPEN* 2022; 12. DOI: 10.1136/bmjopen-2020-048003.

98. Choukou MA, Sanchez-Ramirez DC, Pol M, et al. COVID-19 infodemic and digital health literacy in vulnerable populations: A scoping review. *DIGITAL HEALTH* 2022; 8. DOI: 10.1177/20552076221076927.

99. Chellew N, Chang AB and Grimwood K. Azithromycin Prescribing by Respiratory Pediatricians in Australia and New Zealand for Chronic Wet Cough: A Questionnaire-Based Survey. *FRONTIERS IN PEDIATRICS* 2020; 8. DOI: 10.3389/fped.2020.00519.

100. Chamberlain C, Perlen S, Brennan S, et al. Evidence for a comprehensive approach to Aboriginal tobacco control to maintain the decline in smoking: an overview of reviews among Indigenous peoples. *SYSTEMATIC REVIEWS* 2017; 6. DOI: 10.1186/s13643-017-0520-9.

101. Carson TL, Cardel MI, Stanley TL, et al. Racial and ethnic representation among a sample of nutrition- and obesity-focused professional organizations in the United States. *AMERICAN JOURNAL OF CLINICAL NUTRITION* 2021; 114: 1869-1872. DOI: 10.1093/ajcn/nqab284.

102. Carlson B, Jones LV, Harris M, et al. Trauma, Shared Recognition and Indigenous Resistance on Social Media. *AUSTRALASIAN JOURNAL OF INFORMATION SYSTEMS* 2017; 21.

103. Carlson B, Frazer R and Farrelly T. "That makes all the difference": Aboriginal and Torres Strait Islander health-seeking on social media. *HEALTH PROMOTION JOURNAL OF AUSTRALIA* 2021; 32: 523-531. DOI: 10.1002/hpja.366.

104. Brusse C, Gardner K, McAullay D, et al. Social Media and Mobile Apps for Health Promotion in Australian Indigenous Populations: Scoping Review. *JOURNAL OF MEDICAL INTERNET RESEARCH* 2014; 16: 56-70. DOI: 10.2196/jmir.3614.

105. Britt RK, Britt BC, Anderson J, et al. "Sharing Hope and Healing": A Culturally Tailored Social Media Campaign to Promote Living Kidney Donation and Transplantation Among Native Americans. *HEALTH PROMOTION PRACTICE* 2021; 22: 786-795. DOI: 10.1177/1524839920974580.

106. Bovill M, Chamberlain C, Bennett J, et al. Building an Indigenous-Led Evidence Base for Smoking Cessation Care among Aboriginal and Torres Strait Islander Women during Pregnancy and Beyond: Research Protocol for the Which Way? Project. *INTERNATIONAL JOURNAL OF ENVIRONMENTAL RESEARCH AND PUBLIC HEALTH* 2021; 18. DOI: 10.3390/ijerph18031342.

107. Boutrin MC and Williams DR. What Racism Has to Do with It: Understanding and Reducing Sexually Transmitted Diseases in Youth of Color. *HEALTHCARE* 2021; 9. DOI: 10.3390/healthcare9060673.

108. Bonner A, Gillespie K, Campbell KL, et al. Evaluating the prevalence and opportunity for technology use in chronic kidney disease patients: a cross-sectional study. *BMC NEPHROLOGY* 2018; 19. DOI: 10.1186/s12882-018-0830-8.

109. Blinkhorn F, Wallace J, Smith L, et al. Developing leaflets to give dental health advice to Aboriginal families with young children. *INTERNATIONAL DENTAL JOURNAL* 2014; 64: 195-199. DOI: 10.1111/idj.12108.

110. Berends L and Halliday R. Capacity building and social marketing promotes healthy lifestyle behaviour in an Australian Aboriginal community. *AUSTRALIAN JOURNAL OF RURAL HEALTH* 2018; 26: 279-283. DOI: 10.1111/ajr.12421.

111. Bennett-Levy J, Singer J, DuBois S, et al. Translating E-Mental Health Into Practice: What Are the Barriers and Enablers to E-Mental Health Implementation by Aboriginal and Torres Strait Islander Health Professionals? *JOURNAL OF MEDICAL INTERNET RESEARCH* 2017; 19. DOI: 10.2196/jmir.6269.

112. Ashman AM, Collins CE, Brown LJ, et al. A Brief Tool to Assess Image-Based Dietary Records and Guide Nutrition Counselling Among Pregnant Women: An Evaluation. *JMIR MHEALTH AND UHEALTH* 2016; 4. DOI: 10.2196/mhealth.6469.

113. Anderson K, Gall A, Butler T, et al. Using web conferencing to engage Aboriginal and Torres Strait Islander young people in research: a feasibility study. *BMC MEDICAL RESEARCH METHODOLOGY* 2021; 21. DOI: 10.1186/s12874-021-01366-y.

114. Anderson G, Gleeson S, Rissel C, et al. Twitter tweets and twaddle: twittering at AHPA's National Health Promotion Conference. *HEALTH PROMOTION JOURNAL OF AUSTRALIA* 2014; 25: 143-146. DOI: 10.1071/HE13112.

115. Ali-Hassan H, Eloulabi R and Keethakumar A. Internet non-use among Canadian indigenous older adults: Aboriginal Peoples Survey (APS). *BMC PUBLIC HEALTH* 2020; 20. DOI: 10.1186/s12889-020-09659-5.

116. Alexander J, Kwon HT, Strecher R, et al. Multicultural Media Outreach: Increasing Cancer Information Coverage in Minority Communities. *JOURNAL OF CANCER EDUCATION* 2013; 28: 744-747. DOI: 10.1007/s13187-013-0534-5.

# SCOPUS. 2022.03.09

## Notes about this database search

Mesh terms do not transfer to this database – results in over a million hits

Went back to original plain English to start and modified as I went until I was getting a reasonable number of hits that were in the ballpark of correct. Important on this database to limit by the subject terms, topics areas and disciplines (everything on the left column needs to be reviewed)

## Actual search terms for SCOPUS

( TITLE-ABS-KEY ( smartphone OR {Mobile application} OR {mobile app} OR {Social Media} OR facebook OR snapchat OR instagram OR twitter OR tictoc OR pinterest OR linkedin OR youtube OR blogging OR email OR {social networking} OR {internet use} OR {internet application} OR email OR {Digital Interventions} ) AND TITLE-ABS-KEY ( aboriginal OR "Indigenous people" OR {American Native Continental ancestry} OR {Oceanic Ancestry Group} OR {First Nations} OR {Torres Strait Islander} OR {Native American} OR maori OR sami OR inuit OR ainu OR {American Indian} OR {Alaskan Natives} ) AND TITLE-ABS-KEY ( {Consumer health information} OR {health literacy} OR {health promotion} OR {health education} OR {chronic disease} OR {chronic illness} OR disease OR cancer OR neoplasms OR {cardiovascular disease} OR diabetes OR {Healthy people programs} OR {weight reduction} OR {sex education} OR {smoking prevention} OR {smoking cessation} OR {smoking reduction} OR {tobacco use} OR smoking OR vaping OR selfcare OR self-care OR {Quality of Life} OR stress OR wellbeing OR coping OR alcoholism OR addiction ) ) AND PUBYEAR > 2004 AND ( EXCLUDE ( SUBJAREA , "SOCI" ) OR EXCLUDE ( SUBJAREA , "ENVI" ) OR EXCLUDE ( SUBJAREA , "COMP" ) OR EXCLUDE ( SUBJAREA , "BIOC" ) OR EXCLUDE ( SUBJAREA , "AGRI" ) OR EXCLUDE ( SUBJAREA , "ARTS" ) OR EXCLUDE ( SUBJAREA , "PHAR" ) OR EXCLUDE ( SUBJAREA , "ENGI" ) OR EXCLUDE ( SUBJAREA , "NEUR" ) OR EXCLUDE ( SUBJAREA , "MULT" ) OR EXCLUDE ( SUBJAREA , "CENG" ) OR EXCLUDE ( SUBJAREA , "DECI" ) OR EXCLUDE ( SUBJAREA , "DENT" ) OR EXCLUDE ( SUBJAREA , "ECON" ) OR EXCLUDE ( SUBJAREA , "IMMU" ) OR EXCLUDE ( SUBJAREA , "MATH" ) ) AND ( LIMIT-TO ( LANGUAGE , "English" ) =135

## Link to saved search for SCOPUS

https://www-scopus-com.simsrad.net.ocs.mq.edu.au/search/save/savedSearch.uri?origin=userDashboard&zone=TopNavBar

## References found by SCOPUS

1. Yi JC, Sullivan B, Leisenring WM, et al. Who Enrolls in an Online Cancer Survivorship Program? Reach of the INSPIRE Randomized Controlled Trial for Hematopoietic Cell Transplantation Survivors. *Biology of Blood and Marrow Transplantation* 2020; 26: 1948-1954. Article. DOI: 10.1016/j.bbmt.2020.06.017.

2. Wood S and Schulman K. When Vaccine Apathy, Not Hesitancy, Drives Vaccine Disinterest. *JAMA - Journal of the American Medical Association* 2021; 325: 2435-2436. Note. DOI: 10.1001/jama.2021.7707.

3. Winch S, Ahmed N, Rissel C, et al. The reach and flow of health information in two Aboriginal communities: A social network analysis. *Australian Journal of Primary Health* 2017; 23: 189-195. Article. DOI: 10.1071/PY16024.

4. Webkamigad S, Rowe R, Peltier S, et al. Identifying and understanding the health and social care needs of Indigenous older adults with multiple chronic conditions and their caregivers: A scoping review. *BMC Geriatrics* 2020; 20. Article. DOI: 10.1186/s12877-020-01552-5.

5. Wan N, Wen M, Fan JX, et al. Physical activity barriers and facilitators among us pacific islanders and the feasibility of using mobile technologies for intervention: A focus group study with tongan Americans. *Journal of Physical Activity and Health* 2018; 15: 287-294. Article. DOI: 10.1123/jpah.2017-0014.

6. Walker T, Molenaar A and Palermo C. A qualitative study exploring what it means to be healthy for young Indigenous Australians and the role of social media in influencing health behaviour. *Health Promotion Journal of Australia* 2021; 32: 532-540. Article. DOI: 10.1002/hpja.391.

7. Volkova E, Michie J, Corrigan C, et al. Effectiveness of recruitment to a smartphone-delivered nutrition intervention in New Zealand: Analysis of a randomised controlled trial. *BMJ Open* 2017; 7. Article. DOI: 10.1136/bmjopen-2017-016198.

8. Vave R. Urban-Rural Compliance Variability to COVID-19 Restrictions of Indigenous Fijian (iTaukei) Funerals in Fiji. *Asia-Pacific Journal of Public Health* 2021; 33: 767-774. Article. DOI: 10.1177/10105395211005921.

9. Trofholz A, Tate A, Janowiec M, et al. Ecological Momentary Assessment of Weight-Related Behaviors in the Home Environment of Children from Low-Income and Racially and Ethnically Diverse Households: Development and Usability Study. *JMIR Research Protocols* 2021; 10. Review. DOI: 10.2196/30525.

10. Towns S, DiFranza JR, Jayasuriya G, et al. Smoking Cessation in Adolescents: targeted approaches that work. *Paediatric Respiratory Reviews* 2017; 22: 11-22. Review. DOI: 10.1016/j.prrv.2015.06.001.

11. Tighe J, Shand F, Ridani R, et al. Ibobbly mobile health intervention for suicide prevention in Australian Indigenous youth: A pilot randomised controlled trial. *BMJ Open* 2017; 7. Article. DOI: 10.1136/bmjopen-2016-013518.

12. Tighe J, Shand F, McKay K, et al. Usage and acceptability of the iBobbly app: Pilot trial for suicide prevention in aboriginal and torres strait islander youth. *JMIR Mental Health* 2020; 7. Article. DOI: 10.2196/14296.

13. Sweet MA. Social media: New links for indigenous health. *Medical Journal of Australia* 2013; 199: 18. Note. DOI: 10.5694/mja13.10429.

14. Sweet M, Geia L, Dudgeon P, et al. #IHMayDay: tweeting for empowerment and social and emotional wellbeing. *Australasian psychiatry : bulletin of Royal Australian and New Zealand College of Psychiatrists* 2015; 23: 636-640. Article. DOI: 10.1177/1039856215609762.

15. Swannell C. COVID-19, Black Lives Matter and making a difference. *Medical Journal of Australia* 2020; 213: C1. Note. DOI: 10.5694/mja2.50723.

16. Strudwick G, Sockalingam S, Kassam I, et al. Digital interventions to support population mental health in canada during the covid-19 pandemic: Rapid review. *JMIR Mental Health* 2021; 8. Review. DOI: 10.2196/26550.

17. Stotz S, Brega AG, Lockhart S, et al. An online diabetes nutrition education programme for American Indian and Alaska Native adults with type 2 diabetes: Perspectives from key stakeholders. *Public Health Nutrition* 2021; 24: 1449-1459. Review. DOI: 10.1017/S1368980020001743.

18. Stockman JK, Wood BA and Anderson KM. Racial and ethnic differences in COVID-19 outcomes, stressors, fear, and prevention behaviors among US women: Web-based cross-sectional study. *Journal of Medical Internet Research* 2021; 23. Article. DOI: 10.2196/26296.

19. Snyder T, Ravenhurst J, Cramer EY, et al. Serological surveys to estimate cumulative incidence of SARS-CoV-2 infection in adults (Sero-MAss study), Massachusetts, July-August 2020: A mail-based cross-sectional study. *BMJ Open* 2021; 11. Article. DOI: 10.1136/bmjopen-2021-051157.

20. Snijder M, Lees B, Ward J, et al. Developing an ecological framework of factors associated with substance use and related harms among Aboriginal and Torres Strait Islander people: Protocol for a systematic review. *BMJ Open* 2019; 9. Review. DOI: 10.1136/bmjopen-2018-024418.

21. Smith JA, Merlino A, Christie B, et al. Using social media in health literacy research: A promising example involving Facebook with young Aboriginal and Torres Strait Islander males from the Top End of the Northern Territory. *Health Promotion Journal of Australia* 2021; 32: 186-191. Article. DOI: 10.1002/hpja.421.

22. Smartlowit-Briggs L, Pearson C, Whitefoot P, et al. Community-Based Assessment to Inform a Chlamydia Screening Program for Women in a Rural American Indian Community. *Sexually Transmitted Diseases* 2016; 43: 390-395. Article. DOI: 10.1097/OLQ.0000000000000456.

23. Slater JJ and Mudryj AN. Are we really "eating well with Canada's food guide"? *BMC Public Health* 2018; 18. Article. DOI: 10.1186/s12889-018-5540-4.

24. Sinka V, Lopez-Vargas P, Tong A, et al. Chronic disease prevention programs offered by Aboriginal Community Controlled Health Services in New South Wales, Australia. *Australian and New Zealand Journal of Public Health* 2021; 45: 59-64. Article. DOI: 10.1111/1753-6405.13069.

25. Sinicrope PS, Young CD, Resnicow K, et al. Lessons Learned From Beta-Testing a Facebook Group Prototype to Promote Treatment Use in the “Connecting Alaska Native People to Quit Smoking” (CAN Quit) Study. *Journal of Medical Internet Research* 2022; 24. Article. DOI: 10.2196/28704.

26. Shibasaki S, Gardner K and Sibthorpe B. Using knowledge translation to craft “sticky” social media health messages that provoke interest, raise awareness, impart knowledge, and inspire change. *JMIR mHealth and uHealth* 2016; 4. Article. DOI: 10.2196/mhealth.5987.

27. Shand F, MacKinnon A, O'Moore K, et al. The iBobbly Aboriginal and Torres Strait Islander app project: Study protocol for a randomised controlled trial. *Trials* 2019; 20. Article. DOI: 10.1186/s13063-019-3262-2.

28. Shaeer O and Shaeer K. The Global Online Sexuality Survey (GOSS): The United States of America in 2011. Chapter I: Erectile Dysfunction Among English-Speakers. *Journal of Sexual Medicine* 2012; 9: 3018-3027. Article. DOI: 10.1111/j.1743-6109.2012.02976.x.

29. Serlachius A, Schache K, Boggiss A, et al. Coping skills mobile app to support the emotional well-being of young people during the COVID-19 pandemic: Protocol for a mixed methods study. *JMIR Research Protocols* 2020; 9. Article. DOI: 10.2196/23716.

30. Serlachius A, Boggiss A, Lim D, et al. Pilot study of a well-being app to support New Zealand young people during the COVID-19 pandemic. *Internet Interventions* 2021; 26. Article. DOI: 10.1016/j.invent.2021.100464.

31. Scarton LJ, Bakas T, Miller WR, et al. Development and Psychometric Testing of the Diabetes Caregiver Activity and Support Scale. *Diabetes Educator* 2017; 43: 465-475. Article. DOI: 10.1177/0145721717726280.

32. Saxton PJW, Azariah S, Franklin RA, et al. Baseline characteristics of gay and bisexual men in a HIV pre-exposure prophylaxis demonstration project with equity quotas in Auckland, New Zealand. *Sexual Health* 2019; 16: 47-55. Article. DOI: 10.1071/SH18056.

33. Sarkar U, Le GM, Lyles CR, et al. Using social media to target cancer prevention in young adults: Viewpoint. *Journal of Medical Internet Research* 2018; 20. Article. DOI: 10.2196/jmir.8882.

34. Santiago-Torres M, Mull KE, Sullivan BM, et al. Efficacy and Utilization of Smartphone Applications for Smoking Cessation Among American Indians and Alaska Natives: Results From the iCanQuit Trial. *Nicotine & tobacco research : official journal of the Society for Research on Nicotine and Tobacco* 2022; 24: 544-554. Article. DOI: 10.1093/ntr/ntab213.

35. Rychert M, Wilkins C, Parker K, et al. Exploring medicinal use of cannabis in a time of policy change in New Zealand. *The New Zealand medical journal* 2020; 133: 54-69. Article.

36. Rushing SC, Kelley A, Bull S, et al. Efficacy of an mhealth intervention (brave) to promote mental wellness for american indian and alaska native teenagers and young adults: Randomized controlled trial. *JMIR Mental Health* 2021; 8. Article. DOI: 10.2196/26158.

37. Rose SB, Garrett SM, McKinlay EM, et al. Access to sexual healthcare during New Zealand's COVID-19 lockdown: Cross-sectional online survey of 15-24-year-olds in a high deprivation region. *BMJ Sexual and Reproductive Health* 2021; 47: 277-284. Article. DOI: 10.1136/bmjsrh-2020-200986.

38. Robards F, Kang M, Steinbeck K, et al. Health care equity and access for marginalised young people: A longitudinal qualitative study exploring health system navigation in Australia. *International Journal for Equity in Health* 2019; 18. Article. DOI: 10.1186/s12939-019-0941-2.

39. Rice ES, Haynes E, Royce P, et al. Social media and digital technology use among Indigenous young people in Australia: A literature review. *International Journal for Equity in Health* 2016; 15. Review. DOI: 10.1186/s12939-016-0366-0.

40. Reti SR, Feldman HJ and Safran C. Online access and literacy in Maori New Zealanders with diabetes. *Journal of Primary Health Care* 2011; 3: 190-191. Article. DOI: 10.1071/hc11190.

41. Redfern J, Usherwood T, Harris MF, et al. A randomised controlled trial of a consumer-focused e-health strategy for cardiovascular risk management in primary care: The Consumer Navigation of Electronic Cardiovascular Tools (CONNECT) study protocol. *BMJ Open* 2014; 4. Article. DOI: 10.1136/bmjopen-2013-004523.

42. Ragupathy R, Yogarajan V and Luoni C. Health information research privacy standards should include Māori perspectives on privacy. *New Zealand Medical Journal* 2019; 132: 64-67. Letter.

43. Povey J, Mills PPJR, Dingwall KM, et al. Acceptability of mental health apps for aboriginal and torres strait islander australians: A qualitative study. *Journal of Medical Internet Research* 2016; 18. Article. DOI: 10.2196/jmir.5314.

44. Peterson J, Erickson G, Knapp K, et al. Communication of Intent to Do Harm Preceding Mass Public Shootings in the United States, 1966 to 2019. *JAMA Network Open* 2021; 4. Article. DOI: 10.1001/jamanetworkopen.2021.33073.

45. Peiris D, Wright L, News M, et al. A smartphone app to assist smoking cessation among aboriginal Australians: Findings from a pilot randomized controlled trial. *JMIR mHealth and uHealth* 2019; 7. Article. DOI: 10.2196/12745.

46. Papautsky EL, Rice DR, Ghoneima H, et al. Characterizing health care delays and interruptions in the United States during the COVID-19 pandemic: Internet-based, cross-sectional survey study. *Journal of Medical Internet Research* 2021; 23. Review. DOI: 10.2196/25446.

47. Okorodudu DO and Okorodudu DE. An issue of trust—vaccinating Black patients against COVID-19. *The Lancet Respiratory Medicine* 2021; 9: 228-229. Note. DOI: 10.1016/S2213-2600(21)00002-3.

48. Nugus P, Désalliers J, Morales J, et al. Localizing Global Medicine: Challenges and Opportunities in Cervical Screening in an Indigenous Community in Ecuador. *Qualitative Health Research* 2018; 28: 800-812. Article. DOI: 10.1177/1049732317742129.

49. Norris P, Cousins K, Churchward M, et al. Recruiting people facing social disadvantage: the experience of the Free Meds study. *International Journal for Equity in Health* 2021; 20. Article. DOI: 10.1186/s12939-021-01483-6.

50. Niznik JD, Harrison J, White EM, et al. Perceptions of COVID-19 vaccines among healthcare assistants: A national survey. *Journal of the American Geriatrics Society* 2022; 70: 8-18. Article. DOI: 10.1111/jgs.17437.

51. Nghiem N, Leung W, Cleghorn C, et al. Mass media promotion of a smartphone smoking cessation app: Modelled health and cost-saving impacts. *BMC Public Health* 2019; 19. Article. DOI: 10.1186/s12889-019-6605-8.

52. Nathan S, Rawstorne P, Hayen A, et al. Examining the pathways for young people with drug and alcohol dependence: A mixed-method design to examine the role of a treatment programme. *BMJ Open* 2016; 6. Article. DOI: 10.1136/bmjopen-2015-010824.

53. Mosby I and Swidrovich J. Medical experimentation and the roots of COVID-19 vaccine hesitancy among Indigenous Peoples in Canada. *CMAJ* 2021; 193: E381-E383. Note. DOI: 10.1503/cmaj.210112.

54. Mick P, Parfyonov M, Wittich W, et al. Associations between sensory loss and social networks, participation, support, and loneliness: Analysis of the Canadian Longitudinal Study on Aging. *Canadian Family Physician* 2018; 64: e33-e41. Article.

55. Metheny N and Stephenson R. Structural stressors and intimate partner violence in indigenous men who have sex with men in the United States. *Stigma and Health* 2020; 5: 492-496. Article. DOI: 10.1037/sah0000220.

56. Merculieff ZT, Koller KR, Sinicrope PS, et al. Developing a Social Media Intervention to Connect Alaska Native People Who Smoke with Resources and Support to Quit Smoking: The Connecting Alaska Native Quit Study. *Nicotine and Tobacco Research* 2021; 23: 1002-1009. Article. DOI: 10.1093/ntr/ntaa253.

57. Melvin GA, Gresham D, Beaton S, et al. Evaluating the Feasibility and Effectiveness of an Australian Safety Planning Smartphone Application: A Pilot Study Within a Tertiary Mental Health Service. *Suicide and Life-Threatening Behavior* 2019; 49: 846-858. Article. DOI: 10.1111/sltb.12490.

58. McGovern TF, Bundoc-Baronia R and Bergeson SE. Alcoholism Treatment Quarterly 2020, 38-4. *Alcoholism Treatment Quarterly* 2020; 38: 401-402. Editorial. DOI: 10.1080/07347324.2020.1816327.

59. McElfish PA, Rowland B, Riklon S, et al. Development and Evaluation of a Blood Glucose Monitoring YouTube Video for Marshallese Patients Using a Community-Based Participatory Research Approach. *Policy, Politics, and Nursing Practice* 2019; 20: 205-215. Article. DOI: 10.1177/1527154419872834.

60. McCrabb S, Twyman L, Palazzi K, et al. A cross sectional survey of internet use among a highly socially disadvantaged population of tobacco smokers. *Addiction Science and Clinical Practice* 2019; 14. Article. DOI: 10.1186/s13722-019-0168-y.

61. Mayer G, Alvarez S, Gronewold N, et al. Expressions of individualization on the internet and social media: multigenerational focus group study. *Journal of Medical Internet Research* 2020; 22. Article. DOI: 10.2196/20528.

62. Mattingly JA. Fostering Cultural Safety in Nursing Education: Experiential Learning on an American Indian Reservation. *Contemporary Nurse* 2022. Note. DOI: 10.1080/10376178.2021.2013124.

63. Mathieson K, Leafman JS and Horton MB. Access to digital communication technology and perceptions of telemedicine for patient education among American Indian patients with diabetes. *Journal of Health Care for the Poor and Underserved* 2017; 28: 1522-1536. Article. DOI: 10.1353/hpu.2017.0131.

64. Marson FAL and Ortega MM. COVID-19 in Brazil. *Pulmonology* 2020; 26: 241-244. Note. DOI: 10.1016/j.pulmoe.2020.04.008.

65. Makosky Daley C, James AS, Ulrey E, et al. Using focus groups in community-based participatory research: Challenges and resolutions. *Qualitative Health Research* 2010; 20: 697-706. Article. DOI: 10.1177/1049732310361468.

66. Macniven R, Gwynn J, Fujimoto H, et al. Feasibility and acceptability of opportunistic screening to detect atrial fibrillation in Aboriginal adults. *Australian and New Zealand Journal of Public Health* 2019; 43: 313-318. Article. DOI: 10.1111/1753-6405.12905.

67. Lombard C, Brennan L, Reid M, et al. Communicating health—Optimising young adults’ engagement with health messages using social media: Study protocol. *Nutrition and Dietetics* 2018; 75: 509-519. Article. DOI: 10.1111/1747-0080.12448.

68. Lee KSK, Conigrave JH, Callinan S, et al. Asking about the last four drinking occasions on a tablet computer as a way to record alcohol consumption in Aboriginal and Torres Strait Islander Australians: a validation. *Addiction science & clinical practice* 2019; 14: 15. Article. DOI: 10.1186/s13722-019-0148-2.

69. Lauckner C, Desrosiers A, Muilenburg J, et al. Social media photos of substance use and their relationship to attitudes and behaviors among ethnic and racial minority emerging adult men living in low-income areas. *Journal of Adolescence* 2019; 77: 152-162. Article. DOI: 10.1016/j.adolescence.2019.10.013.

70. Lane-Fall MB, Butler PD and Mahoney KB. Promoting Racial Justice and Equity in Academic Medicine: Moving beyond the Honeymoon Period. *JAMA - Journal of the American Medical Association* 2021; 326: 603-604. Note. DOI: 10.1001/jama.2021.9324.

71. Laffel LM, Kanapka LG, Beck RW, et al. Effect of Continuous Glucose Monitoring on Glycemic Control in Adolescents and Young Adults with Type 1 Diabetes: A Randomized Clinical Trial. *JAMA - Journal of the American Medical Association* 2020; 323: 2388-2396. Article. DOI: 10.1001/jama.2020.6940.

72. Kyoon-Achan G, Schroth RJ, Sanguins J, et al. Early childhood oral health promotion for first nations and métis communities and caregivers in Manitoba. *Health Promotion and Chronic Disease Prevention in Canada* 2021; 41: 14-24. Article. DOI: 10.24095/hpcdp.41.1.02.

73. Kohlhagen J, Massey PD, Taylor KA, et al. Describing meningococcal disease: Understanding, perceptions and feelings of people in a regional area of NSW, Australia. *Public Health Research and Practice* 2016; 26. Article. DOI: 10.17061/phrp2651658.

74. Koch T, Mann S, Kralik D, et al. Reflection: Look, think and act cycles in participatory action research. *Journal of Research in Nursing* 2005; 10: 261-278. Article. DOI: 10.1177/174498710501000304.

75. Kirkham R, MacKay D, Barzi F, et al. Improving postpartum screening after diabetes in pregnancy: Results of a pilot study in remote Australia. *Australian and New Zealand Journal of Obstetrics and Gynaecology* 2019; 59: 430-435. Article. DOI: 10.1111/ajo.12894.

76. Kira A, Glover M, Walker N, et al. Recruiting pregnant indigenous women who smoke into a high contact incentivized cessation trial: A feasibility study. *Nicotine and Tobacco Research* 2016; 18: 2036-2040. Article. DOI: 10.1093/ntr/ntw106.

77. Kerrigan V, Herdman RM, Thomas DP, et al. 'I still remember your post about buying smokes': A case study of a remote Aboriginal community-controlled health service using Facebook for tobacco control. *Australian Journal of Primary Health* 2019; 25: 443-448. Article. DOI: 10.1071/PY19008.

78. Kerr B, Stephens D, Pham D, et al. Assessing the usability, appeal, and impact of a web-based training for adults responding to concerning posts on social media: Pilot suicide prevention study. *JMIR Mental Health* 2020; 7. Article. DOI: 10.2196/14949.

79. Kelleher E and Moreno MA. Hot Topics in Social Media and Reproductive Health. *Journal of Pediatric and Adolescent Gynecology* 2020; 33: 619-622. Review. DOI: 10.1016/j.jpag.2020.06.016.

80. Kearns N, Shortt N, Kearns C, et al. How big is your bubble? Characteristics of self-isolating household units ('bubbles') during the COVID-19 Alert Level 4 period in New Zealand: A cross-sectional survey. *BMJ Open* 2021; 11. Article. DOI: 10.1136/bmjopen-2020-042464.

81. Kaufman CE, Black K, Keane EM, et al. Planning for a group-randomized trial with American Indian youth. *Journal of Adolescent Health* 2014; 54: S59-S63. Article. DOI: 10.1016/j.jadohealth.2013.12.028.

82. Kariippanon K and Senior K. Re-thinking knowledge landscapes in the context of grounded aboriginal theory and online health communication. *Croatian Medical Journal* 2018; 59: 33-38. Article. DOI: 10.3325/cmj.2018.59.33.

83. Kamdar N, Rozmus CL, Grimes DE, et al. Ethnic/Racial Comparisons in Strategies Parents Use to Cope with Food Insecurity: A Systematic Review of Published Research. *Journal of Immigrant and Minority Health* 2019; 21: 175-188. Article. DOI: 10.1007/s10903-018-0720-y.

84. Jeffs E, Williman J, Brunton C, et al. Pregnant women's knowledge of, and adherence to, New Zealand Food Safety in Pregnancy guidelines. *The New Zealand medical journal* 2020; 133: 41-52. Article.

85. Janca A, Lyons Z and Gaspar J. Here and now aboriginal assessment (HANAA): A follow-up survey of users. *Australasian Psychiatry* 2017; 25: 288-289. Article. DOI: 10.1177/1039856217700806.

86. Jackson MA, Brown AL, Baker AL, et al. The Incentives to Quit tobacco in Pregnancy (IQuiP) protocol: Piloting a financial incentive-based smoking treatment for women attending substance use in pregnancy antenatal services. *BMJ Open* 2019; 9. Article. DOI: 10.1136/bmjopen-2019-032330.

87. Immanuel J, Eagleton C, Baker J, et al. Pregnancy outcomes among multi-ethnic women with different degrees of hyperglycaemia during pregnancy in an urban New Zealand population and their association with postnatal HbA1c uptake. *Australian and New Zealand Journal of Obstetrics and Gynaecology* 2021; 61: 69-77. Article. DOI: 10.1111/ajo.13231.

88. Imlach F, McKinlay E, Middleton L, et al. Telehealth consultations in general practice during a pandemic lockdown: survey and interviews on patient experiences and preferences. *BMC Family Practice* 2020; 21. Article. DOI: 10.1186/s12875-020-01336-1.

89. Hume A, Wetten A, Feeney C, et al. Remote school gardens: Exploring a cost-effective and novel way to engage Australian Indigenous students in nutrition and health. *Australian and New Zealand Journal of Public Health* 2014; 38: 235-240. Article. DOI: 10.1111/1753-6405.12236.

90. Hui A, Philips-Beck W, Campbell R, et al. Impact of remote prenatal education on program participation and breastfeeding of women in rural and remote Indigenous communities. *EClinicalMedicine* 2021; 35. Article. DOI: 10.1016/j.eclinm.2021.100851.

91. Hochhauser S, Rao S, England-Kennedy E, et al. Why social justice matters: A context for suicide prevention efforts. *International Journal for Equity in Health* 2020; 19. Review. DOI: 10.1186/s12939-020-01173-9.

92. Hess KM, Crawford J, Eanes A, et al. Reasons Why Young Men Who Have Sex with Men Report Not Using HIV Pre-Exposure Prophylaxis: Perceptions of Burden, Need, and Safety. *AIDS Patient Care and STDs* 2019; 33: 449-454. Article. DOI: 10.1089/apc.2019.0150.

93. Hefler M, Kerrigan V, Grunseit A, et al. Facebook-based social marketing to reduce smoking in Australia's first nations communities: An analysis of reach, shares, and likes. *Journal of Medical Internet Research* 2020; 22. Article. DOI: 10.2196/16927.

94. Hefler M, Kerrigan V, Freeman B, et al. Using Facebook to reduce smoking among Australian Aboriginal and Torres Strait Islander people: A participatory grounded action study. *BMC Public Health* 2019; 19. Article. DOI: 10.1186/s12889-019-6918-7.

95. Harris R, Van Dyke ER, Ton TGN, et al. Assessing Needs for Cancer Education and Support in American Indian and Alaska Native Communities in the Northwestern United States. *Health Promotion Practice* 2016; 17: 891-898. Article. DOI: 10.1177/1524839915611869.

96. Harfield S, Elliott S, Ramsey L, et al. Using social networking sites to recruit participants: methods of an online survey of sexual health, knowledge and behaviour of young South Australians. *Australian and New Zealand Journal of Public Health* 2021; 45: 348-354. Article. DOI: 10.1111/1753-6405.13117.

97. Hamilton SJ, Mills B, Birch EM, et al. Smartphones in the secondary prevention of cardiovascular disease: A systematic review. *BMC Cardiovascular Disorders* 2018; 18. Article. DOI: 10.1186/s12872-018-0764-x.

98. Hale L, Devan H, Davies C, et al. Clinical and cost-effectiveness of an online-delivered group-based pain management programme in improving pain-related disability for people with persistent pain - Protocol for a non-inferiority randomised controlled trial (iSelf-help trial). *BMJ Open* 2021; 11. Article. DOI: 10.1136/bmjopen-2020-046376.

99. Gwynne K, Flaskas Y, O'Brien C, et al. Opportunistic screening to detect atrial fibrillation in Aboriginal adults in Australia. *BMJ Open* 2016; 6. Article. DOI: 10.1136/bmjopen-2016-013576.

100. Gwynn J, Gwynne K, Rodrigues R, et al. Atrial Fibrillation in Indigenous Australians: A Multisite Screening Study Using a Single-Lead ECG Device in Aboriginal Primary Health Settings. *Heart Lung and Circulation* 2021; 30: 267-274. Article. DOI: 10.1016/j.hlc.2020.06.009.

101. Grout L, Telfer K, Wilson N, et al. Prescribing Smartphone Apps for Physical Activity Promotion in Primary Care: Modeling Study of Health Gain and Cost Savings. *Journal of Medical Internet Research* 2021; 23. Article. DOI: 10.2196/31702.

102. Graham AL and Papandonatos GD. Reliability of internet- versus telephone-administered questionnaires in a diverse sample of smokers. *Journal of Medical Internet Research* 2008; 10. Article. DOI: 10.2196/jmir.987.

103. Gittelsohn J, Jock B, Redmond L, et al. OPREVENT2: Design of a multi-institutional intervention for obesity control and prevention for American Indian adults. *BMC Public Health* 2017; 17: 1-9. Article. DOI: 10.1186/s12889-017-4018-0.

104. Gaugler JE, McCarron HR and Mitchell LL. Perceptions of precision medicine among diverse dementia caregivers and professional providers. *Alzheimer's and Dementia: Translational Research and Clinical Interventions* 2019; 5: 468-474. Article. DOI: 10.1016/j.trci.2019.07.005.

105. Garcia P, Montez-Rath ME, Moore H, et al. SARS-CoV-2 vaccine acceptability in patients on hemodialysis: A nationwide survey. *Journal of the American Society of Nephrology* 2021; 32: 1575-1581. Article. DOI: 10.1681/ASN.2021010104.

106. Fuller J, Hermeston W, Passey M, et al. Acceptability of participatory social network analysis for problem-solving in Australian Aboriginal health service partnerships. *BMC Health Services Research* 2012; 12. Article. DOI: 10.1186/1472-6963-12-152.

107. Foxworth R, Redvers N, Moreno MA, et al. Covid-19 vaccination in American Indians and Alaska natives — Lessons from effective community responses. *New England Journal of Medicine* 2021; 385: 2403-2406. Short Survey. DOI: 10.1056/NEJMp2113296.

108. Foulds HJA, Rodgers CD, Duncan V, et al. A systematic review and meta-analysis of screen time behaviour among North American indigenous populations. *Obesity Reviews* 2016; 17: 455-466. Article. DOI: 10.1111/obr.12389.

109. Firestone R, Cheng S, Dalhousie S, et al. Exploring Pasifika wellbeing: findings from a large cluster randomised controlled trial of a mobile health intervention programme. *The New Zealand medical journal* 2020; 133: 82-101. Article.

110. Feldman JM, Gruskin S, Coull BA, et al. Quantifying underreporting of law-enforcement-related deaths in United States vital statistics and news-media-based data sources: A capture–recapture analysis. *PLoS Medicine* 2017; 14. Article. DOI: 10.1371/journal.pmed.1002399.

111. Eades S, Eades F, McCaullay D, et al. Australia's First Nations' response to the COVID-19 pandemic. *The Lancet* 2020; 396: 237-238. Letter. DOI: 10.1016/S0140-6736(20)31545-2.

112. Dingwall KM, Sweet M, Cass A, et al. Effectiveness of Wellbeing Intervention for Chronic Kidney Disease (WICKD): results of a randomised controlled trial. *BMC Nephrology* 2021; 22. Article. DOI: 10.1186/s12882-021-02344-8.

113. Danchin M and Buttery J. COVID-19 vaccine hesitancy: a unique set of challenges. *Internal Medicine Journal* 2021; 51: 1987-1989. Editorial. DOI: 10.1111/imj.15599.

114. D’Amico EJ, Dickerson DL, Rodriguez A, et al. Integrating traditional practices and social network visualization to prevent substance use: study protocol for a randomized controlled trial among urban Native American emerging adults. *Addiction Science and Clinical Practice* 2021; 16. Article. DOI: 10.1186/s13722-021-00265-3.

115. Christie V, Rice M, Dracakis J, et al. Improving breast cancer outcomes for Aboriginal women: A mixed-methods study protocol. *BMJ Open* 2022; 12. Article. DOI: 10.1136/bmjopen-2020-048003.

116. Christensen H and Petrie K. Online mental health programs promising tools for suicide prevention. *Medicine Today* 2014; 15: 66-68. Article.

117. Chepulis L, Morison B, Tamatea J, et al. Midwifery awareness of diabetes in pregnancy screening guidelines in Aotearoa New Zealand. *Midwifery* 2022; 106. Article. DOI: 10.1016/j.midw.2021.103230.

118. Chamberlain C, Perlen S, Brennan S, et al. Evidence for a comprehensive approach to Aboriginal tobacco control to maintain the decline in smoking: An overview of reviews among Indigenous peoples. *Systematic Reviews* 2017; 6. Article. DOI: 10.1186/s13643-017-0520-9.

119. Carson SL, Casillas A, Castellon-Lopez Y, et al. COVID-19 Vaccine Decision-making Factors in Racial and Ethnic Minority Communities in Los Angeles, California. *JAMA Network Open* 2021; 4. Article. DOI: 10.1001/jamanetworkopen.2021.27582.

120. Carlson B, Frazer R and Farrelly T. “That makes all the difference”: Aboriginal and Torres Strait Islander health-seeking on social media. *Health Promotion Journal of Australia* 2021; 32: 523-531. Article. DOI: 10.1002/hpja.366.

121. Callander D, Schneider JA, Radix A, et al. Longitudinal cohort of HIV-negative transgender women of colour in New York City: Protocol for the TURNNT ('Trying to Understand Relationships, Networks and Neighbourhoods among Transgender women of colour') study. *BMJ Open* 2020; 10. Article. DOI: 10.1136/bmjopen-2019-032876.

122. Brusse C, Gardner K, McAullay D, et al. Social media and mobile apps for health promotion in Australian indigenous populations: Scoping review. *Journal of Medical Internet Research* 2014; 16. Review. DOI: 10.2196/jmir.3614.

123. Britt RK, Britt BC, Anderson J, et al. “Sharing Hope and Healing”: A Culturally Tailored Social Media Campaign to Promote Living Kidney Donation and Transplantation Among Native Americans. *Health Promotion Practice* 2021; 22: 786-795. Article. DOI: 10.1177/1524839920974580.

124. Brennan DJ, Lachowsky NJ, Georgievski G, et al. Online outreach services among men who use the internet to seek sex with other men (MISM) in Ontario, Canada: An online survey. *Journal of Medical Internet Research* 2015; 17. Article. DOI: 10.2196/jmir.4503.

125. Berends L and Halliday R. Capacity building and social marketing promotes healthy lifestyle behaviour in an australian aboriginal community. *Australian Journal of Rural Health* 2018; 26: 279-283. Article. DOI: 10.1111/ajr.12421.

126. Bennett-Levy J, Singer J, DuBois S, et al. Translating e-mental health into practice: What are the barriers and enablers to e-mental health implementation by aboriginal and torres strait islander health professionals? *Journal of Medical Internet Research* 2017; 19. Article. DOI: 10.2196/jmir.6269.

127. Benatar J, Evile T and Wihongi H. Hui: a partnership in practice in familial hypercholesterolemia. *The New Zealand medical journal* 2020; 133: 63-70. Article.

128. Attwell K, Carlson S, Tchilingirian J, et al. Coronavax: Preparing community and government for COVID-19 vaccination: A research protocol for a mixed methods social research project. *BMJ Open* 2021; 11. Article. DOI: 10.1136/bmjopen-2021-049356.

129. Arnaudova I, Jin H and Amaro H. Pretreatment social network characteristics relate to increased risk of dropout and unfavorable outcomes among women in a residential treatment setting for substance use. *Journal of Substance Abuse Treatment* 2020; 116. Article. DOI: 10.1016/j.jsat.2020.108044.

130. Arambula Solomon TG, Jones D, Laurila K, et al. Using the Community Readiness Model to Assess American Indian Communities Readiness to Address Cancer Prevention and Control Programs. *Journal of Cancer Education* 2021. Article. DOI: 10.1007/s13187-021-02100-4.

131. Aoki JR and Porter MA. First Food Policy and Law Scan: How Tribes in the Bemidji Area Are Applying Policy and Systems Approaches to Support Breastfeeding. *Preventing Chronic Disease* 2021; 18: 1-4. Article. DOI: 10.5888/PCD18.200460.

132. Anderson K, Gall A, Butler T, et al. Using web conferencing to engage Aboriginal and Torres Strait Islander young people in research: a feasibility study. *BMC Medical Research Methodology* 2021; 21. Article. DOI: 10.1186/s12874-021-01366-y.

133. Anderson G, Gleeson S, Rissel C, et al. Twitter tweets and twaddle: Twittering at AHPA's National Health Promotion Conference. *Health Promotion Journal of Australia* 2014; 25: 143-146. Article. DOI: 10.1071/HE13112.

134. Ali-Hassan H, Eloulabi R and Keethakumar A. Internet non-use among Canadian indigenous older adults: Aboriginal Peoples Survey (APS). *BMC Public Health* 2020; 20. Article. DOI: 10.1186/s12889-020-09659-5.

135. Alexander J, Kwon HT, Strecher R, et al. Multicultural media outreach: Increasing cancer information coverage in minority communities. *Journal of Cancer Education* 2013; 28: 744-747. Article. DOI: 10.1007/s13187-013-0534-5.

# CINHAL 2022.03.08

## Notes about this database search

## Actual search terms for CINHAL

Search ID# Search Terms Search Options Actions

S188 S150 AND S162 AND S185 Limiters - Published Date: 20050101-20221231; English Language

Expanders - Apply equivalent subjects

Search modes - Boolean/Phrase View Results (212)

View Details

Edit

S187 S150 AND S162 AND S185 Limiters - Published Date: 20050101-20221231

Expanders - Apply equivalent subjects

Search modes - Boolean/Phrase Rerun

View Details

Edit

S186 S150 AND S162 AND S185 Expanders - Apply equivalent subjects

Search modes - Boolean/Phrase Rerun

View Details

Edit

S185 S163 OR S164 OR S165 OR S166 OR S167 OR S168 OR S169 OR S170 OR S171 OR S172 OR S173 OR S174 OR S175 OR S176 OR S177 OR S178 OR S179 OR S180 OR S181 OR S182 OR S183 OR S184 Expanders - Apply equivalent subjects

Search modes - Boolean/Phrase Rerun

View Details

Edit

S184 "addiction disorders" Expanders - Apply equivalent subjects

Search modes - Boolean/Phrase Rerun

View Details

Edit

S183 (MH "Tobacco+") Expanders - Apply equivalent subjects

Search modes - Boolean/Phrase Rerun

View Details

Edit

S182 (MH "Substance Use Disorders+") Expanders - Apply equivalent subjects

Search modes - Boolean/Phrase Rerun

View Details

Edit

S181 (MH "Alcoholism") OR (MH "Alcohol Abuse+") Expanders - Apply equivalent subjects

Search modes - Boolean/Phrase Rerun

View Details

Edit

S180 (MH "Stress, Psychological+") Expanders - Apply equivalent subjects

Search modes - Boolean/Phrase Rerun

View Details

Edit

S179 (MH "Diabetes Mellitus+") OR (MH "Diabetes Mellitus, Type 2") OR (MH "Diabetes Mellitus, Type 1+") Expanders - Apply equivalent subjects

Search modes - Boolean/Phrase Rerun

View Details

Edit

S178 (MH "Cardiovascular Diseases+") Expanders - Apply equivalent subjects

Search modes - Boolean/Phrase Rerun

View Details

Edit

S177 (MH "Emphysema+") Expanders - Apply equivalent subjects

Search modes - Boolean/Phrase Rerun

View Details

Edit

S176 "multiple chronic conditions" Expanders - Apply equivalent subjects

Search modes - Boolean/Phrase Rerun

View Details

Edit

S175 (MH "Chronic Disease+") Expanders - Apply equivalent subjects

Search modes - Boolean/Phrase Rerun

View Details

Edit

S174 (MH "Lung Diseases+") OR (MH "Pulmonary Disease, Chronic Obstructive+") OR (MH "Lung Diseases, Obstructive+") Expanders - Apply equivalent subjects

Search modes - Boolean/Phrase Rerun

View Details

Edit

S173 "neoplasms or cancer" Expanders - Apply equivalent subjects

Search modes - Boolean/Phrase Rerun

View Details

Edit

S172 (MH "Self Care+") Expanders - Apply equivalent subjects

Search modes - Boolean/Phrase Rerun

View Details

Edit

S171 "wellbeing or well-being or well being" Expanders - Apply equivalent subjects

Search modes - SmartText Searching Rerun

View Details

Edit

S170 (MH "Quality of Life+") Expanders - Apply equivalent subjects

Search modes - Boolean/Phrase Rerun

View Details

Edit

S169 (MH "Smoking+") OR (MH "Smoking Cessation Programs") Expanders - Apply equivalent subjects

Search modes - Boolean/Phrase Rerun

View Details

Edit

S168 (MH "Sex Education") Expanders - Apply equivalent subjects

Search modes - Boolean/Phrase Rerun

View Details

Edit

S167 (MH "Weight Reduction Programs") Expanders - Apply equivalent subjects

Search modes - Boolean/Phrase Rerun

View Details

Edit

S166 "healthy people programs" Expanders - Apply equivalent subjects

Search modes - Boolean/Phrase Rerun

View Details

Edit

S165 (MH "Health Promotion+") Expanders - Apply equivalent subjects

Search modes - Boolean/Phrase Rerun

View Details

Edit

S164 (MH "Health Education+") OR (MH "Health Knowledge") OR (MH "Health Literacy") Expanders - Apply equivalent subjects

Search modes - Boolean/Phrase Rerun

View Details

Edit

S163 (MH "Consumer Health Information+") Expanders - Apply equivalent subjects

Search modes - Boolean/Phrase Rerun

View Details

Edit

S162 S151 OR S152 OR S153 OR S154 OR S155 OR S156 OR S157 OR S158 OR S159 OR S160 OR S161 Expanders - Apply equivalent subjects

Search modes - Boolean/Phrase Rerun

View Details

Edit

S161 "e-health" Expanders - Apply equivalent subjects

Search modes - Boolean/Phrase Rerun

View Details

Edit

S160 "ehealth" Expanders - Apply equivalent subjects

Search modes - Boolean/Phrase Rerun

View Details

Edit

S159 (MH "Twitter") OR (MH "Facebook") OR "facebook or twitter or TicToc or instagram or Pinterest or youtube" Expanders - Apply equivalent subjects

Search modes - Boolean/Phrase Rerun

View Details

Edit

S158 (MH "Health Literacy") Expanders - Apply equivalent subjects

Search modes - Boolean/Phrase Rerun

View Details

Edit

S157 (MH "Mobile Applications") Expanders - Apply equivalent subjects

Search modes - Boolean/Phrase Rerun

View Details

Edit

S156 (MH "Smartphone") Expanders - Apply equivalent subjects

Search modes - Boolean/Phrase Rerun

View Details

Edit

S155 (MH "Social Networking+") OR (MH "Online Social Networking") Expanders - Apply equivalent subjects

Search modes - Boolean/Phrase Rerun

View Details

Edit

S154 (MH "Internet+") OR (MH "Internet-Based Intervention") OR (MH "Internet Access") Expanders - Apply equivalent subjects

Search modes - Boolean/Phrase Rerun

View Details

Edit

S153 (MH "Email") Expanders - Apply equivalent subjects

Search modes - Boolean/Phrase Rerun

View Details

Edit

S152 (MH "Social Media+") Expanders - Apply equivalent subjects

Search modes - Boolean/Phrase Rerun

View Details

Edit

S151 (MH "Blogs") Expanders - Apply equivalent subjects

Search modes - Boolean/Phrase Rerun

View Details

Edit

S150 S142 OR S143 OR S144 OR S145 OR S146 OR S147 OR S148 OR S149 Expanders - Apply equivalent subjects

Search modes - Boolean/Phrase Rerun

View Details

Edit

S149 "ainu people" Expanders - Apply equivalent subjects

Search modes - Boolean/Phrase Rerun

View Details

Edit

S148 (MH "Native Americans+") OR (MH "Alaska Natives") Expanders - Apply equivalent subjects

Search modes - Boolean/Phrase Rerun

View Details

Edit

S147 (MH "Inuit") Expanders - Apply equivalent subjects

Search modes - Boolean/Phrase Rerun

View Details

Edit

S146 (MH "Inuit") Expanders - Apply equivalent subjects

Search modes - Boolean/Phrase Rerun

View Details

Edit

S145 (MH "Arctic Peoples+") Expanders - Apply equivalent subjects

Search modes - Boolean/Phrase Rerun

View Details

Edit

S144 (MH "Maori") Expanders - Apply equivalent subjects

Search modes - Boolean/Phrase Rerun

View Details

Edit

S143 (MH "Torres Strait Islanders") Expanders - Apply equivalent subjects

Search modes - Boolean/Phrase Rerun

View Details

Edit

S142 (MH "Aboriginal Australians") OR (MH "Native Americans+") OR (MH "First Nations of Australia+") OR (MH "Aboriginal Canadians+") OR (MH "Indigenous Peoples+") OR (MH "Alaska Natives") Expanders - Apply equivalent subjects

Search modes - Boolean/Phrase Rerun

View Details

Edit

S141 S103 AND S115 AND S138 Limiters - Published Date: 20050101-20221231; English Language

Expanders - Apply equivalent subjects

Search modes - Boolean/Phrase Rerun

View Details

Edit

S140 S103 AND S115 AND S138 Limiters - Published Date: 20050101-20221231

Expanders - Apply equivalent subjects

Search modes - Boolean/Phrase Rerun

View Details

Edit

S139 S103 AND S115 AND S138 Expanders - Apply equivalent subjects

Search modes - Boolean/Phrase Rerun

View Details

Edit

S138 S116 OR S117 OR S118 OR S119 OR S120 OR S121 OR S122 OR S123 OR S124 OR S125 OR S126 OR S127 OR S128 OR S129 OR S130 OR S131 OR S132 OR S133 OR S134 OR S135 OR S136 OR S137 Expanders - Apply equivalent subjects

Search modes - Boolean/Phrase Rerun

View Details

Edit

S137 "addiction disorders" Expanders - Apply equivalent subjects

Search modes - Boolean/Phrase Rerun

View Details

Edit

S136 (MH "Tobacco+") Expanders - Apply equivalent subjects

Search modes - Boolean/Phrase Rerun

View Details

Edit

S135 (MH "Substance Use Disorders+") Expanders - Apply equivalent subjects

Search modes - Boolean/Phrase Rerun

View Details

Edit

S134 (MH "Alcoholism") OR (MH "Alcohol Abuse+") Expanders - Apply equivalent subjects

Search modes - Boolean/Phrase Rerun

View Details

Edit

S133 (MH "Stress, Psychological+") Expanders - Apply equivalent subjects

Search modes - Boolean/Phrase Rerun

View Details

Edit

S132 (MH "Diabetes Mellitus+") OR (MH "Diabetes Mellitus, Type 2") OR (MH "Diabetes Mellitus, Type 1+") Expanders - Apply equivalent subjects

Search modes - Boolean/Phrase Rerun

View Details

Edit

S131 (MH "Cardiovascular Diseases+") Expanders - Apply equivalent subjects

Search modes - Boolean/Phrase Rerun

View Details

Edit

S130 (MH "Emphysema+") Expanders - Apply equivalent subjects

Search modes - Boolean/Phrase Rerun

View Details

Edit

S129 "multiple chronic conditions" Expanders - Apply equivalent subjects

Search modes - Boolean/Phrase Rerun

View Details

Edit

S128 (MH "Chronic Disease+") Expanders - Apply equivalent subjects

Search modes - Boolean/Phrase Rerun

View Details

Edit

S127 (MH "Lung Diseases+") OR (MH "Pulmonary Disease, Chronic Obstructive+") OR (MH "Lung Diseases, Obstructive+") Expanders - Apply equivalent subjects

Search modes - Boolean/Phrase Rerun

View Details

Edit

S126 "neoplasms or cancer" Expanders - Apply equivalent subjects

Search modes - Boolean/Phrase Rerun

View Details

Edit

S125 (MH "Self Care+") Expanders - Apply equivalent subjects

Search modes - Boolean/Phrase Rerun

View Details

Edit

S124 "wellbeing or well-being or well being" Expanders - Apply equivalent subjects

Search modes - SmartText Searching Rerun

View Details

Edit

S123 (MH "Quality of Life+") Expanders - Apply equivalent subjects

Search modes - Boolean/Phrase Rerun

View Details

Edit

S122 (MH "Smoking+") OR (MH "Smoking Cessation Programs") Expanders - Apply equivalent subjects

Search modes - Boolean/Phrase Rerun

View Details

Edit

S121 (MH "Sex Education") Expanders - Apply equivalent subjects

Search modes - Boolean/Phrase Rerun

View Details

Edit

S120 (MH "Weight Reduction Programs") Expanders - Apply equivalent subjects

Search modes - Boolean/Phrase Rerun

View Details

Edit

S119 "healthy people programs" Expanders - Apply equivalent subjects

Search modes - Boolean/Phrase Rerun

View Details

Edit

S118 (MH "Health Promotion+") Expanders - Apply equivalent subjects

Search modes - Boolean/Phrase Rerun

View Details

Edit

S117 (MH "Health Education+") OR (MH "Health Knowledge") OR (MH "Health Literacy") Expanders - Apply equivalent subjects

Search modes - Boolean/Phrase Rerun

View Details

Edit

S116 (MH "Consumer Health Information+") Expanders - Apply equivalent subjects

Search modes - Boolean/Phrase Rerun

View Details

Edit

S115 S104 OR S105 OR S106 OR S107 OR S108 OR S109 OR S110 OR S111 OR S112 OR S113 OR S114 Expanders - Apply equivalent subjects

Search modes - Boolean/Phrase Rerun

View Details

Edit

S114 "e-health" Expanders - Apply equivalent subjects

Search modes - Boolean/Phrase Rerun

View Details

Edit

S113 "ehealth" Expanders - Apply equivalent subjects

Search modes - Boolean/Phrase Rerun

View Details

Edit

S112 (MH "Twitter") OR (MH "Facebook") OR "facebook or twitter or TicToc or instagram or Pinterest or youtube" Expanders - Apply equivalent subjects

Search modes - Boolean/Phrase Rerun

View Details

Edit

S111 (MH "Health Literacy") Expanders - Apply equivalent subjects

Search modes - Boolean/Phrase Rerun

View Details

Edit

S110 (MH "Mobile Applications") Expanders - Apply equivalent subjects

Search modes - Boolean/Phrase Rerun

View Details

Edit

S109 (MH "Smartphone") Expanders - Apply equivalent subjects

Search modes - Boolean/Phrase Rerun

View Details

Edit

S108 (MH "Social Networking+") OR (MH "Online Social Networking") Expanders - Apply equivalent subjects

Search modes - Boolean/Phrase Rerun

View Details

Edit

S107 (MH "Internet+") OR (MH "Internet-Based Intervention") OR (MH "Internet Access") Expanders - Apply equivalent subjects

Search modes - Boolean/Phrase Rerun

View Details

Edit

S106 (MH "Email") Expanders - Apply equivalent subjects

Search modes - Boolean/Phrase Rerun

View Details

Edit

S105 (MH "Social Media+") Expanders - Apply equivalent subjects

Search modes - Boolean/Phrase Rerun

View Details

Edit

S104 (MH "Blogs") Expanders - Apply equivalent subjects

Search modes - Boolean/Phrase Rerun

View Details

Edit

S103 S95 OR S96 OR S97 OR S98 OR S99 OR S100 OR S101 OR S102 Expanders - Apply equivalent subjects

Search modes - Boolean/Phrase Rerun

View Details

Edit

S102 "ainu people" Expanders - Apply equivalent subjects

Search modes - Boolean/Phrase Rerun

View Details

Edit

S101 (MH "Native Americans+") OR (MH "Alaska Natives") Expanders - Apply equivalent subjects

Search modes - Boolean/Phrase Rerun

View Details

Edit

S100 (MH "Inuit") Expanders - Apply equivalent subjects

Search modes - Boolean/Phrase Rerun

View Details

Edit

S99 (MH "Inuit") Expanders - Apply equivalent subjects

Search modes - Boolean/Phrase Rerun

View Details

Edit

S98 (MH "Arctic Peoples+") Expanders - Apply equivalent subjects

Search modes - Boolean/Phrase Rerun

View Details

Edit

S97 (MH "Maori") Expanders - Apply equivalent subjects

Search modes - Boolean/Phrase Rerun

View Details

Edit

S96 (MH "Torres Strait Islanders") Expanders - Apply equivalent subjects

Search modes - Boolean/Phrase Rerun

View Details

Edit

S95 (MH "Aboriginal Australians") OR (MH "Native Americans+") OR (MH "First Nations of Australia+") OR (MH "Aboriginal Canadians+") OR (MH "Indigenous Peoples+") OR (MH "Alaska Natives") Expanders - Apply equivalent subjects

Search modes - Boolean/Phrase Rerun

View Details

Edit

S94 S56 AND S68 AND S91 Limiters - Published Date: 20050101-20221231; English Language

Expanders - Apply equivalent subjects

Search modes - Boolean/Phrase View Results (212)

View Details

Edit

S93 S56 AND S68 AND S91 Limiters - Published Date: 20050101-20221231

Expanders - Apply equivalent subjects

Search modes - Boolean/Phrase Rerun

View Details

Edit

S92 S56 AND S68 AND S91 Expanders - Apply equivalent subjects

Search modes - Boolean/Phrase Rerun

View Details

Edit

S91 S69 OR S70 OR S71 OR S72 OR S73 OR S74 OR S75 OR S76 OR S77 OR S78 OR S79 OR S80 OR S81 OR S82 OR S83 OR S84 OR S85 OR S86 OR S87 OR S88 OR S89 OR S90 Expanders - Apply equivalent subjects

Search modes - Boolean/Phrase Rerun

View Details

Edit

S90 "addiction disorders" Expanders - Apply equivalent subjects

Search modes - Boolean/Phrase Rerun

View Details

Edit

S89 (MH "Tobacco+") Expanders - Apply equivalent subjects

Search modes - Boolean/Phrase Rerun

View Details

Edit

S88 (MH "Substance Use Disorders+") Expanders - Apply equivalent subjects

Search modes - Boolean/Phrase Rerun

View Details

Edit

S87 (MH "Alcoholism") OR (MH "Alcohol Abuse+") Expanders - Apply equivalent subjects

Search modes - Boolean/Phrase Rerun

View Details

Edit

S86 (MH "Stress, Psychological+") Expanders - Apply equivalent subjects

Search modes - Boolean/Phrase Rerun

View Details

Edit

S85 (MH "Diabetes Mellitus+") OR (MH "Diabetes Mellitus, Type 2") OR (MH "Diabetes Mellitus, Type 1+") Expanders - Apply equivalent subjects

Search modes - Boolean/Phrase Rerun

View Details

Edit

S84 (MH "Cardiovascular Diseases+") Expanders - Apply equivalent subjects

Search modes - Boolean/Phrase Rerun

View Details

Edit

S83 (MH "Emphysema+") Expanders - Apply equivalent subjects

Search modes - Boolean/Phrase Rerun

View Details

Edit

S82 "multiple chronic conditions" Expanders - Apply equivalent subjects

Search modes - Boolean/Phrase Rerun

View Details

Edit

S81 (MH "Chronic Disease+") Expanders - Apply equivalent subjects

Search modes - Boolean/Phrase Rerun

View Details

Edit

S80 (MH "Lung Diseases+") OR (MH "Pulmonary Disease, Chronic Obstructive+") OR (MH "Lung Diseases, Obstructive+") Expanders - Apply equivalent subjects

Search modes - Boolean/Phrase Rerun

View Details

Edit

S79 "neoplasms or cancer" Expanders - Apply equivalent subjects

Search modes - Boolean/Phrase Rerun

View Details

Edit

S78 (MH "Self Care+") Expanders - Apply equivalent subjects

Search modes - Boolean/Phrase Rerun

View Details

Edit

S77 "wellbeing or well-being or well being" Expanders - Apply equivalent subjects

Search modes - SmartText Searching Rerun

View Details

Edit

S76 (MH "Quality of Life+") Expanders - Apply equivalent subjects

Search modes - Boolean/Phrase Rerun

View Details

Edit

S75 (MH "Smoking+") OR (MH "Smoking Cessation Programs") Expanders - Apply equivalent subjects

Search modes - Boolean/Phrase Rerun

View Details

Edit

S74 (MH "Sex Education") Expanders - Apply equivalent subjects

Search modes - Boolean/Phrase Rerun

View Details

Edit

S73 (MH "Weight Reduction Programs") Expanders - Apply equivalent subjects

Search modes - Boolean/Phrase Rerun

View Details

Edit

S72 "healthy people programs" Expanders - Apply equivalent subjects

Search modes - Boolean/Phrase Rerun

View Details

Edit

S71 (MH "Health Promotion+") Expanders - Apply equivalent subjects

Search modes - Boolean/Phrase Rerun

View Details

Edit

S70 (MH "Health Education+") OR (MH "Health Knowledge") OR (MH "Health Literacy") Expanders - Apply equivalent subjects

Search modes - Boolean/Phrase Rerun

View Details

Edit

S69 (MH "Consumer Health Information+") Expanders - Apply equivalent subjects

Search modes - Boolean/Phrase Rerun

View Details

Edit

S68 S57 OR S58 OR S59 OR S60 OR S61 OR S62 OR S63 OR S64 OR S65 OR S66 OR S67 Expanders - Apply equivalent subjects

Search modes - Boolean/Phrase Rerun

View Details

Edit

S67 "e-health" Expanders - Apply equivalent subjects

Search modes - Boolean/Phrase Rerun

View Details

Edit

S66 "ehealth" Expanders - Apply equivalent subjects

Search modes - Boolean/Phrase Rerun

View Details

Edit

S65 (MH "Twitter") OR (MH "Facebook") OR "facebook or twitter or TicToc or instagram or Pinterest or youtube" Expanders - Apply equivalent subjects

Search modes - Boolean/Phrase Rerun

View Details

Edit

S64 (MH "Health Literacy") Expanders - Apply equivalent subjects

Search modes - Boolean/Phrase Rerun

View Details

Edit

S63 (MH "Mobile Applications") Expanders - Apply equivalent subjects

Search modes - Boolean/Phrase Rerun

View Details

Edit

S62 (MH "Smartphone") Expanders - Apply equivalent subjects

Search modes - Boolean/Phrase Rerun

View Details

Edit

S61 (MH "Social Networking+") OR (MH "Online Social Networking") Expanders - Apply equivalent subjects

Search modes - Boolean/Phrase Rerun

View Details

Edit

S60 (MH "Internet+") OR (MH "Internet-Based Intervention") OR (MH "Internet Access") Expanders - Apply equivalent subjects

Search modes - Boolean/Phrase Rerun

View Details

Edit

S59 (MH "Email") Expanders - Apply equivalent subjects

Search modes - Boolean/Phrase Rerun

View Details

Edit

S58 (MH "Social Media+") Expanders - Apply equivalent subjects

Search modes - Boolean/Phrase Rerun

View Details

Edit

S57 (MH "Blogs") Expanders - Apply equivalent subjects

Search modes - Boolean/Phrase Rerun

View Details

Edit

S56 S48 OR S49 OR S50 OR S51 OR S52 OR S53 OR S54 OR S55 Expanders - Apply equivalent subjects

Search modes - Boolean/Phrase Rerun

View Details

Edit

S55 "ainu people" Expanders - Apply equivalent subjects

Search modes - Boolean/Phrase Rerun

View Details

Edit

S54 (MH "Native Americans+") OR (MH "Alaska Natives") Expanders - Apply equivalent subjects

Search modes - Boolean/Phrase Rerun

View Details

Edit

S53 (MH "Inuit") Expanders - Apply equivalent subjects

Search modes - Boolean/Phrase Rerun

View Details

Edit

S52 (MH "Inuit") Expanders - Apply equivalent subjects

Search modes - Boolean/Phrase Rerun

View Details

Edit

S51 (MH "Arctic Peoples+") Expanders - Apply equivalent subjects

Search modes - Boolean/Phrase Rerun

View Details

Edit

S50 (MH "Maori") Expanders - Apply equivalent subjects

Search modes - Boolean/Phrase Rerun

View Details

Edit

S49 (MH "Torres Strait Islanders") Expanders - Apply equivalent subjects

Search modes - Boolean/Phrase Rerun

View Details

Edit

S48 (MH "Aboriginal Australians") OR (MH "Native Americans+") OR (MH "First Nations of Australia+") OR (MH "Aboriginal Canadians+") OR (MH "Indigenous Peoples+") OR (MH "Alaska Natives") Expanders - Apply equivalent subjects

Search modes - Boolean/Phrase Rerun

View Details

Edit

S47 S9 AND S21 AND S44 Limiters - Published Date: 20050101-20221231; English Language

Expanders - Apply equivalent subjects

Search modes - Boolean/Phrase View Results (212)

View Details

Edit

S46 S9 AND S21 AND S44 Limiters - Published Date: 20050101-20221231

Expanders - Apply equivalent subjects

Search modes - Boolean/Phrase View Results (212)

View Details

Edit

S45 S9 AND S21 AND S44 Expanders - Apply equivalent subjects

Search modes - Boolean/Phrase View Results (229)

View Details

Edit

S44 S22 OR S23 OR S24 OR S25 OR S26 OR S27 OR S28 OR S29 OR S30 OR S31 OR S32 OR S33 OR S34 OR S35 OR S36 OR S37 OR S38 OR S39 OR S40 OR S41 OR S42 OR S43 Expanders - Apply equivalent subjects

Search modes - Boolean/Phrase View Results (1,583,096)

View Details

Edit

S43 "addiction disorders" Expanders - Apply equivalent subjects

Search modes - Boolean/Phrase View Results (103)

View Details

Edit

S42 (MH "Tobacco+") Expanders - Apply equivalent subjects

Search modes - Boolean/Phrase View Results (10,166)

View Details

Edit

S41 (MH "Substance Use Disorders+") Expanders - Apply equivalent subjects

Search modes - Boolean/Phrase View Results (178,769)

View Details

Edit

S40 (MH "Alcoholism") OR (MH "Alcohol Abuse+") Expanders - Apply equivalent subjects

Search modes - Boolean/Phrase View Results (30,142)

View Details

Edit

S39 (MH "Stress, Psychological+") Expanders - Apply equivalent subjects

Search modes - Boolean/Phrase View Results (94,664)

View Details

Edit

S38 (MH "Diabetes Mellitus+") OR (MH "Diabetes Mellitus, Type 2") OR (MH "Diabetes Mellitus, Type 1+") Expanders - Apply equivalent subjects

Search modes - Boolean/Phrase View Results (181,750)

View Details

Edit

S37 (MH "Cardiovascular Diseases+") Expanders - Apply equivalent subjects

Search modes - Boolean/Phrase View Results (648,887)

View Details

Edit

S36 (MH "Emphysema+") Expanders - Apply equivalent subjects

Search modes - Boolean/Phrase View Results (4,066)

View Details

Edit

S35 "multiple chronic conditions" Expanders - Apply equivalent subjects

Search modes - Boolean/Phrase View Results (948)

View Details

Edit

S34 (MH "Chronic Disease+") Expanders - Apply equivalent subjects

Search modes - Boolean/Phrase View Results (70,421)

View Details

Edit

S33 (MH "Lung Diseases+") OR (MH "Pulmonary Disease, Chronic Obstructive+") OR (MH "Lung Diseases, Obstructive+") Expanders - Apply equivalent subjects

Search modes - Boolean/Phrase View Results (207,079)

View Details

Edit

S32 "neoplasms or cancer" Expanders - Apply equivalent subjects

Search modes - Boolean/Phrase View Results (27)

View Details

Edit

S31 (MH "Self Care+") Expanders - Apply equivalent subjects

Search modes - Boolean/Phrase View Results (57,078)

View Details

Edit

S30 "wellbeing or well-being or well being" Expanders - Apply equivalent subjects

Search modes - SmartText Searching View Results (7,939)

View Details

Edit

S29 (MH "Quality of Life+") Expanders - Apply equivalent subjects

Search modes - Boolean/Phrase View Results (136,926)

View Details

Edit

S28 (MH "Smoking+") OR (MH "Smoking Cessation Programs") Expanders - Apply equivalent subjects

Search modes - Boolean/Phrase View Results (78,065)

View Details

Edit

S27 (MH "Sex Education") Expanders - Apply equivalent subjects

Search modes - Boolean/Phrase View Results (5,892)

View Details

Edit

S26 (MH "Weight Reduction Programs") Expanders - Apply equivalent subjects

Search modes - Boolean/Phrase View Results (3,230)

View Details

Edit

S25 "healthy people programs" Expanders - Apply equivalent subjects

Search modes - Boolean/Phrase View Results (432)

View Details

Edit

S24 (MH "Health Promotion+") Expanders - Apply equivalent subjects

Search modes - Boolean/Phrase View Results (76,651)

View Details

Edit

S23 (MH "Health Education+") OR (MH "Health Knowledge") OR (MH "Health Literacy") Expanders - Apply equivalent subjects

Search modes - Boolean/Phrase View Results (167,046)

View Details

Edit

S22 (MH "Consumer Health Information+") Expanders - Apply equivalent subjects

Search modes - Boolean/Phrase View Results (18,760)

View Details

Edit

S21 S10 OR S11 OR S12 OR S13 OR S14 OR S15 OR S16 OR S17 OR S18 OR S19 OR S20 Expanders - Apply equivalent subjects

Search modes - Boolean/Phrase View Results (197,979)

View Details

Edit

S20 "e-health" Expanders - Apply equivalent subjects

Search modes - Boolean/Phrase View Results (1,619)

View Details

Edit

S19 "ehealth" Expanders - Apply equivalent subjects

Search modes - Boolean/Phrase View Results (0)

View Details

Edit

S18 (MH "Twitter") OR (MH "Facebook") OR "facebook or twitter or TicToc or instagram or Pinterest or youtube" Expanders - Apply equivalent subjects

Search modes - Boolean/Phrase View Results (1,619)

View Details

Edit

S17 (MH "Health Literacy") Expanders - Apply equivalent subjects

Search modes - Boolean/Phrase View Results (5,363)

View Details

Edit

S16 (MH "Mobile Applications") Expanders - Apply equivalent subjects

Search modes - Boolean/Phrase View Results (10,305)

View Details

Edit

S15 (MH "Smartphone") Expanders - Apply equivalent subjects

Search modes - Boolean/Phrase View Results (3,538)

View Details

Edit

S14 (MH "Social Networking+") OR (MH "Online Social Networking") Expanders - Apply equivalent subjects

Search modes - Boolean/Phrase View Results (3,661)

View Details

Edit

S13 (MH "Internet+") OR (MH "Internet-Based Intervention") OR (MH "Internet Access") Expanders - Apply equivalent subjects

Search modes - Boolean/Phrase View Results (162,286)

View Details

Edit

S12 (MH "Email") Expanders - Apply equivalent subjects

Search modes - Boolean/Phrase View Results (6,954)

View Details

Edit

S11 (MH "Social Media+") Expanders - Apply equivalent subjects

Search modes - Boolean/Phrase View Results (19,417)

View Details

Edit

S10 (MH "Blogs") Expanders - Apply equivalent subjects

Search modes - Boolean/Phrase View Results (3,731)

View Details

Edit

S9 S1 OR S2 OR S3 OR S4 OR S5 OR S6 OR S7 OR S8 Expanders - Apply equivalent subjects

Search modes - Boolean/Phrase View Results (22,055)

View Details

Edit

S8 "ainu people" Expanders - Apply equivalent subjects

Search modes - Boolean/Phrase View Results (2)

View Details

Edit

S7 (MH "Native Americans+") OR (MH "Alaska Natives") Expanders - Apply equivalent subjects

Search modes - Boolean/Phrase View Results (8,984)

View Details

Edit

S6 (MH "Inuit") Expanders - Apply equivalent subjects

Search modes - Boolean/Phrase View Results (360)

View Details

Edit

S5 (MH "Inuit") Expanders - Apply equivalent subjects

Search modes - Boolean/Phrase View Results (0)

View Details

Edit

S4 (MH "Arctic Peoples+") Expanders - Apply equivalent subjects

Search modes - Boolean/Phrase View Results (950)

View Details

Edit

S3 (MH "Maori") Expanders - Apply equivalent subjects

Search modes - Boolean/Phrase View Results (1,874)

View Details

Edit

S2 (MH "Torres Strait Islanders") Expanders - Apply equivalent subjects

Search modes - Boolean/Phrase View Results (120)

View Details

Edit

S1 (MH "Aboriginal Australians") OR (MH "Native Americans+") OR (MH "First Nations of Australia+") OR (MH "Aboriginal Canadians+") OR (MH "Indigenous Peoples+") OR (MH "Alaska Natives") Expanders - Apply equivalent subjects

Search modes - Boolean/Phrase View Results (22,055)

View Details

Edit

## Link to saved search for CINHAL

<https://web-p-ebscohost-com.simsrad.net.ocs.mq.edu.au/ehost/folder?vid=166&sid=dc7e0ea1-5888-4745-90e6-5acad236a4a6%40redis>

## References found by CINHAL 2022.03.08

1. Hanson JD, Weber TL, Shrestha U, et al. Acceptability of an eHealth Intervention to Prevent Alcohol‐Exposed Pregnancy Among American Indian/Alaska Native Teens. Alcoholism: Clinical & Experimental Research 2020; 44: 196-202. DOI: 10.1111/acer.14229.

2. Searby A and Burr D. Telehealth during COVID‐19: The perspective of alcohol and other drug nurses. Journal of Advanced Nursing (John Wiley & Sons, Inc) 2021; 77: 3829-3841. DOI: 10.1111/jan.14939.

3. Dawson KG, Jin A, Summerskill M, et al. Mobile Diabetes Telemedicine Clinics for Aboriginal First Nation People With Reported Diabetes in British Columbia. Canadian Journal of Diabetes 2021; 45: 89-95. DOI: 10.1016/j.jcjd.2020.05.018.

4. Yulong GU, Kennely J, Warren J, et al. Identifying eHealth Opportunities to Support Medication Adherence -- Findings of a Focus Group Study. Studies in Health Technology & Informatics 2016; 223: 150-157. DOI: 10.3233/978-1-61499-645-3-150.

5. Ryan T. COMPARING HEALTH OUTCOMES OF RURAL AND URBAN DIABETES PATIENTS: AN AUDIT OF A MĀORI HEALTH PROVIDER. Kai Tiaki Nursing Research 2021; 12: 60-62.

6. Snoswell CL, Caffery LJ, Haydon HM, et al. Telehealth uptake in general practice as a result of the coronavirus (COVID-19) pandemic. Australian Health Review 2020; 44: 737-740. DOI: 10.1071/AH20183.

7. Xu D, Jenkins A, Ryan C, et al. Health‐related behaviours in a remote Indigenous population with Type 2 diabetes: a Central Australian primary care survey in the Telehealth Eye and Associated Medical Services Network [TEAMSnet] project. Diabetic Medicine 2019; 36: 1659-1670. DOI: 10.1111/dme.14099.

8. Tao X and Fisher CB. Exposure to Social Media Racial Discrimination and Mental Health among Adolescents of Color. Journal of Youth & Adolescence 2022; 51: 30-44. DOI: 10.1007/s10964-021-01514-z.

9. Tobe SW, Yeates K, Campbell NRC, et al. Diagnosing hypertension in Indigenous Canadians (DREAM-GLOBAL): A randomized controlled trial to compare the effectiveness of short message service messaging for management of hypertension: Main results. Journal of Clinical Hypertension 2019; 21: 29-36. DOI: 10.1111/jch.13434.

10. Dilekli N, Janitz A, Martinez S, et al. Spatiotemporal Analysis of Oklahoma Tobacco Helpline Registrations Using Geoimputation and Joinpoint Analysis. Journal of Public Health Management & Practice 2019; 25: S61-S69. DOI: 10.1097/PHH.0000000000000996.

11. Crossan D. Health literacy can improve inequities. Kai Tiaki Nursing New Zealand 2020; 26: 22-43.

12. Burhansstipanov L, Krebs LU, Petereit D, et al. Reality Versus Grant Application Research “Plans”. Health Promotion Practice 2018; 19: 566-572. DOI: 10.1177/1524839917700892.

13. Low T, Scott-Chapman S and Forrest R. Patient experiences of pictogram use during nurse-led rapid-access chest pain clinic consultations in regional Aotearoa New Zealand. Nursing Praxis in Aotearoa New Zealand 2020; 36: 31-40. DOI: 10.36951/27034542.2020.009.

14. Petrun Sayers EL, Bouskill KE, Concannon TW, et al. Creating culture-centered health and health insurance literacy resources: lessons learned from Haitian Creole, Mandarin, Native American, and Vietnamese communities. Journal of Communication in Healthcare 2021; 14: 312-323. DOI: 10.1080/17538068.2021.1930814.

15. Liew CL, Yeates J and Lilley SC. Digitized indigenous knowledge collections: Impact on cultural knowledge transmission, social connections, and cultural identity. Journal of the Association for Information Science & Technology 2021; 72: 1575-1592. DOI: 10.1002/asi.24536.

16. Britt RK, Britt BC, Anderson J, et al. "Sharing Hope and Healing": A Culturally Tailored Social Media Campaign to Promote Living Kidney Donation and Transplantation Among Native Americans. Health Promotion Practice 2021; 22: 786-795. DOI: 10.1177/1524839920974580.

17. Rath A, Wong M, Pannuti CM, et al. Cross-cultural adaptation and validation of Malay version of Rapid Estimate of Adult Literacy in Dentistry (MREALD-30) among Orang Asli population in Malaysia. BMC Oral Health 2021; 21: 1-11. DOI: 10.1186/s12903-021-01866-9.

18. Hengstermann M, Díaz-Artiga A, Otzóy-Sucúc R, et al. Developing Visual Messages to Support Liquefied Petroleum Gas Use in Intervention Homes in the Household Air Pollution Intervention Network (HAPIN) Trial in Rural Guatemala. Health Education & Behavior 2021; 48: 651-669. DOI: 10.1177/1090198121996280.

19. Mathieson K, Leafman JS and Horton MB. Access to Digital Communication Technology and Perceptions of Telemedicine for Patient Education among American Indian Patients with Diabetes. Journal of Health Care for the Poor & Underserved 2017; 28: 1522-1536. DOI: 10.1353/hpu.2017.0131.

20. Hammitt LL, Vigil DE and Reid R. Tribal Sovereignty in Research and Community Engagement for a COVID-19 Vaccine Clinical Trial on the Navajo Nation: Beyond a Facebook Town Hall. American Journal of Public Health 2021; 111: 1431-1432. DOI: 10.2105/ajph.2021.306400.

21. Atlas A, Muru-Lanning M, Moyes S, et al. Cell phone and technology use by octogenarians. Journal of Primary Health Care 2020; 12: 35-40. DOI: 10.1071/HC19042.

22. Hospital Uses Telehealth to Monitor At-risk Patients On the Navajo Reservation. Hospital Case Management 2017; 25: 56-57.

23. Gao Y, Ju X and Jamieson L. Associations between dental care approachability and dental attendance among women pregnant with an Indigenous child: a cross-sectional study. BMC Oral Health 2021; 21: 1-10. DOI: 10.1186/s12903-021-01816-5.

24. Soares GH, Santiago PHR, Biazevic MGH, et al. Do network centrality measures predict dental outcomes of Indigenous children over time? International Journal of Paediatric Dentistry 2021; 31: 634-646. DOI: 10.1111/ipd.12749.

25. Rheault H, Coyer F and Bonner A. Chronic disease health literacy in First Nations people: A mixed methods study. Journal of Clinical Nursing (John Wiley & Sons, Inc) 2021; 30: 2683-2695. DOI: 10.1111/jocn.15757.

26. Harfield S, Elliott S, Ramsey L, et al. Using social networking sites to recruit participants: methods of an online survey of sexual health, knowledge and behaviour of young South Australians. Australian & New Zealand Journal of Public Health 2021; 45: 348-354. DOI: 10.1111/1753-6405.13117.

27. Kearns C, Baggott C, Harwood M, et al. Engaging Māori with qualitative healthcare research using an animated comic. Health Promotion International 2021; 36: 1170-1177. DOI: 10.1093/heapro/daaa111.

28. Jayakody A, Carey M, Bryant J, et al. Exploring experiences and perceptions of Aboriginal and Torres Strait Islander peoples readmitted to hospital with chronic disease in New South Wales, Australia: a qualitative study. Australian Health Review 2021; 45: 411-417. DOI: 10.1071/AH20342.

29. Gross-Schulman S, Sklaroff LM, Hertz CC, et al. Safety Evaluation of an Automated Remote Monitoring System for Heart Failure in an Urban, Indigent Population. Population Health Management 2017; 20: 449-457. DOI: 10.1089/pop.2016.0186.

30. Gibson O, Reilly R, Harfield S, et al. Web-based therapeutic interventions for assessing, managing and treating health conditions in Indigenous people: a scoping review protocol. JBI Database of Systematic Reviews & Implementation Reports 2017; 15: 2487-2494. DOI: 10.11124/JBISRIR-2016-003324.

31. Khalil H and Gruis H. Medication safety challenges in Aboriginal Health Care services. Australian Journal of Rural Health 2019; 27: 542-549. DOI: 10.1111/ajr.12554.

32. Ryan T. EXPLORING THE EXPERIENCES OF MĀORI MEN IN A CULTURALLY ENRICHED WELL-BEING PROGRAMME. Kai Tiaki Nursing Research 2019; 10: 22-28.

33. Gunville JA and Williams J. The development of a prenatal care health literacy instrument for American Indian mothers. International Journal of Child Health & Human Development 2019; 12: 337-344.

34. Hefler M, Kerrigan V, Henryks J, et al. Social media and health information sharing among Australian Indigenous people. Health Promotion International 2019; 34: 706-715. DOI: 10.1093/heapro/day018.

35. Podder D, Dasgupta A, Dobe M, et al. Health Care Seeking Behavior in a Scheduled Tribe Community in India: A Mixed Methods Research Using the Framework of Andersen's Behavioral Model. Asia-Pacific Journal of Public Health 2021; 33: 369-377. DOI: 10.1177/1010539521993695.

36. Schroeder SM, Adamsen C and Besse R. The relationship between diabetes and oral health status, and dental visits among American Indian, Alaska Native, and Native Hawaiian elders. Journal of the American Dental Association (JADA) 2021; 152: 293-301. DOI: 10.1016/j.adaj.2020.12.008.

37. Coates D, Saleeba C and Howe D. Mental Health Attitudes and Beliefs in a Community Sample on the Central Coast in Australia: Barriers to Help Seeking. Community Mental Health Journal 2019; 55: 476-486. DOI: 10.1007/s10597-018-0270-8.

38. Brazionis L, Jenkins A, Keech A, et al. An evaluation of the telehealth facilitation of diabetes and cardiovascular care in remote Australian Indigenous communities: - protocol for the telehealth eye and associated medical services network [TEAMSnet] project, a pre-post study design. BMC Health Services Research 2017; 17: 1-12. DOI: 10.1186/s12913-016-1967-4.

39. McPhail-Bell K, Appo N, Haymes A, et al. Deadly Choices empowering Indigenous Australians through social networking sites. Health Promotion International 2018; 33: 770-780. DOI: 10.1093/heapro/dax014.

40. Cassim S, Kidd J, Rolleston A, et al. Hā Ora: Barriers and enablers to early diagnosis of lung cancer in primary healthcare for Māori communities. European Journal of Cancer Care 2021; 30: 1-8. DOI: 10.1111/ecc.13380.

41. Peake RM, Jackson D, Lea J, et al. Meaningful Engagement With Aboriginal Communities Using Participatory Action Research to Develop Culturally Appropriate Health Resources. Journal of Transcultural Nursing 2021; 32: 129-136. DOI: 10.1177/1043659619899999.

42. Dickerson DL, Parker J, Johnson CL, et al. Recruitment and retention in randomized controlled trials with urban American Indian/Alaska Native adolescents: Challenges and lessons learned. Clinical Trials 2021; 18: 83-91. DOI: 10.1177/1740774520971774.

43. Rheault H, Coyer F and Bonner A. Time to listen: Chronic disease yarning with Aboriginal and Torres Strait Islander peoples living in remote Australia. Collegian 2021; 28: 10-17. DOI: 10.1016/j.colegn.2020.06.001.

44. Day A, Casey S, Baird M, et al. Evaluation of the Aboriginal and Torres Strait Islander Mental Health First Aid Program. Australian & New Zealand Journal of Public Health 2021; 45: 46-52. DOI: 10.1111/1753-6405.13064.

45. Skinner J, Dimitropoulos Y, Moir R, et al. A graduate oral health therapist program to support dental service delivery and oral health promotion in Aboriginal communities in New South Wales, Australia. Rural & Remote Health 2021; 21: 1-9. DOI: 10.22605/RRH5789.

46. Jongbloed K, Friedman AJ, Pearce ME, et al. The Cedar Project WelTel mHealth intervention for HIV prevention in young Indigenous people who use illicit drugs: study protocol for a randomized controlled trial. Trials 2016; 17: 1-12. DOI: 10.1186/s13063-016-1250-3.

47. CHLA 2018 CONFERENCE POSTERS. Journal of the Canadian Health Libraries Association (JCHLA) 2018; 39: 105-118. DOI: 10.29173/jchla29379.

48. Long S, Ipsaralexi Y and Jack C. CHLA 2018 CONFERENCE CONTRIBUTED PAPERS. Journal of the Canadian Health Libraries Association (JCHLA) 2018; 39: 89-104.

49. Thewes B, McCaffery K, Davis E, et al. Insufficient evidence on health literacy amongst Indigenous people with cancer: a systematic literature review. Health Promotion International 2018; 33: 195-218. DOI: 10.1093/heapro/daw066.

50. Gendron F, Hancherow A and Norton A. Exploring and revitalizing Indigenous food networks in Saskatchewan, Canada, as a way to improve food security. Health Promotion International 2017; 32: 808-817. DOI: 10.1093/heapro/daw013.

51. Li J and Brar A. The use and impact of digital technologies for and on the mental health and wellbeing of Indigenous people: A systematic review of empirical studies. Computers in Human Behavior 2022; 126: N.PAG-N.PAG. DOI: 10.1016/j.chb.2021.106988.

52. Venter A, Burns R, Hefford M, et al. Results of a telehealth-enabled chronic care management service to support people with long-term conditions at home. Journal of Telemedicine & Telecare 2012; 18: 172-175. DOI: 10.1258/jtt.2012.SFT112.

53. Flicker S, Wilson C, Monchalin R, et al. The Impact of Indigenous Youth Sharing Digital Stories About HIV Activism. Health Promotion Practice 2020; 21: 802-810. DOI: 10.1177/1524839918822268.

54. De La Rosa VY, Hoover J, Du R, et al. Diet quality among pregnant women in the Navajo Birth Cohort Study. Maternal & Child Nutrition 2020; 16: 1-13. DOI: 10.1111/mcn.12961.

55. Power T, Kelly R, Usher K, et al. Living with diabetes and disadvantage: A qualitative, geographical case study. Journal of Clinical Nursing (John Wiley & Sons, Inc) 2020; 29: 2710-2722. DOI: 10.1111/jocn.15295.

56. Nesoff E, Brownstein J, Veazie M, et al. Time-to-Treatment for Myocardial Infarction: Barriers and Facilitators Perceived by American Indians in Three Regions. Journal of Community Health 2017; 42: 129-138. DOI: 10.1007/s10900-016-0239-x.

57. Simpson ML, Berryman K, Oetzel J, et al. A cultural analysis of New Zealand palliative care brochures. Health Promotion International 2016; 31: 839-848. DOI: 10.1093/heapro/dav067.

58. Hayes R. Whānau Ora: A Māori Health Strategy to Support Whānau in Aotearoa. Whitireia Nursing & Health Journal 2016: 25-29.

59. Untitled. Journal of the Canadian Health Libraries Association (JCHLA) 2016; 37: 63-100.

60. Neubrander J and Metcalfe SE. INCREASING DIVERSITY IN OUR SCHOOLS OF NURSING. Journal of Cultural Diversity 2016; 23: 46-49.

61. Wild CEK, O'Sullivan NA, Lee AC, et al. Survey of Barriers and Facilitators to Engagement in a Multidisciplinary Healthy Lifestyles Program for Children. Journal of Nutrition Education & Behavior 2020; 52: 528-534. DOI: 10.1016/j.jneb.2019.10.010.

62. Pagnocca TS, Zank S and Hanazaki N. "The plants have axé": investigating the use of plants in Afro-Brazilian religions of Santa Catarina Island. Journal of Ethnobiology & Ethnomedicine 2020; 16: 1-13. DOI: 10.1186/s13002-020-00372-6.

63. Sitotaw R, Lulekal E and Abate D. Ethnomycological study of edible and medicinal mushrooms in Menge District, Asossa Zone, Benshangul Gumuz Region, Ethiopia. Journal of Ethnobiology & Ethnomedicine 2020; 16: 1-14. DOI: 10.1186/s13002-020-00361-9.

64. Carroll DM, Soto C, Baezconde-Garbanati L, et al. Tobacco Industry Marketing Exposure and Commercial Tobacco Product Use Disparities among American Indians and Alaska Natives. Substance Use & Misuse 2020; 55: 261-270. DOI: 10.1080/10826084.2019.1664589.

65. Kim KK, Ngo V, Gilkison G, et al. Native American Youth Citizen Scientists Uncovering Community Health and Food Security Priorities. Health Promotion Practice 2020; 21: 80-90. DOI: 10.1177/1524839919852098.

66. Patterson K, Clark S, Berrang-Ford L, et al. Acute gastrointestinal illness in an African Indigenous population: the lived experience of Uganda's Batwa. Rural & Remote Health 2020; 20: 124-134. DOI: 10.22605/RRH5141.

67. Styne DM. Childhood obesity in American Indians. Journal of Public Health Management & Practice 2010; 16: 381-387. DOI: 10.1097/PHH.0b013e3181e887ae.

68. Leiter A, Diefenbach MA, Doucette J, et al. Clinical trial awareness: Changes over time and sociodemographic disparities. Clinical Trials 2015; 12: 215-223. DOI: 10.1177/1740774515571917.

69. Mann WC, Belchior P, Tomita MR, et al. Older adults' perception and use of PDAs, home automation system, and home health monitoring system. Topics in Geriatric Rehabilitation 2007; 23: 35-46. DOI: 10.1097/00013614-200701000-00006.

70. Robinson JD, Turner JW, Levine B, et al. Expanding the Walls of the Health Care Encounter: Support and Outcomes for Patients Online. Health Communication 2011; 26: 125-134. DOI: 10.1080/10410236.2010.541990.

71. Kerrigan V, Herdman RM, Thomas DP, et al. 'I still remember your post about buying smokes': a case study of a remote Aboriginal community-controlled health service using Facebook for tobacco control. Australian Journal of Primary Health 2019; 25: 443-448. DOI: 10.1071/PY19008.

72. Peake RM, Jackson D, Lea J, et al. Investigating the processes used to develop and evaluate the effectiveness of health education resources for adult Indigenous people: A literature review. Contemporary Nurse: A Journal for the Australian Nursing Profession 2019; 55: 421-449. DOI: 10.1080/10376178.2019.1633939.

73. Webb Hooper M, Carpenter KM and Salmon EE. Web-Based Tobacco Cessation Interventions and Digital Inequality across US Racial/Ethnic Groups. Ethnicity & Disease 2019; 29: 495-504. DOI: 10.18865/ed.29.3.495.

74. Patel B. Communicating across cultures: proceedings of a workshop to assess health literacy and cross-cultural communication skills. Journal of Pharmacy Practice & Research 2015; 45: 49-56. DOI: 10.1002/jppr.1062.

75. Hodge FS, Line-Itty T and Ellenwood C. Communication Pathways: HPV Information and Message Barriers Reported among American Indian College Students. Californian Journal of Health Promotion 2014; 12: 14-23. DOI: 10.32398/cjhp.v12i3.1577.

76. Pokhrel P and Herzog TA. Historical Trauma and Substance Use among Native Hawaiian College Students. American Journal of Health Behavior 2014; 38: 420-429. DOI: 10.5993/AJHB.38.3.11.

77. Robertson C, Kattelmann K and Ren C. Control of type 2 diabetes mellitus using interactive internet-based support on a Northern Plains Indian reservation: a pilot study. Topics in Clinical Nutrition 2007; 22: 185-193. DOI: 10.1097/01.tin.0000270137.00099.91.

78. Adcock A, Lawton B, MacDonald EJ, et al. Acceptability of self‐taken vaginal HPV sample for cervical screening among an under‐screened Indigenous population. Australian & New Zealand Journal of Obstetrics & Gynaecology 2019; 59: 301-307. DOI: 10.1111/ajo.12933.

79. Simonds VW, Kim FL, LaVeaux D, et al. Guardians of the Living Water: Using a Health Literacy Framework to Evaluate a Child as Change Agent Intervention. Health Education & Behavior 2019; 46: 349-359. DOI: 10.1177/1090198118798676.

80. Carson SL, Casillas A, Castellon-Lopez Y, et al. COVID-19 Vaccine Decision-making Factors in Racial and Ethnic Minority Communities in Los Angeles, California. JAMA Network Open 2021; 4: e2127582-e2127582. DOI: 10.1001/jamanetworkopen.2021.27582.

81. Sofer D. A Beacon in the Labyrinth of the Indian. AJN American Journal of Nursing 2017; 117: 14-14. DOI: 10.1097/01.NAJ.0000515215.07839.39.

82. Desrosiers A, Vine V and Kershaw T. "R U Mad?": Computerized text analysis of affect in social media relates to stress and substance use among ethnic minority emerging adult males. Anxiety, Stress & Coping 2019; 32: 109-123. DOI: 10.1080/10615806.2018.1539964.

83. Filippi M, McCloskey C, Williams C, et al. Perceptions, Barriers, and Suggestions for Creation of a Tobacco and Health Website Among American Indian/Alaska Native College Students. Journal of Community Health 2013; 38: 486-491. DOI: 10.1007/s10900-012-9634-0.

84. Kypri K, McCambridge J, Vater T, et al. Web-based alcohol intervention for Māori university students: double-blind, multi-site randomized controlled trial. Addiction 2013; 108: 331-338. DOI: 10.1111/j.1360-0443.2012.04067.x.

85. Kypri K, McCambridge J, Vater T, et al. Web-based alcohol intervention for Māori university students: double-blind, multi-site randomized controlled trial. Addiction 2013; 108: 331-338. DOI: 10.1111/j.1360-0443.2012.04067.x.

86. Belton S, Kruske S, Jackson Pulver L, et al. Rheumatic heart disease in pregnancy: How can health services adapt to the needs of Indigenous women? A qualitative study. Australian & New Zealand Journal of Obstetrics & Gynaecology 2018; 58: 425-431. DOI: 10.1111/ajo.12744.

87. Wong G, Glover M, McPherson M, et al. Boosting efficacy of nurse-led stop smoking interventions with a quit and win contest: pilot study results. Contemporary Nurse: A Journal for the Australian Nursing Profession 2018; 54: 395-408. DOI: 10.1080/10376178.2018.1539337.

88. Albino J, Tiwari T, Henderson WG, et al. Parental psychosocial factors and childhood caries prevention: Data from an American Indian population. Community Dentistry & Oral Epidemiology 2018; 46: 360-368. DOI: 10.1111/cdoe.12376.

89. Roh S, Burnette CE, Lee Y-S, et al. Breast cancer literacy and health beliefs related to breast cancer screening among American Indian women. Social Work in Health Care 2018; 57: 465-482. DOI: 10.1080/00981389.2018.1455789.

90. Mindfulness app in Aboriginal communities helps improve mental health. Australian Nursing & Midwifery Journal 2019; 26: 9-9.

91. Galati C, Adams R, Graham K, et al. Health Literacy and Written Communication in Skilled Nursing/Subacute Facilities. OTJR: Occupation, Participation & Health 2018; 38: 131-138. DOI: 10.1177/1539449217723896.

92. Maar MA, Seymour A, Sanderson B, et al. Reaching agreement for an Aboriginal e-health research agenda: the Aboriginal Telehealth Knowledge Circle consensus method. Rural & Remote Health 2010; 10: 10p-10p.

93. Kim G, Kim H and Song M-J. Ethnopharmacological implications of quantitative and network analysis for traditional knowledge regarding the medicinal use of animals by indigenous people in Wolchulsan National Park, Korea. Journal of Ethnopharmacology 2018; 213: 1-11. DOI: 10.1016/j.jep.2017.10.033.

94. Black KJ, Morse B, Tuitt N, et al. Beyond Content: Cultural Perspectives on Using the Internet to Deliver a Sexual Health Intervention to American Indian Youth. Journal of Primary Prevention 2018; 39: 59-70. DOI: 10.1007/s10935-017-0497-0.

95. Garcia A, Baethke L, Kaur J, et al. Lessons Learned from Native C.I.R.C.L.E., a Culturally Specific Resource. Journal of Cancer Education 2017; 32: 740-744. DOI: 10.1007/s13187-016-1001-x.

96. Ju X, Brennan D, Parker E, et al. Efficacy of an oral health literacy intervention among Indigenous Australian adults. Community Dentistry & Oral Epidemiology 2017; 45: 413-426. DOI: 10.1111/cdoe.12305.

97. Lakhan P, Askew D, Harris MF, et al. Understanding health talk in an urban Aboriginal and Torres Strait Islander primary healthcare service: a cross-sectional study. Australian Journal of Primary Health 2017; 23: 335-341. DOI: 10.1071/PY16162.

98. Yost KJ, Bauer MC, Buki LP, et al. Adapting a Cancer Literacy Measure for Use Among Navajo Women. Journal of Transcultural Nursing 2017; 28: 278-285. DOI: 10.1177/1043659616628964.

99. Tomayko EJ, Prince RJ, Cronin KA, et al. Healthy Children, Strong Families 2: A randomized controlled trial of a healthy lifestyle intervention for American Indian families designed using community-based approaches. Clinical Trials 2017; 14: 152-161. DOI: 10.1177/1740774516685699.

100. Finlay S and Wenitong M. Aboriginal Community Controlled Health Organisations are taking a leading role in COVID‐19 health communication. Australian & New Zealand Journal of Public Health 2020; 44: 251-252. DOI: 10.1111/1753-6405.13010.

101. Williams K. Linking Research to Clinical Practice Health Literacy and Patient Communication. Journal of Dental Hygiene 2010; 84: 161-164.

102. Johnson R and Withers M. Cultural competence in the emergency department: Clinicians as cultural learners. Emergency Medicine Australasia 2018; 30: 854-856. DOI: 10.1111/1742-6723.13197.

103. Tomas I. Equity for Indigenous peoples in the emergency department: A Māori perspective. Emergency Medicine Australasia 2018; 30: 859-861. DOI: 10.1111/1742-6723.13196.

104. Street JM, Braunack-Mayer AJ, Facey K, et al. Virtual community consultation? Using the literature and weblogs to link community perspectives and health technology assessment. Health Expectations 2008; 11: 189-200. DOI: 10.1111/j.1369-7625.2007.00484.x.

105. Maticka-Tyndale E. Sexuality and sexual health of Canadian adolescents: yesterday, today and tomorrow. Canadian Journal of Human Sexuality 2008; 17: 85-95.

106. Hoffman-Goetz L and Donelle L. Chat room computer-mediated support on health issues for Aboriginal women. Health Care for Women International 2007; 28: 397-418. DOI: 10.1080/07399330601180057.

107. Boyle JR and Boekeloo BO. Perceived parental approval of drinking and its impact on problem drinking behaviors among first-year college students. Journal of American College Health 2006; 54: 238-244. DOI: 10.3200/jach.54.4.238-244.

108. Williams FD, Osorio A and Castaldi L. Consumer Health Resources for Racial and Ethnic Minority Groups. Journal of Consumer Health on the Internet 2017; 21: 271-283. DOI: 10.1080/15398285.2017.1353315.

109. Vander Schaaf EB, Dellon EP, Carr RA, et al. Perceptions of the Medical Home by Parents of Children With Chronic Illnesses. American Journal of Managed Care 2017; 23: e70-e74.

110. Shegog R, Rushing SC, Jessen C, et al. Native IYG: Improving Psychosocial Protective Factors for HIV/STI and Teen Pregnancy Prevention among Youth in American Indian/ Alaska Native Communities. Journal of Applied Research on Children 2017; 8: 1-29.

111. Morrell HER, Cohen LM, Bacchi D, et al. Predictors of smoking and smokeless tobacco use in college students: a preliminary study using Web-based survey methodology. Journal of American College Health 2005; 54: 108-115. DOI: 10.3200/jach.54.2.108-115.

112. Worrall-Carter L, Daws K, Rahman MA, et al. Exploring Aboriginal patients' experiences of cardiac care at a major metropolitan hospital in Melbourne. Australian Health Review 2016; 40: 696-704. DOI: 10.1071/AH15175.

113. Rice ES, Haynes E, Royce P, et al. Social media and digital technology use among Indigenous young people in Australia: a literature review. International Journal for Equity in Health 2016; 15: 1-16. DOI: 10.1186/s12939-016-0366-0.

114. Adebajo A, Shikoh S, Kumar K, et al. Ethnic minority musculoskeletal health: Improving health literacy. Rheumatology 2018; 57: 201-203. DOI: 10.1093/rheumatology/kex006.

115. Bo X. Experimenting on the impact of learning methods and information presentation channels on older adults' e-health literacy. Journal of the American Society for Information Science & Technology 2011; 62: 1797-1807. DOI: 10.1002/asi.21575.

116. Laragy C, David C and Moran N. A framework for providing information in individualised funding programmes. Qualitative Social Work 2016; 15: 190-208. DOI: 10.1177/1473325015589402.

117. Ehrlich C, Kendall E, Parekh S, et al. The impact of culturally responsive self-management interventions on health outcomes for minority populations: A systematic review. Chronic Illness 2016; 12: 41-57. DOI: 10.1177/1742395315587764.

118. Jones K, Keeler N, Morris C, et al. Factors Relating to Access to Dental Care for Indigenous South Australians. Journal of Health Care for the Poor & Underserved 2016; 27: 148-160. DOI: 10.1353/hpu.2016.0042.

119. Hayes L, Bhochhibhoya A, Cheney M, et al. An Evaluation of the Effects of Formal Nutrition Education on Online Nutrition Information Retrieval among College Students. Journal of Consumer Health on the Internet 2016; 20: 139-155. DOI: 10.1080/15398285.2016.1198878.

120. Arora S, Kurji AK, Tennant MT, et al. Dismantling sociocultural barriers to eye care with tele-ophthalmology: lessons from an Alberta Cree community. Clinical & Investigative Medicine 2013; 36: E57-E57.

121. Wichmann F, Pischke CR, Jürgens D, et al. Requirements for (web-based) physical activity interventions targeting adults above the age of 65 years - qualitative results regarding acceptance and needs of participants and non-participants. BMC Public Health 2020; 20: 907-907. DOI: 10.1186/s12889-020-08927-8.

122. Lassetter JH, Clark L, Morgan SE, et al. Health Literacy and Obesity Among Native Hawaiian and Pacific Islanders in the United States. Public Health Nursing 2015; 32: 15-23. DOI: 10.1111/phn.12155.

123. Hildebrand DA, McCarthy P, Tipton D, et al. Innovative Use of Influential Prenatal Counseling May Improve Breastfeeding Initiation Rates Among WIC Participants. Journal of Nutrition Education & Behavior 2014; 46: 458-466. DOI: 10.1016/j.jneb.2014.05.005.

124. Whitacre B and Brooks L. Do broadband adoption rates impact a community's health? Behaviour & Information Technology 2014; 33: 767-779. DOI: 10.1080/0144929X.2013.830334.

125. Bottorff JL, Struik LL, Bissell LJL, et al. A social media approach to inform youth about breast cancer and smoking: An exploratory descriptive study. Collegian 2014; 21: 159-168. DOI: 10.1016/j.colegn.2014.04.002.

126. Lee YJ and Ha S. Consumer Use of the Internet for Health Management. Journal of Consumer Health on the Internet 2016; 20: 1-18. DOI: 10.1080/15398285.2015.1127114.

127. Morey OT. Digital disparities: the persistent digital divide as related to health information access on the Internet. Journal of Consumer Health on the Internet 2007; 11: 23-41.

128. MacDonald C, Browne J, Delbridge R, et al. You wouldn't eat 16 teaspoons of sugar – So why drink it? Community response to the Aboriginal Rethink Sugary Drink advertisement. Obesity Research & Clinical Practice 2019; 13: 61-62. DOI: 10.1016/j.orcp.2016.10.087.

129. Improving asthma health literacy. Kai Tiaki Nursing New Zealand 2015; 21: 11-11.

130. Morrow M. INDIGENOUS AND POPULATION HEALTH. Journal of Pharmacy Practice & Research 2013; 43: 151-152.

131. Morrow M. INDIGENOUS AND POPULATION HEALTH. Journal of Pharmacy Practice & Research 2013; 43: 151-152.

132. Palmer SC, Gray H, Huria T, et al. Reported Māori consumer experiences of health systems and programs in qualitative research: a systematic review with meta-synthesis. International Journal for Equity in Health 2019; 18: N.PAG-N.PAG. DOI: 10.1186/s12939-019-1057-4.

133. Cawthon C, Mion LC, Willens DE, et al. Implementing Routine Health Literacy Assessment in Hospital and Primary Care Patients. Joint Commission Journal on Quality & Patient Safety 2014; 40: 68-76. DOI: 10.1016/s1553-7250(14)40008-4.

134. Tsai H-M, Cheng C-Y, Chang S-C, et al. Health Literacy and Health-Promoting Behaviors among Multiethnic Groups of Women in Taiwan. JOGNN: Journal of Obstetric, Gynecologic & Neonatal Nursing 2014; 43: 117-129. DOI: 10.1111/1552-6909.12269.

135. Estacio EV. Health literacy and community empowerment: It is more than just reading, writing and counting. Journal of Health Psychology 2013; 18: 1056-1068. DOI: 10.1177/1359105312470126.

136. Gould GS, McEwen A, Watters T, et al. Should anti-tobacco media messages be culturally targeted for Indigenous populations? A systematic review and narrative synthesis. Tobacco Control 2013; 22: 1-10. DOI: 10.1136/tobaccocontrol-2012-050436.

137. Latycheva O, Chera R, Hampson C, et al. Engaging First Nation and Inuit communities in asthma management and control: Assessing cultural appropriateness of educational resources. Rural & Remote Health 2013; 13: 1-11.

138. Latycheva O, Chera R, Hampson C, et al. Engaging First Nation and Inuit communities in asthma management and control: Assessing cultural appropriateness of educational resources. Rural & Remote Health 2013; 13: 1-11.

139. Lebow M. Explore Arctic Health. Journal of Consumer Health on the Internet 2014; 18: 187-192. DOI: 10.1080/15398285.2014.902278.

140. Spicer P. Ask the expert. Zero to Three 2009; 29: 58-58.

141. Burke J. 'Ups' and 'downs' of diabetes care. Registered Nurse Journal 2008; 20: 11-11.

142. White WL, Evans AC, Lamb R, et al. Addiction Recovery Communities as Indigenous Cultures: Implications for Professional and Scientific Collaborations. Alcoholism Treatment Quarterly 2013; 31: 121-128. DOI: 10.1080/07347324.2013.746591.

143. Supplee LH, Harwood RL, Geyelin Margie N, et al. New Opportunities and Directions in Home Visiting Research and Evaluation. Zero to Three 2013; 33: 45-50.

144. Supplee LH, Harwood RL, Geyelin Margie N, et al. New Opportunities and Directions in Home Visiting Research and Evaluation. Zero to Three 2013; 33: 45-50.

145. Hom JM, Lee JY, Divaris K, et al. Oral health literacy and knowledge among patients who are pregnant for the first time. Journal of the American Dental Association (JADA) 2012; 143: 972-980. DOI: 10.14219/jada.archive.2012.0322.

146. Divaris K, Lee JY, Baker AD, et al. Caregivers' oral health literacy and their young children's oral health-related quality-of-life. Acta Odontologica Scandinavica 2012; 70: 390-397. DOI: 10.3109/00016357.2011.629627.

147. Henderson JA, Chubak J, O'Connell J, et al. Design of a randomized controlled trial of a web-based intervention to reduce cardiovascular disease risk factors among remote reservation-dwelling American Indian adults with type 2 diabetes. Journal of Primary Prevention 2012; 33: 209-222. DOI: 10.1007/s10935-012-0276-x.

148. Cortese J and Lustria MLA. Can tailoring increase elaboration of health messages delivered via an adaptive educational site on adolescent sexual health and decision making? Journal of the American Society for Information Science & Technology 2012; 63: 1567-1580. DOI: 10.1002/asi.22700.

149. Brega AG, Ang A, Vega W, et al. Mechanisms underlying the relationship between health literacy and glycemic control in American Indians and Alaska Natives. Patient Education & Counseling 2012; 88: 61-68.

150. Samuel KA, Ribisl KM and Williams RS. Internet cigarette sales and Native American sovereignty: Political and public health contexts. Journal of Public Health Policy 2012; 33: 173-187. DOI: 10.1057/jphp.2012.4.

151. Kagie R, Lin S-Y, Hussain MA, et al. A Pragmatic Review to Assist Planning and Practice in Delivering Nutrition Education to Indigenous Youth. Nutrients 2019; 11: 510-510. DOI: 10.3390/nu11030510.

152. Kaphingst KA, Goodman M, Pyke O, et al. Relationship Between Self-Reported Racial Composition of High School and Health Literacy Among Community Health Center Patients. Health Education & Behavior 2012; 39: 35-44. DOI: 10.1177/1090198111406538.

153. Cudney S and Weinert C. An online approach to providing chronic illness self-management information. CIN: Computers, Informatics, Nursing 2012; 30: 110-117. DOI: 10.1097/NCN.0b013e31822b899a.

154. Shaw RJ and Ferranti J. Patient-provider internet portals -- patient outcomes and use. CIN: Computers, Informatics, Nursing 2011; 29: 714-720. DOI: 10.1097/NCN.0b013e318224b597.

155. Bakker JP, O'Keeffe KM, Neill AM, et al. Ethnic Disparities in CPAP Adherence in New Zealand: Effects of Socioeconomic Status, Health Literacy and Self-Efficacy. Sleep 2011; 34: 1595-1603.

156. Sentell T, Baker KK, Onaka A, et al. Low health literacy and poor health status in Asian Americans and Pacific Islanders in Hawai'i. Journal of Health Communication 2011; 16: 279-294. DOI: 10.1080/10810730.2011.604390.

157. Okamoto SK, Helm S, Delp JA, et al. A community stakeholder analysis of drug resistance strategies of rural native Hawaiian youth. Journal of Primary Prevention 2011; 32: 185-193. DOI: 10.1007/s10935-011-0247-7.

158. Rushing SC, Stephens D, Rushing SC, et al. Use of media technologies by Native American teens and young adults in the Pacific Northwest: exploring their utility for designing culturally appropriate technology-based health interventions. Journal of Primary Prevention 2011; 32: 135-145. DOI: 10.1007/s10935-011-0242-z.

159. Vass A, Mitchell A, Dhurrkay Y, et al. Health literacy and Australian Indigenous peoples: an analysis of the role of language and worldview. Health Promotion Journal of Australia 2011; 22: 33-37.

160. Jamieson LM, Divaris K, Parker EJ, et al. Oral health literacy comparisons between Indigenous Australians and American Indians. Community Dental Health 2013; 30: 52-57. DOI: 10.1922/CDH 3025Jamieson06.

161. Lebow M. Explore Arctic Health. Journal of Consumer Health on the Internet 2012; 16: 187-192. DOI: 10.1080/15398285.2014.902278.

162. Christian-Kopp S, Sinha M, Chen AC-C, et al. Social Media Use Among Adolescent Patients Visiting an Inner City Pediatric Emergency Department. Journal of Consumer Health on the Internet 2012; 16: 147-161. DOI: 10.1080/15398285.2012.673463.

163. Johnson VBB and Lorig K. The Internet Diabetes Self-Management Workshop for American Indians and Alaska Natives. Health Promotion Practice 2011; 12: 261-270. DOI: 10.1177/1524839909335178.

164. Lee JY, Divaris K, Baker AD, et al. Oral health literacy levels among a low-income WIC population. Journal of Public Health Dentistry 2011; 71: 152-160. DOI: 10.1111/j.1752-7325.2011.00244.x.

165. Haverkamp D, Perdue DG, Espey D, et al. A survey of Indian health service and tribal health providers' colorectal cancer screening knowledge, perceptions, and practices. Journal of Health Care for the Poor & Underserved 2011; 22: 243-257.

166. Rey GN, Mora-Ríos J, Sainz MT, et al. An international perspective: Constructing intervention strategies for families in Mexico. Drugs: Education, Prevention & Policy 2010; 17: 193-202. DOI: 10.3109/09687637.2010.514787.

167. Vann WF, Jr., Lee JY, Baker D, et al. Oral health literacy among female caregivers: impact on oral health outcomes in early childhood. Journal of Dental Research 2010; 89: 1395-1400. DOI: 10.1177/0022034510379601.

168. Barnes SJ, Hamasu C and Nail-Chiwetalu B. A Place for Sharing-Outreach Connections: Native Health Information. Journal of Hospital Librarianship 2010; 10: 387-394. DOI: 10.1080/15323269.2010.514560.

169. Burhansstipanov L, Krebs LU, Seals BF, et al. Native American breast cancer survivors' physical conditions and quality of life. Cancer (0008543X) 2010; 116: 1560-1571. DOI: 10.1002/cncr.24924.

170. Taualii M, Bush N, Bowen DJ, et al. Adaptation of a smoking cessation and prevention website for urban American Indian/Alaska Native youth. Journal of Cancer Education 2010; 25: 23-31. DOI: 10.1007/s13187-009-0004-2.

171. Nagel TM, Thompson C, Robinson G, et al. Two-way approaches to Indigenous mental health literacy. Australian Journal of Primary Health 2009; 15: 50-55. DOI: 10.1071/py08052.

172. Steyn NP, Lambert EV and Tabana H. Conference on 'Multidisciplinary approaches to nutritional problems'. Symposium on 'Diabetes and health'. Nutrition interventions for the prevention of type 2 diabetes. Proceedings of the Nutrition Society 2009; 68: 55-70. DOI: 10.1017/S0029665108008823.

173. Willmon-Haque S and BigFoot DS. Violence and the effects of trauma on American Indian and Alaska native populations. Journal of Emotional Abuse 2008; 8: 51-66. DOI: 10.1080/10926790801982410.

174. Hoberecht T and Miller-Cribbs J. Some Health Literacy Aspects of a Photovoice Project. Journal of Consumer Health on the Internet 2011; 15: 389-395. DOI: 10.1080/15398285.2011.623590.

175. Choi CS, Haynes S, Konsella L, et al. The Quick Health Data Online: The Ultimate Interactive Database on Recent Trends in Women's Health. Journal of Women's Health (15409996) 2007; 16: 941-958. DOI: 10.1089/jwh.2007.DH01.

176. Friedman DB, Hoffman-Goetz L, Friedman DB, et al. Assessing cultural sensitivity of breast cancer information for older Aboriginal women. Journal of Cancer Education 2007; 22: 112-118. DOI: 10.1007/bf03174359.

177. Brown SL, Teufel JA and Birch DA. Early adolescents perceptions of health and health literacy. Journal of School Health 2007; 77: 7-15. DOI: 10.1111/j.1746-1561.2007.00156.x.

178. Swartz LH, Noell JW, Schroeder SW, et al. A randomised control study of a fully automated internet based smoking cessation programme. Tobacco Control 2006; 15: 7-12. DOI: 10.1136/tc.2003.006189.

179. Roubidoux MA, Hilmes M, Abate S, et al. Development of computer games to teach breast cancer screening to Native American patients and their healthcare providers. Journal of Women's Imaging 2005; 7: 77-95. DOI: 10.1097/01.jwi.0000168675.22467.f6.

180. Koch T, Mann S, Kralik D, et al. Focus. Reflection: look, think and act cycles in participatory action research. Journal of Research in Nursing 2005; 10: 261-278.

181. Austin PON. Promoting Health Literacy Among Rural Native Hawaiians and Pacific Islanders with Hypertension. Promoting Health Literacy Among Rural Native Hawaiians & Pacific Islanders with Hypertension 2017: 1-1.

182. Power JM, Braun KL and Bersamin A. Exploring the Potential for Technology-Based Nutrition Education Among WIC Recipients in Remote Alaska Native Communities. Journal of Nutrition Education & Behavior 2017; 49: S186-S191.e181. DOI: 10.1016/j.jneb.2016.11.003.

183. Report on the Maternal and Mental Health Workshop. Midwifery News 2009: 22-23.

184. Stillwater B. State of Alaska 2008 recommendations for the management of diabetes type 2. Alaska Nurse 2008; 58: 8-9.

185. Marquand B. Minority women and intimate partner violence. Minority Nurse 2010: 14-18.

186. Safratowich M, Markland MJ and Rieke JL. Discover health services near you! The North Dakota story: part II. Journal of Consumer Health on the Internet 2009; 13: 223-236.

187. Ottosen T. Webhealth topics. Veterans health information: a webliography. Journal of Consumer Health on the Internet 2009; 13: 53-65.

188. Marquand B. The color of hope. Minority Nurse 2008: 20-24.

189. Rieke JL. Linking Native Americans to quality health information on the Internet: a tribal college library collaboration. Journal of Consumer Health on the Internet 2005; 9: 27-42.

190. Kearney G. Editorial. Australian Nursing Journal 2008; 16: 1-1.

191. Povey J, Mills PPJR, Dingwall KM, et al. Acceptability of Mental Health Apps for Aboriginal and Torres Strait Islander Australians: A Qualitative Study. Journal of Medical Internet Research 2016; 18: 24-24. DOI: 10.2196/jmir.5314.

192. Brusse C, Gardner K, McAullay D, et al. Social media and mobile apps for health promotion in Australian Indigenous populations: scoping review. Journal of Medical Internet Research 2014; 16: e280-e280. DOI: 10.2196/jmir.3614.

193. Reti SR, Feldman HJ and Safran C. Online access and literacy in Maori New Zealanders with diabetes. Journal of Primary Health Care 2011; 3: 190-191.

194. In Short. Australian Occupational Therapy Journal 2013: 23-23.

195. In Short. Australian Occupational Therapy Journal 2013: 23-23.

196. Sweet MA. Social media: new links for Indigenous health. Medical Journal of Australia 2013; 199: 18-18.

197. Norton M. Half of Kiwis have low health literacy. Pharmacy Today (11701927) 2014: 15-15.

198. Lassetter JH, Clark L, Brown LB, et al. Pacific Islanders' Health Literacy, Diet/Activity Recall, and Self-Efficacy. Communicating Nursing Research 2013; 46: 373-373.

199. Rubenfire M. [Commentary on] Prediction of coronary heart disease in a population with high prevalence of diabetes and albuminuria: the Strong Heart Study. ACC Cardiosource Review Journal 2006; 15: 66-67.

200. Parker EJ, Misan G, Chong A, et al. An oral health literacy intervention for Indigenous adults in a rural setting in Australia. BMC Public Health 2012; 12: 461-461. DOI: 10.1186/1471-2458-12-461.

201. Gleeson S. For the toolbox. Update Newsletter of the Australian Health Promotion Association 2011: 5-5.

202. Nursing news. Manitoba RN Journal 2009; 34: 8-9.

203. Chwedyk P. Vital signs. News grand rounds. Minority Nurse 2007: 12-13.

204. Spooner G. Web of knowledge: Internet highlights from the college's e-bulletin. nursingaust 2006; 7: 22-23.

205. Kay-Lambkin FJ, White A, Baker AL, et al. Assessment of function and clinical utility of alcohol and other drug web sites: an observational, qualitative study. BMC Public Health 2011; 11: 277-277. DOI: 10.1186/1471-2458-11-277.

206. Spooner G. Web of knowledge: Internet highlights from the college's e-bulletin. nursingaust 2005; 6: 26-27.

207. Si D, Bailie R, Wang Z, et al. Comparison of diabetes management in five countries for general and indigenous populations: an internet-based review. BMC Health Services Research 2010; 10: 169-169. DOI: 10.1186/1472-6963-10-169.

208. Parker EJ and Jamieson LM. Associations between Indigenous Australian oral health literacy and self-reported oral health outcomes. BMC Oral Health 2010; 10: 8p-8p. DOI: 10.1186/1472-6831-10-3.

209. On-line resources. Zero to Three 2007; 27: 63-63.

210. Winning programs prevent addiction, disease and falls. Aging Today 2006; 27: 11-12.

211. Baldridge D and Benson WF. Indian country faces abuse. Aging Today 2005; 26: 7-8.

212. Health information made to order. InMotion 2007; 17: 24-24.

# PsychoInfo-Ovid 2022.03.08

## Notes about this database search

## Actual search terms for PsychoInfo-Ovid

APA PsycInfo <1806 to February Week 4 2022>

1 "Racial and Ethnic Groups"/ or Minority Groups/ or Oceanic Ancestry Group.mp. 29873

2 Alaska Natives/ or American Indians/ or Indigenous Populations/ or Inuit/ 14106

3 Indigenous Peoples.mp. 1416

4 (Aboriginal or First Nations or Torres Strait or Maori or Sami or Ainu).tw. 6320

5 American Native Continental ancestry group.mp. 66

6 1 or 2 or 3 or 4 or 5 45956

7 Internet/ or Social Media/ or Blog/ or Online Social Networks/ or Computer Mediated Communication/ 50593

8 email.tw. 4684

9 Internet Usage/ or Online Social Networks/ 11338

10 Digital Interventions/ 810

11 Social Networks/ or Websites/ 18999

12 (smartphone or "mobile application" or App or e-health or ehealth or internet or email or Facebook or SnapChat or Instagram or Twitter or TicToc or Pinterest or youtube).tw. 70228

13 7 or 8 or 9 or 10 or 11 or 12 107200

14 Health Education/ or "Information and Communication Technology"/ or Health Information Technology/ or Health Information/ or Information Seeking/ or Health Promotion/ or Health Literacy/ or sex education.mp. [mp=title, abstract, heading word, table of contents, key concepts, original title, tests & measures, mesh word] 65038

15 healthy people programs.mp. 205

16 weight reduction programs.tw. 67

17 "Substance Use Prevention"/ or Tobacco Smoking/ or Smoking Cessation/ 43296

18 "Tobacco Use Disorder"/ or "Substance Use Disorder"/ or Electronic Cigarettes/ 11734

19 Drug Abuse/ or Addiction/ or Relapse Prevention/ or Drug Addiction/ or alcoholism/ 97756

20 Psychological Stress/ or Stress Management/ or Stress/ 82461

21 "Quality of Life"/ or Well being/ or Self Care/ 95671

22 Chronic Obstructive Pulmonary Disease/ or Chronic Illness/ or neoplasms/ or pulmonary emphysema/ 53731

23 Type 2 Diabetes/ or Diabetes/ or Diabetes Mellitus/ 18517

24 Cardiovascular Disorders/ or Heart Disorders/ 20470

25 14 or 15 or 16 or 17 or 18 or 19 or 20 or 21 or 22 or 23 or 24 443607

26 6 and 13 and 25 155

27 limit 26 to (english language and yr="2005 -Current") 146

## Link to saved search for PsychoInfo-Ovid

<https://simsrad.net.ocs.mq.edu.au/login?url=http://ovidsp.ovid.com/ovidweb.cgi?T=JS&NEWS=N&PAGE=main&SHAREDSEARCHID=1OlDpHBqfox3WZ3U3L0kittRHKB0ZPKknxUfrP9DE4zdpjNn0caNt6zidevzg6LgM>

## References found by PsychInfo 2022.03.08

1. Bauer JE, Englert JJ, Michalek AM, et al. American Indian Cancer Survivors: Exploring Social Network Topology and Perceived Social Supports. *Journal of Cancer Education* 2005; 20: 23-27.

2. Koch T, Mann S, Kralik D, et al. Reflection: Look, think and act cycles in participatory action research. *Journal of Research in Nursing* 2005; 10: 261-278.

3. Nguyen KD, Hara B and Chlebowski RT. Utility of Two Cancer Organization Websites for a Multiethnic, Public Hospital Oncology Population: Comparative Cross-Sectional Survey. *Journal of Medical Internet Research* 2005; 7: No Pagination Specified.

4. Talosig-Garcia M and Davis SW. Information-seeking behavior of minority breast cancer patients: An exploratory study. *Journal of Health Communication* 2005: 53-64.

5. Drabble L, Keatley JG and Marcelle G. Media Strategies for Advancing Health in Lesbian, Gay, Bisexual, and Transgender Communities. *The handbook of lesbian, gay, bisexual, and transgender public health: A practitioner's guide to service*. Binghamton: Harrington Park Press, 2006, pp.335-352.

6. van der Velden M. Invisability and the ethics of digitalization: Designing so as not to hurt others. *Information technology ethics: Cultural perspectives*. Hershey, PA: Idea Group Reference/IGI Global; US, 2007, pp.81-93.

7. Campbell RJ. Meeting seniors' information needs: Using computer technology. *Home Health Care Management & Practice* 2008; 20: 328-335.

8. Graham AL and Papandonatos GD. Reliability of internet- versus telephone-administered questionnaires in a diverse sample of smokers. *Journal of Medical Internet Research* 2008; 10: 90-103.

9. Im E-O, Lim H-J, Clark M, et al. African American cancer patients' pain experience. *Cancer Nursing* 2008; 31: 38-46.

10. Jernigan VJ. The native American diabetes self-management program. *Dissertation Abstracts International: Section B: The Sciences and Engineering* 2008; 68: 5170.

11. Kumar S and Browne MA. Usefulness of the construct of social network to explain mental health service utilization by the Maori population in New Zealand. *Transcultural Psychiatry* 2008; 45: 439-454.

12. Pinto RM, Melendez RM and Spector AY. Male-to-female transgender individuals building social support and capital from within a gender-focused network. *Journal of Gay & Lesbian Social Services: Issues in Practice, Policy & Research* 2008; 20: 203-220.

13. Campbell RJ. Internet-based health information seeking among low-income, minority seniors living in urban residential centers. *Home Health Care Management & Practice* 2009; 21: 195-202.

14. Rafnsson SB and Bhopal RS. Large-scale epidemiological data on cardiovascular diseases and diabetes in migrant and ethnic minority groups in Europe. *European Journal of Public Health* 2009; 19: 484-491.

15. Cutilli CC. Seeking health information: What sources do your patients use? *Orthopaedic Nursing* 2010; 29: 214-219.

16. Macnab A, Radziminski N, Budden H, et al. Brighter smiles Africa-Translation of a Canadian community-based health-promoting school program to Uganda. *Education for Health: Change in Learning & Practice* 2010; 23: 1-8.

17. Oh A, Shaikh A, Waters E, et al. Health disparities in awareness of physical activity and cancer prevention: Findings from the National Cancer Institute's 2007 Health Information National Trends Survey (HINTS). *Journal of Health Communication* 2010; 15: 60-77.

18. Taualii M, Bush N, Bowen DJ, et al. Adaptation of a smoking cessation and prevention website for urban American Indian/Alaska Native youth. *Journal of Cancer Education* 2010; 25: 23-31.

19. Gibbons M, Fleisher L, Slamon RE, et al. Exploring the potential of Web 2.0 to address health disparities. *Journal of Health Communication* 2011; 16: 77-89.

20. Kontos EZ, Emmons KM, Puleo E, et al. Determinants and beliefs of health information mavens among a lower-socioeconomic position and minority population. *Social Science & Medicine* 2011; 73: 22-32.

21. Leech TGJ and Littlefield M. Social support and resilience in the aftermath of sexual assault: Suggestions across life course, gender, and racial groups. *Surviving sexual violence: A guide to recovery and empowerment*. Lanham, MD: Rowman & Littlefield; US, 2011, pp.296-317.

22. Lehavot K and Simoni JM. Victimization, smoking, and chronic physical health problems among sexual minority women. *Annals of Behavioral Medicine* 2011; 42: 269-276.

23. Paquette DM, Bryant J and De Wit J. Use of respondent-driven sampling to enhance understanding of injecting networks: A study of people who inject drugs in Sydney, Australia. *International Journal of Drug Policy* 2011; 22: 267-273.

24. Radoll P. The primary determinants of Indigenous household information and communication technology adoption: Remote, rural and urban. *Australasian Psychiatry* 2011; 19: S49-S52.

25. Ralph LJ, Berglas NF, Schwartz SL, et al. Finding teens in TheirSpace: Using social networking sites to connect youth to sexual health services. *Sexuality Research & Social Policy: A Journal of the NSRC* 2011; 8: 38-49.

26. Rhodes SD, Hergenrather KC, Vissman AT, et al. Boys must be men, and men must have sex with women: A qualitative CBPR study to explore sexual risk among African American, Latino, and White gay men and MSM. *American Journal of Men's Health* 2011; 5: 140-151.

27. Sussman S, Lisha N and Griffiths M. Prevalence of the addictions: A problem of the majority or the minority? *Evaluation & the Health Professions* 2011; 34: 3-56.

28. Bjorkman M and Malterud K. Lesbian women coping with challenges of minority stress: A qualitative study. *Scandinavian Journal of Public Health* 2012; 40: 239-244.

29. Bowen DJ, Henderson PN, Harvill J, et al. Short-term effects of a smoking prevention Website in American Indian youth. *Journal of Medical Internet Research* 2012; 14: 185-192.

30. Burhansstipanov L, Clark RE, Watanabe-Galloway S, et al. Online evaluation programs: Benefits and limitations. *Journal of Cancer Education* 2012; 27: S24-S31.

31. Geana MV, Daley CM, Nazir N, et al. Use of online health information resources by American Indians and Alaska Natives. *Journal of Health Communication* 2012; 17: 820-835.

32. Geana MV, Greiner K, Cully A, et al. Improving health promotion to American Indians in the Midwest United States: Preferred sources of health information and its use for the medical encounter. *Journal of Community Health: The Publication for Health Promotion and Disease Prevention* 2012; 37: 1253-1263.

33. Gray JS and Carter PM. Growing our own: Building a Native research team. *Journal of Psychoactive Drugs* 2012; 44: 160-165.

34. Im E-O, Chang SJ, Chee W, et al. Attitudes of women in midlife to web-based interventions for promoting physical activity. *Journal of Telemedicine and Telecare* 2012; 18: 419-422.

35. Kelley R, Hannans A, Kreps G, et al. The Community Liaison Program: A health education pilot program to increase minority awareness of HIV and acceptance of HIV vaccine trials. *Health Education Research* 2012; 27: 746-754.

36. Mesch G, Mano R and Tsamir J. Minority status and health information search: A test of the social diversification hypothesis. *Social Science & Medicine* 2012; 75: 854-858.

37. Rushing SC and Stephens D. Tribal recommendations for redesigning culturally appropriate technology-based sexual health interventions targeting native youth in the Pacific Northwest. *American Indian and Alaska Native Mental Health Research* 2012; 19: 76-101.

38. Bockting WO, Miner MH, Romine RE, et al. Stigma, mental health, and resilience in an online sample of the US transgender population. *American Journal of Public Health* 2013; 103: 943-951.

39. DeBlaere C and Bertsch KN. Perceived sexist events and psychological distress of sexual minority women of color: The moderating role of womanism. *Psychology of Women Quarterly* 2013; 37: 167-178.

40. Filippi MK, McCloskey C, Williams C, et al. Perceptions, barriers, and suggestions for creation of a tobacco and health website among American Indian/Alaska Native college students. *Journal of Community Health: The Publication for Health Promotion and Disease Prevention* 2013; 38: 486-491.

41. Gould GS, McEwen A, Watters T, et al. Should anti-tobacco media messages be culturally targeted for Indigenous populations? A systematic review and narrative synthesis. *Tobacco Control: An International Journal* 2013; 22: 1-10.

42. Johns MM, Pingel ES, Youatt EJ, et al. LGBT community, social network characteristics, and smoking behaviors in young sexual minority women. *American Journal of Community Psychology* 2013; 52: 141-154.

43. Nicolas-Rocca TS and Parrish JL, Jr. Capturing and conveying Chamorro cultural knowledge using social media. *International Journal of Knowledge Management* 2013; 9: 1-18.

44. Sulfridge RM. An ethnographic analysis of adolescent sexual minority website usage: Exploring notions of information seeking and sexual identity development. *Dissertation Abstracts International Section A: Humanities and Social Sciences* 2013; 73: No Pagination Specified.

45. Adams-Bass VN, Stevenson HC and Kotzin DS. Measuring the meaning of Black media stereotypes and their relationship to the racial identity, Black history knowledge, and racial socialization of African American youth. *Journal of Black Studies* 2014; 45: 367-395.

46. Anderson KJ. Tobacco use and cessation: What matters to southeast alaska native young adults? *Dissertation Abstracts International: Section B: The Sciences and Engineering* 2014; 75: No Pagination Specified.

47. Bull S, Walker T and Levine D. Communities and technology: Enhancements in HIV-prevention research and practice among adolescents and young adults. *Innovation in HIV prevention research and practice through community engagement*. New York, NY: Springer Science + Business Media; US, 2014, pp.183-214.

48. Cueva M, Cueva K, Dignan M, et al. Evaluating arts-based cancer education using an internet survey among Alaska Community Health Workers. *Journal of Cancer Education* 2014; 29: 529-535.

49. Lee YJ. Online health information seeking behaviors of Hispanics in New York city. *Dissertation Abstracts International: Section B: The Sciences and Engineering* 2014; 74: No Pagination Specified.

50. Mitchell KJ, Ybarra ML, Korchmaros JD, et al. Accessing sexual health information online: Use, motivations and consequences for youth with different sexual orientations. *Health Education Research* 2014; 29: 147-157.

51. Wong CF, Schrager SM, Holloway IW, et al. Minority stress experiences and psychological well-being: The impact of support from and connection to social networks within the Los Angeles House and Ball communities. *Prevention Science* 2014; 15: 44-55.

52. Chambers RA, Rosenstock S, Neault N, et al. A home-visiting diabetes prevention and management program for American Indian youth: The together on diabetes trial. *The Diabetes Educator* 2015; 41: 729-747.

53. Craig SL, McInroy LB, McCready LT, et al. Connecting without fear: Clinical implications of the consumption of information and communication technologies by sexual minority youth and young adults. *Clinical Social Work Journal* 2015; 43: 159-168.

54. Dingwall KM, Puszka S, Sweet M, et al. "Like drawing into sand": Acceptability, feasibility, and appropriateness of a new e-mental health resource for service providers working with Aboriginal and Torres Strait Islander people. *Australian Psychologist* 2015; 50: 60-69.

55. Ham O-K, Kang Y, Teng H, et al. Consistency and accuracy of multiple pain scales measured in cancer patients from multiple ethnic groups. *Cancer Nursing* 2015; 38: 305-311.

56. Hildebrand J, Burns S, Zhao Y, et al. Potential and challenges in collecting social and behavioral data on adolescent alcohol norms: Comparing respondent-driven sampling and Web-based respondent-driven sampling. *Journal of Medical Internet Research* 2015; 17: 1-15.

57. Huang Y-M, Chen M-Y and Mo S-S. How do we inspire people to contact aboriginal culture with Web2.0 technology? *Computers & Education* 2015; 86: 71-83.

58. Mollenhorst G, Edling C and Rydgren J. Psychological well-being and brokerage in friendship networks of young Swedes. *Social Indicators Research* 2015; 123: 897-917.

59. Schwinn TM, Thom B, Schinke SP, et al. Preventing drug use among sexual-minority youths: Findings from a tailored, web-based intervention. *Journal of Adolescent Health* 2015; 56: 571-573.

60. Song H, Cramer EM and McRoy S. Information gathering and technology use among low-income minority men at risk for prostate cancer. *American Journal of Men's Health* 2015; 9: 235-246.

61. Sweet M, Geia L, Dudgeon P, et al. #IHMayDay: Tweeting for empowerment and social and emotional wellbeing. *Australasian Psychiatry* 2015; 23: 636-640.

62. Trotter RT, II, Laurila K, Alberts D, et al. A diagnostic evaluation model for complex research partnerships with community engagement: The partnership for Native American Cancer Prevention (NACP) model. *Evaluation and Program Planning* 2015; 48: 10-20.

63. Zeraiq L, Nielsen D and Sodemann M. Attitudes towards human papillomavirus vaccination among Arab ethnic minority in Denmark: A qualitative study. *Scandinavian Journal of Public Health* 2015; 43: 408-414.

64. Gibson A, Lee C and Crabb S. Representations of women on Australian breast cancer websites: Cultural 'inclusivity' and marginalisation. *Journal of Sociology* 2016; 52: 433-452.

65. Logie CH, Lacombe-Duncan A, MacKenzie RK, et al. Minority stress and safer sex practices among sexual minority women in Toronto, Canada: Results from a cross-sectional internet-based survey. *LGBT Health* 2016; 3: 407-415.

66. Nguyen TQ, Bandeen-Roche K, German D, et al. Negative treatment by family as a predictor of depressive symptoms, life satisfaction, suicidality, and tobacco/alcohol use in vietnamese sexual minority women. *LGBT Health* 2016; 3: 357-365.

67. Philip J, Ford T, Henry D, et al. Relationship of Social Network to Protective Factors in Suicide and Alcohol Use Disorder Intervention for Rural Yup'ik Alaska Native Youth. *Psychosocial Intervention* 2016; 25: 45-54.

68. Pokhrel P, Fagan P, Cassel K, et al. Social network characteristics, social support, and cigarette smoking among Asian/Pacific Islander young adults. *American Journal of Community Psychology* 2016; 57: 353-365.

69. Ramsey J. "Aho! all my relations": Native identity and its relationship to psychological well-being. *Dissertation Abstracts International: Section B: The Sciences and Engineering* 2016; 76: No Pagination Specified.

70. Bennett-Levy J, Singer J, DuBois S, et al. Translating e-mental health into practice: What are the barriers and enablers to e-mental health implementation by Aboriginal and Torres Strait Islander health professionals? *Journal of Medical Internet Research* 2017; 19: No Pagination Specified.

71. Dispenza F. Career and romantic partnership role salience between sexual minority men living with and without a chronic illness/disability (CID). *Psychology of Men & Masculinity* 2017; 18: 157-164.

72. Dispenza F, Varney M and Golubovic N. Counseling and psychological practices with sexual and gender minority persons living with chronic illnesses/disabilities (CID). *Psychology of Sexual Orientation and Gender Diversity* 2017; 4: 137-142.

73. Fleming JB and Burns MN. Online evaluative conditioning did not alter internalized homonegativity or self-esteem in gay men. *Journal of Clinical Psychology* 2017; 73: 1013-1026.

74. Geia L, Pearson L and Sweet M. Narratives of twitter as a platform for professional development, innovation, and advocacy. *Australian Psychologist* 2017; 52: 280-287.

75. Harding A. The quality of life of minority women in the United States with lupus: Assessment by an internet based survey. *Dissertation Abstracts International: Section B: The Sciences and Engineering* 2017; 77: No Pagination Specified.

76. Janca A, Lyons Z and Gaspar J. Here and Now Aboriginal Assessment (HANAA): A follow-up survey of users. *Australasian Psychiatry* 2017; 25: 288-289.

77. Kelley A, Bingham D, Brown E, et al. Assessing the impact of American Indian peer recovery support on substance use and health. *Journal of Groups in Addiction & Recovery* 2017; 12: 296-308.

78. Lee JH, Giovenco D and Operario D. Patterns of health information technology use according to sexual orientation among US adults aged 50 and older: Findings from a national representative sample-National Health Interview Survey 2013-2014. *Journal of Health Communication* 2017; 22: 666-671.

79. Luczak SE, Khoddam R, Yu S, et al. Review: Prevalence and co-occurrence of addictions in US ethnic/racial groups: Implications for genetic research. *The American Journal on Addictions* 2017; 26: 424-436.

80. Lumpkins CY, Mabachi N, Lee J, et al. A prescription for Internet access: Appealing to middle-aged and older racial and ethnic minorities through social network sites to combat colorectal cancer. *Health Communication* 2017; 32: 916-920.

81. Massey PM, Langellier BA, Sentell T, et al. Nativity and language preference as drivers of health information seeking: Examining differences and trends from a U.S. population-based survey. *Ethnicity & Health* 2017; 22: 596-609.

82. Nguyen AB, Robinson J, O'Brien EK, et al. Racial and ethnic differences in tobacco information seeking and information sources: Findings from the 2015 Health Information National Trends Survey. *Journal of Health Communication* 2017; 22: 743-752.

83. Stevens R, Gilliard-Matthews S, Dunaev J, et al. Social media use and sexual risk reduction behavior among minority youth: Seeking safe sex information. *Nursing Research* 2017; 66: 368-377.

84. Van Bewer V and Woodgate RL. Examining the correlates of current smoking among off-reserve First Nations, Metis and Inuit youth: Evidence from the 2012 Aboriginal Peoples Survey. *Addictive Behaviors* 2017; 69: 93-97.

85. Williams-Johnson LM. Preventive health education media and older worker health literacy. *Dissertation Abstracts International: Section B: The Sciences and Engineering* 2017; 77: No Pagination Specified.

86. Abbass-Dick J, Brolly M, Huizinga J, et al. Designing an ehealth breastfeeding resource with indigenous families using a participatory design. *Journal of Transcultural Nursing* 2018; 29: 480-488.

87. Bartikowski B, Laroche M, Jamal A, et al. The type-of-internet-access digital divide and the well-being of ethnic minority and majority consumers: A multi-country investigation. *Journal of Business Research* 2018; 82: 373-380.

88. Black KJ, Morse B, Tuitt N, et al. Beyond content: Cultural perspectives on using the internet to deliver a sexual health intervention to American Indian youth. *The Journal of Primary Prevention* 2018; 39: 59-70.

89. Burhansstipanov L, Krebs LU, Petereit D, et al. Reality versus grant application research "plans". *Health Promotion Practice* 2018; 19: 566-572.

90. Chard AN, Metheny NS, Sullivan PS, et al. Social stressors and intoxicated sex among an online sample of men who have sex with men (MSM) drawn from seven countries. *Substance Use & Misuse* 2018; 53: 42-50.

91. Conner TS, McFarlane KG, Choukri M, et al. The oxytocin receptor gene (OXTR) variant rs53576 is not related to emotional traits or states in young adults. *Frontiers in Psychology Vol 9 2018, ArtID 2548* 2018; 9.

92. Jang M, Johnson CM, D'Eramo-Melkus G, et al. Participation of racial and ethnic minorities in technology-based interventions to self-manage type 2 diabetes: A scoping review. *Journal of Transcultural Nursing* 2018; 29: 292-307.

93. Lu L, Xu D-D, Liu H-Z, et al. Internet addiction in Tibetan and Han Chinese middle school students: Prevalence, demographics and quality of life. *Psychiatry Research* 2018; 268: 131-136.

94. Manduley AE, Mertens AE, Plante I, et al. "The role of social media in sex education: Dispatches from queer, trans, and racialized communities": Erratum. *Feminism & Psychology* 2018; 28: 453-454.

95. Manduley AE, Mertens AE, Plante I, et al. The role of social media in sex education: Dispatches from queer, trans, and racialized communities. *Feminism & Psychology* 2018; 28: 152-170.

96. McPhail-Bell K, Appo N, Haymes A, et al. Deadly Choices empowering indigenous Australians through social networking sites. *Health Promotion International* 2018; 33: 770-780.

97. McQuoid J, Thrul J and Ling P. A geographically explicit ecological momentary assessment (GEMA) mixed method for understanding substance use. *Social Science & Medicine* 2018; 202: 89-98.

98. Park H, Rodgers S, McElroy JA, et al. Sexual and gender minority's social media user characteristics: Examining preferred health information. *Health Marketing Quarterly* 2018; 35: 1-17.

99. Tabb KM. Black sexual minorities coping with multiple minority stress. *Dissertation Abstracts International: Section B: The Sciences and Engineering* 2018; 78: No Pagination Specified.

100. Wong G, Glover M, McPherson M, et al. Boosting efficacy of nurse-led stop smoking interventions with a quit and win contest: Pilot study results. *Contemporary Nurse* 2018; 54: 395-408.

101. Dayyani I, Terkildsen Maindal H, Rowlands G, et al. A qualitative study about the experiences of ethnic minority pregnant women with gestational diabetes. *Scandinavian Journal of Caring Sciences* 2019; 33: 621-631.

102. Desrosiers A, Vine V and Kershaw T. "R U Mad?": Computerized text analysis of affect in social media relates to stress and substance use among ethnic minority emerging adult males. *Anxiety, Stress & Coping: An International Journal* 2019; 32: 109-123.

103. Dingwall KM, Nagel T, Hughes JT, et al. Wellbeing intervention for chronic kidney disease (WICKD): A randomised controlled trial study protocol. *BMC Psychology Vol 7 2019, ArtID 2* 2019; 7.

104. Dowsett M, Islam M, Ganora C, et al. Asking young aboriginal people who use illicit drugs about their healthcare preferences using audio-computer-assisted self-interviewing. *Drug and Alcohol Review* 2019; 38: 482-493.

105. Fernandez SB, Howard M, Hospital M, et al. Hispanic students' perceptions about HIV/STI testing and prevention: A mixed-methods study in a Hispanic-serving university. *Health Promotion Practice* 2019; 20: 742-750.

106. Gonzalez M, Sanders-Jackson A and Wright T. Web-based health information technology: Access among Latinos varies by subgroup affiliation. *Journal of Medical Internet Research Vol 21(4), 2019, ArtID e10389* 2019; 21.

107. Griffiths S, Murray SB, Mitchison D, et al. Relative strength of the associations of body fat, muscularity, height, and penis size dissatisfaction with psychological quality of life impairment among sexual minority men. *Psychology of Men & Masculinities* 2019; 20: 55-60.

108. Langford A and Loeb S. Perceived patient-provider communication quality and sociodemographic factors associated with watching health-related videos on YouTube:A cross-sectional analysis. *Journal of Medical Internet Research Vol 21(5), 2019, ArtID e13512* 2019; 21.

109. Levin-Zamir D and Bertschi I. Media health literacy, eHealth literacy and health behaviour across the lifespan: Current progress and future challenges. *International handbook of health literacy: Research, practice and policy across the life-span*. Bristol, United Kingdom: Policy Press; United Kingdom, 2019, pp.275-290.

110. McCrabb S, Twyman L, Palazzi K, et al. A cross sectional survey of Internet use among a highly socially disadvantaged population of tobacco smokers. *Addiction Science & Clinical Practice Vol 14 2019, ArtID 38* 2019; 14.

111. Ospina-Pinillos L, Davenport T, Mendoza Diaz A, et al. Using participatory design methodologies to co-design and culturally adapt the Spanish version of the Mental Health eClinic: Qualitative study. *Journal of Medical Internet Research Vol 21(8), 2019, ArtID e14127* 2019; 21.

112. Panza E. Minority stress and eating behavior among overweight and obese sexual minority women: An ecological momentary assessment study. *Dissertation Abstracts International: Section B: The Sciences and Engineering* 2019; 80: No Pagination Specified.

113. Park NS, Jang Y, Chiriboga DA, et al. Social network types, health, and well-being of older Asian Americans. *Aging & Mental Health* 2019; 23: 1569-1577.

114. Pyke J, Pyke S and Watuwa R. Social tourism and well-being in a first nation community. *Annals of Tourism Research* 2019; 77: 38-48.

115. Thomas DP, Davey ME, van der Sterren AE, et al. Social networks and quitting in a national cohort of Australian Aboriginal and Torres Strait Islander smokers. *Drug and Alcohol Review* 2019; 38: 82-91.

116. Tsai W, Zavala D and Gomez S. Using the Facebook advertisement platform to recruit Chinese, Korean, and Latinx cancer survivors for psychosocial research: Web-based survey study. *Journal of Medical Internet Research Vol 21(1), 2019, ArtID e11571* 2019; 21.

117. Verbiest ME, Corrigan C, Dalhousie S, et al. Using codesign to develop a culturally tailored, behavior change mHealth intervention for indigenous and other priority communities: A case study in New Zealand. *Translational Behavioral Medicine* 2019; 9: 720-736.

118. Alkazemi MF and Van Stee SK. Electronic direct-to-consumer advertising of pharmaceuticals: An assessment of textual and visual content of websites. *Health Education Research* 2020; 35: 134-151.

119. Bennett AJ. Exploring hegemonic masculinity in online interactions and behavior. *Dissertation Abstracts International Section A: Humanities and Social Sciences* 2020; 81: No Pagination Specified.

120. Escobedo P, Tsai K-Y, Majmundar A, et al. Do tobacco industry websites target content to specific demographic groups? *Drug and Alcohol Dependence Vol 208 2020, ArtID 107852* 2020; 208.

121. Guo M, Ganz O, Cruse B, et al. Keeping it fresh with hip-hop teens: Promising targeting strategies for delivering public health messages to hard-to-reach audiences. *Health Promotion Practice* 2020; 21: 61S-71S.

122. Manduley AE, Mertens AE, Plante I, et al. "The role of social media in sex education: Dispatches from queer, trans, and racialized communities": Corrigendum. *Feminism & Psychology* 2020; 30: 566.

123. Nguyen T, Nguyen T and Woo BK. Using YouTube to disseminate dementia knowledge among Chinese Australians. *Australian and New Zealand Journal of Psychiatry* 2020; 54: 941.

124. Pechmann C, Phillips C, Calder D, et al. Facebook recruitment using zip codes to improve diversity in health research: Longitudinal observational study. *Journal of Medical Internet Research Vol 22(6), 2020, ArtID e17554* 2020; 22.

125. Rodriguez Camacho JC. Intersections of ethics of indigenous health research and health research education. *Dissertation Abstracts International Section A: Humanities and Social Sciences* 2020; 81: No Pagination Specified.

126. Ryan A, Gilroy J and Gibson C. #Changethedate: Advocacy as an on-line and decolonising occupation. *Journal of Occupational Science* 2020; 27: 405-416.

127. Tartakovsky E and Walsh SD. Factors affecting the psychological well-being of immigrants: The role of group self-appraisal, social contacts, and perceived ethnic density. *Cultural Diversity and Ethnic Minority Psychology* 2020; 26: 592-603.

128. Turnbull S, Cabral C, Hay A, et al. Health equity in the effectiveness of web-based health interventions for the self-care of people with chronic health conditions: Systematic review. *Journal of Medical Internet Research Vol 22(6), 2020, ArtID e17849* 2020; 22.

129. Wilkie DJ, Yao Y, Ezenwa MO, et al. A stepped-wedge randomized controlled trial: Effects of eHealth interventions for pain control among adults with cancer in hospice. *Journal of Pain and Symptom Management* 2020; 59: 626-636.

130. Asimovic N, Nagler J, Bonneau R, et al. Testing the effects of Facebook usage in an ethnically polarized setting. *PNAS Proceedings of the National Academy of Sciences of the United States of America Vol 118(25), 2021, ArtID e2022819118* 2021; 118.

131. Conigrave JH, Bradshaw EL, Conigrave KM, et al. Alcohol consumption and dependence is linked to the extent that people experience need satisfaction while drinking alcohol in two Aboriginal and Torres Strait Islander communities. *Addiction Science & Clinical Practice Vol 16 2021, ArtID 23* 2021; 16.

132. D'Amico EJ, Dickerson DL, Rodriguez A, et al. Integrating traditional practices and social network visualization to prevent substance use: Study protocol for a randomized controlled trial among urban Native American emerging adults. *Addiction Science & Clinical Practice Vol 16 2021, ArtID 56* 2021; 16.

133. Keum BT and Cano MA. Online racism, psychological distress, and alcohol use among racial minority women and men: A multi-group mediation analysis. *American Journal of Orthopsychiatry* 2021; 91: 524-530.

134. Kevin C and Salmon F. Indigenous art in higher education: 'Palpable history' as a decolonising strategy for enhancing reconciliation and well-being. *Object-based learning and well-being: Exploring material connections*. New York, NY: Routledge/Taylor & Francis Group; US, 2021, pp.60-78.

135. Khalily MT, Bhatti MM, Ahmad I, et al. Indigenously adapted cognitive-behavioral therapy for excessive smartphone use (IACBT-ESU): A randomized controlled trial. *Psychology of Addictive Behaviors* 2021; 35: 93-101.

136. Weatherall TJ, Conigrave JH, Conigrave KM, et al. Alcohol dependence in a community sample of Aboriginal and Torres Strait Islander Australians: Harms, getting help and awareness of local treatments. *Addiction Science & Clinical Practice Vol 16 2021, ArtID 65* 2021; 16.

137. Young S. The influence of ethnicity on the impact of cyberbullying among adults. *Dissertation Abstracts International: Section B: The Sciences and Engineering* 2021; 82: No Pagination Specified.

138. Alomran AI. The use of smartphone applications for learning purposes among Saudi students. *Dissertation Abstracts International: Section B: The Sciences and Engineering* 2022; 83: No Pagination Specified.

# Embase – Ovid 2022.03.08

## Notes about this database search

There are a lot more articles than expected but they look similar to the Medline search and no obvious terms causing abundance of false positives

## Actual search terms Embase – Ovid 2022.03.08

Embase <1974 to 2022 March 04>

1 Oceanic Ancestry Group/ or alaskan natives/ or inuits/ or Indigenous Peoples/ 12796

2 (Aboriginal or First Nation).tw. 11861

3 Torres Strait.tw. 2742

4 maori.tw. 4653

5 exp American Native Continental Ancestry Group/ 20138

6 sami.tw. 671

7 Ainu.tw. 159

8 1 or 2 or 3 or 4 or 5 or 6 or 7 43606

9 blogging/ or social media/ or electronic mail/ 58495

10 internet/ or internet access/ or "internet use"/ or internet-based intervention/ 118960

11 social networking/ or online social networking/ 17703

12 social media/ 33723

13 (smartphone or "mobile application" or App or e-health or ehealth or internet or email or Facebook or SnapChat or Instagram or Twitter or TicToc or Pinterest or youtube).tw. 172993

14 9 or 10 or 11 or 12 or 13 278106

15 consumer health information/ or health literacy/ or health promotion/ or healthy people programs.mp. [mp=title, abstract, heading word, drug trade name, original title, device manufacturer, drug manufacturer, device trade name, keyword heading word, floating subheading word, candidate term word] 122365

16 weight reduction programs/ or sex education/ or smoking prevention/ 15511

17 "Quality of Life"/ 544408

18 (wellbeing or well-being or wellness).tw. 159447

19 Self Care/ 67324

20 neoplasms/ or lung diseases/ or chronic disease/ or multiple chronic conditions/ or emphysema/ or Cardiovascular Diseases/ or diabetes mellitus/ or diabetes mellitus, type 1/ or diabetes mellitus, type 2/ 1060487

21 Stress, Psychological/ 78947

22 Alcoholism/ 124409

23 "tobacco use disorder"/ 12153

24 smoking/ or smoking reduction/ or tobacco smoking/ or vaping/ or "tobacco use"/ 352830

25 Behavior, Addictive/ or addiction disorder.mp. or Substance-Related Disorders/ 59857

26 15 or 16 or 17 or 18 or 19 or 20 or 21 or 22 or 23 or 24 or 25 2334363

27 8 and 14 and 26 225

28 limit 27 to (english language and yr="2005 -Current") 223

## Link to saved search

<https://simsrad.net.ocs.mq.edu.au/login?url=http://ovidsp.ovid.com/ovidweb.cgi?T=JS&NEWS=N&PAGE=main&SHAREDSEARCHID=60mWARCLedTqI7su8tLi1FL3WU4pBDYqkxYHHCsvG89GFAHl5XwyXpFsr0pdhEUUR>

## References found by Embase-Ovid 2022.03.08

1. Bannink L, Wells S, Broad J, et al. Web-based assessment of cardiovascular disease risk in routine primary care practice in New Zealand: The first 18,000 patients (PREDICT CVD-1). *New Zealand Medical Journal* 2006; 119.

2. Hebert R. What's new in nicotine and tobacco research? *Nicotine and Tobacco Research* 2007; 9(SUPPL. 1): S1-S5.

3. Schillo BA and Willoughby DJ. Understanding tobacco use in Minnesota: Findings from the ClearWay Minnesota<sup>SM</sup> research program. *Nicotine and Tobacco Research* 2007; 9(SUPPL. 1): S7-S9. Note.

4. Simpson SA and Long JA. Medical student-run health clinics: Important contributors to patient care and medical education. *Journal of General Internal Medicine* 2007; 22(3): 352-356.

5. Buller DB, Woodall WG, Zimmerman DE, et al. Randomized trial on the 5 a day, the Rio Grande way website, a web-based program to improve fruit and vegetable consumption in rural communities. *Journal of Health Communication* 2008; 13(3): 230-249.

6. Donelle L and Hoffman-Goetz L. An exploratory study of Canadian aboriginal online health care forums. *Health Communication* 2008; 23(3): 270-281.

7. Case S, Jernigan V, Gardner A, et al. *Journal of medical Internet research* 2009; 11(2): e22.

8. Dawson K, Nabih H, DeGoeij L, et al. Diabetes and my nation: A model program for diabetes teaching and treatment in aboriginal communities. *Canadian Journal of Diabetes* 2009; 33(3): 279-280. Conference Abstract.

9. Bale B. Optimizing hypertension management in underserved rural populations. *Journal of the National Medical Association* 2010; 102(1): 10-17. Review.

10. Collins T, Lightbourne M and Henderson K. Ethnicity and perceptions of health information. *Journal of General Internal Medicine* 2010; 3): S271-S272. Conference Abstract.

11. Grey C, Wells S, Riddell T, et al. A comparative analysis of the cardiovascular disease risk factor profiles of Pacific peoples and Europeans living in New Zealand assessed in routine primary care: PREDICT CVD-11. *New Zealand Medical Journal* 2010; 123(1309): 62-75.

12. Hannah J, Humphrey G, Doughty R, et al. Telehealth in heart failure management: A proof of principle study. *Heart Lung and Circulation* 2010; 1): S13-S14. Conference Abstract.

13. Lemelin H, Matthews D, Mattina C, et al. Climate change, wellbeing and resilience in the Weenusk First Nation at Peawanuck: the Moccasin Telegraph goes global. *Rural and remote health* 2010; 10(2): 1333.

14. Maar MA, Seymour A, Sanderson B, et al. Reaching agreement for an Aboriginal e-health research agenda: the Aboriginal Telehealth Knowledge Circle consensus method. *Rural and remote health* 2010; 10(1): 1299.

15. Macnab AJ, Radziminski N, Budden H, et al. Brighter Smiles Africa--translation of a Canadian community-based health-promoting school program to Uganda. *Education for health (Abingdon, England)* 2010; 23(2): 241.

16. Steinfeldt JA, Foltz BD, Kaladow JK, et al. Racism in the electronic age: Role of online forums in expressing racial attitudes about American Indians. *Cultural Diversity and Ethnic Minority Psychology* 2010; 16(3): 362-371.

17. Brown HW, Wexner S, Segall M, et al. Predictors of and care-seeking for accidental bowel leakage: A common yet under-diagnosed condition. *International Urogynecology Journal and Pelvic Floor Dysfunction* 2011; 1): S76-S77. Conference Abstract.

18. Brown HW, Wexner S, Segall MM, et al. Prevalence, risk factors, and preferred terminology for fecal incontinence: Accidental bowel leakage. *Female Pelvic Medicine and Reconstructive Surgery* 2011; 1): S95-S96. Conference Abstract.

19. Dykstra C, Fatvich D, Flexman J, et al. Royal perth hospital emergency department screening project for chlamydia trachomatis. *Sexually Transmitted Infections* 2011; 1): A347. Conference Abstract.

20. Johnson VB and Lorig K. The internet diabetes self-management workshop for American Indians and Alaska Natives. *Health promotion practice* 2011; 12(2): 261-270.

21. Passey ME, Gale JT and Sanson-Fisher RW. "Its almost expected": Rural Australian Aboriginal women's reflections on smoking initiation and maintenance: a qualitative study. *BMC Women's Health* 2011: 55. In Press.

22. Reti SR, Feldman HJ and Safran C. Online access and literacy in Maori New Zealanders with diabetes. *Journal of primary health care* 2011; 3(3): 190-191.

23. Robinson JD, Warisse Turner J, Levine B, et al. Expanding the walls of the health care encounter: Support and outcomes for patients online. *Health Communication* 2011; 26(2): 125-134.

24. Rushing SC and Stephens D. Use of media technologies by Native American teens and young adults in the Pacific Northwest: exploring their utility for designing culturally appropriate technology-based health interventions. *The journal of primary prevention* 2011; 32(3-4): 135-145.

25. Wang Y, Lim H, Nguyen T, et al. *Obesity* 2011; 1): S213. Conference Abstract.

26. Colleran KM, Harding E, Zurawski A, et al. An innovative community health worker training program: Addressing the diabetes pandemic through expansion of the diabetes health care team. *Diabetologia* 2012; 1): S419. Conference Abstract.

27. Geana MV, Greiner KA, Cully A, et al. *Journal of community health* 2012; 37(6): 1253-1263.

28. Henderson JA, Chubak J, O'Connell J, et al. Design of a randomized controlled trial of a web-based intervention to reduce cardiovascular disease risk factors among remote reservation-dwelling American Indian adults with type 2 diabetes. *The journal of primary prevention* 2012; 33(4): 209-222.

29. Kilcullen ML, Swinbourne A and Cadet-James Y. Mental health and connectedness-exploring aboriginal and torres strait islander perspectives. *Journal of Paediatrics and Child Health* 2012; 1): 62. Conference Abstract.

30. Purnell TS, Hall YN and Boulware LE. *Advances in Chronic Kidney Disease* 2012; 19(4): 244-251. Review.

31. Samuel KA, Ribisl KM and Williams RS. Internet cigarette sales and Native American sovereignty: Political and public health contexts. *Journal of Public Health Policy* 2012; 33(2): 173-187. Review.

32. Shaeer O and Shaeer K. The Global Online Sexuality Survey (GOSS): The United States of America in 2011. Chapter I: Erectile Dysfunction Among English-Speakers. *Journal of Sexual Medicine* 2012; 9(12): 3018-3027.

33. Whitesell NR, Beals J, Crow CB, et al. Epidemiology and etiology of substance use among American Indians and Alaska natives: Risk, protection, and implications for prevention. *American Journal of Drug and Alcohol Abuse* 2012; 38(5): 376-382.

34. Anonymous. 10th International Congress on Adolescent Health. *Turkish Archives of Pediatrics* 2013; 48(Suppl 2): 1. Conference Review.

35. Browne-Yung K, Ziersch A, Baum F, et al. Aboriginal Australians' experience of social capital and its relevance to health and wellbeing in urban settings. *Social Science and Medicine* 2013; 97: 20-28.

36. Clark R, Fredericks B, Adams M, et al. Development of a culturally specific heart failure selfcare ipad teaching tool for aboriginal and Torres strait islanders. *Heart Lung and Circulation* 2013; 1): S251. Conference Abstract.

37. Coleman TA, Pugh D, Aykroyd G, et al. Sexual behaviour in gay, bisexual , and other men who have sex with men in London, Ontario : The health in Middlesex men matters (HiMMM) project survey. *Canadian Journal of Infectious Diseases and Medical Microbiology* 2013; SA): 83A. Conference Abstract.

38. Cueva M, Kuhnley R, Revels LJ, et al. Bridging storytelling traditions with digital technology. *International Journal of Circumpolar Health* 2013; 72.

39. Duncan KC, Salters K, Forrest JI, et al. Cohort profile: Longitudinal investigations into supportive and ancillary health services. *International Journal of Epidemiology* 2013; 42(4): 947-955.

40. Fernet M, Racicot C, Blais M, et al. Mother-children education to sexuality : Perspective of the Mothers Living with HIV (MLHIV). *Canadian Journal of Infectious Diseases and Medical Microbiology* 2013; SA): 121A. Conference Abstract.

41. Filippi MK, McCloskey C, Williams C, et al. *Journal of community health* 2013; 38(3): 486-491.

42. Filippi MK, McCloskey C, Williams C, et al. Perceptions, barriers, and suggestions for creation of a tobacco and health website among American Indian/Alaska Native college students. *Journal of community health* 2013; 38(3): 486-491.

43. Gould GS, McEwen A, Watters T, et al. Should anti-tobacco media messages be culturally targeted for Indigenous populations? A systematic review and narrative synthesis. *Tobacco control* 2013; 22(4): e7.

44. Jessen C, Revels T and Ferguson J. Iknowmine.org - A comprehensive wellness website for Alaska Native Youth. *Sexually Transmitted Infections Conference: STI and AIDS World Congress* 2013; 89. Conference Abstract.

45. Minde GT and Saeterstrand TM. What is important in the surroundings in order to extend the healthy life period? A regional study of 19 older women in a northern part of Norway. *International journal of circumpolar health* 2013; 72.

46. Petersen W and Nicometo A. Can theoretical mediators distinguish between persistent and sporadic mammographic screeners? A preliminary examination of American Indian women in one tribe. *Psycho-Oncology* 2013; 2): 109-110. Conference Abstract.

47. Rivers R, Saewyc EM, MacKay L, et al. Masculinities and marginalized young men's patterns of accessing health care services. *Journal of Adolescent Health* 2013; 1): S21. Conference Abstract.

48. Watanabe-Galloway S, Duran T, Stimpson JP, et al. Gaps in survey data on cancer in American Indian and Alaska Native populations: examination of US population surveys, 1960-2010. *Preventing chronic disease* 2013; 10: E39. Review.

49. Anderson G, Gleeson S, Rissel C, et al. Twitter tweets and twaddle: twittering at AHPA. National Health Promotion Conference. *Health promotion journal of Australia : official journal of Australian Association of Health Promotion Professionals* 2014; 25(2): 143-146.

50. Blinkhorn F, Wallace J, Smith L, et al. Developing leaflets to give dental health advice to aboriginal families with young children. *International Dental Journal* 2014; 64(4): 195-199.

51. Brusse C, Gardner K, McAullay D, et al. Social media and mobile apps for health promotion in Australian Indigenous populations: scoping review. *Journal of medical Internet research* 2014; 16(12): e280. Review.

52. Clark RA, Fredericks B, Adams M, et al. Addressing health literacy and cultural teaching issues in australian indigenous and non-indigenous heart failure patients using avatars: Technology development and pilot testing. *Journal of Cardiac Failure* 2014; 1): S115. Conference Abstract.

53. Currie LM, Ronquillo C and Dick T. Access to internet in rural and remote Canada. *Studies in health technology and informatics* 2014; 201: 407-412.

54. Firestone M, Smylie J, Maracle S, et al. Unmasking health determinants and health outcomes for urban First Nations using respondent-driven sampling. *BMJ Open* 2014; 4(7) (no pagination).

55. Glover M, Kira A, Gentles D, et al. The WERO group stop smoking competition: main outcomes of a pre- and post- study. *BMC public health* 2014; 14: 599.

56. Hume A, Wetten A, Feeney C, et al. Remote school gardens: exploring a cost-effective and novel way to engage Australian Indigenous students in nutrition and health. *Australian and New Zealand journal of public health* 2014; 38(3): 235-240.

57. Nystad K, Spein AR and Ingstad B. Community resilience factors among indigenous Sami adolescents: A qualitative study in Northern Norway. *Transcultural Psychiatry* 2014; 51(5): 651-672.

58. Pesko MF, Xu X, Tynan MA, et al. Per-pack price reductions available from different cigarette purchasing strategies: United States, 2009-2010. *Preventive Medicine* 2014; 63: 13-19.

59. Ram FS and McNaughton W. Giving Asthma Support to Patients (GASP): a novel online asthma education, monitoring, assessment and management tool. *Journal of primary health care* 2014; 6(3): 238-244.

60. Redfern J, Usherwood T, Harris MF, et al. A randomised controlled trial of a consumer-focused e-health strategy for cardiovascular risk management in primary care: The Consumer Navigation of Electronic Cardiovascular Tools (CONNECT) study protocol. *BMJ Open* 2014; 4(2) (no pagination).

61. Shah VO, Ghahate DM, Bobelu J, et al. Identifying barriers to healthcare to reduce health disparity in Zuni Indians using focus group conducted by community health workers. *Clinical and Translational Science* 2014; 7(1): 6-11.

62. Westermeyer J. Alcoholics anonymous and spiritual recovery: A cultural perspective. *Alcoholism Treatment Quarterly* 2014; 32(2-3): 157-172.

63. Bradford D, Hansen D and Karunanithi M. Making an APPropriate Care Program for Indigenous Cardiac Disease: Customization of an Existing Cardiac Rehabilitation Program. *Studies in health technology and informatics* 2015; 216: 343-347.

64. Burhansstipanov L, Krebs LU and Harjo LD. Challenges in addressing cancer health disparities in Native American communities. *Cancer Epidemiology Biomarkers and Prevention Conference: 7th AACR Conference on the Science of Health Disparities in Racial/Ethnic Minorities and the Medically Underserved San Antonio, TX United States Conference Publication:* 2015; 24. Conference Abstract.

65. Campbell AN, Turrigiano E, Moore M, et al. Acceptability of a web-based community reinforcement approach for substance use disorders with treatment-seeking American Indians/Alaska Natives. *Community mental health journal* 2015; 51(4): 393-403.

66. Chambers RA, Rosenstock S, Neault N, et al. A Home-Visiting Diabetes Prevention and Management Program for American Indian Youth: The Together on Diabetes Trial. *The Diabetes educator* 2015; 41(6): 729-747.

67. Jones EJ, Peercy M, Woods JC, et al. Identifying postpartum intervention approaches to reduce cardiometabolic risk among American Indian women with prior gestational diabetes, Oklahoma, 2012-2013. *Preventing chronic disease* 2015; 12: E45.

68. Sweet M, Geia L, Dudgeon P, et al. #IHMayDay: tweeting for empowerment and social and emotional wellbeing. *Australasian psychiatry : bulletin of Royal Australian and New Zealand College of Psychiatrists* 2015; 23(6): 636-640.

69. Young NL, Wabano MJ, Usuba K, et al. Title: Reliability of the Aboriginal Children's Health and Well-Being Measure (ACHWM). *Quality of Life Research* 2015; 24(1 Supplement 1): 158-159. Conference Abstract.

70. Bartgis J and Albright G. Online role-play simulations with emotionally responsive avatars for the early detection of Native youth psychological distress, including depression and suicidal ideation. *American Indian and Alaska native mental health research (Online)* 2016; 23(2): 1-27.

71. Bodhani AR, Suryavanshi M, Medema J, et al. Health related quality of life among patients with erosive hand osteoarthritis in the United States. *Osteoarthritis and Cartilage* 2016; 1): S238. Conference Abstract.

72. Burhansstipanov L, Petereit D, Sargent M, et al. Findings: The walking forward American Indian survivorship physical activity pilot program. *Psycho-Oncology* 2016; 2): 49. Conference Abstract.

73. Gritton JC, Rushing SC, Stephens D, et al. "People care": Recommendations from native youth to address concerning mental health displays on social media. *Journal of Adolescent Health* 2016; 1): S58. Conference Abstract.

74. Gwynne K, Flaskas Y, O'Brien C, et al. *BMJ Open* 2016; 6(11) (no pagination).

75. Lopez-Sublet M, Baguet JP, Courand PY, et al. *Journal of Hypertension* 2016; 34(Supplement 2): e161-e162. Conference Abstract.

76. Munnelly SW. Next steps in addressing sociodemographic and mental health disparities of American Indian/Alaska native youth. *Journal of the American Academy of Child and Adolescent Psychiatry* 2016; 55(10 Supplement 1): S10. Conference Abstract.

77. Nathan S, Rawstorne P, Hayen A, et al. Examining the pathways for young people with drug and alcohol dependence: A mixed-method design to examine the role of a treatment programme. *BMJ Open* 2016; 6(5) (no pagination).

78. Petersen W, Nicometo A, Vierkant R, et al. *Psycho-Oncology* 2016; 2): 58. Conference Abstract.

79. Petersen WO, Nicometo AM and Sabaque C. *Cancer Epidemiology Biomarkers and Prevention Conference: 8th AACR Conference on the Science of Health Disparities in Racial/Ethnic Minorities and the Medically Underserved Atlanta, GA United States* 2016; 25. Conference Abstract.

80. Povey J, Mills PP, Dingwall KM, et al. Acceptability of Mental Health Apps for Aboriginal and Torres Strait Islander Australians: A Qualitative Study. *Journal of medical Internet research* 2016; 18(3): e65.

81. Wolf DAPS, Tovar M, Thompson K, et al. Speaking out about physical harms from tobacco use: Response to graphic warning labels among American Indian/Alaska Native communities. *BMJ Open* 2016; 6(3) (no pagination).

82. Zhang Z, Monro J and Venn B. Exploring emotions, knowledge and nutritional support in different ethnic groups living in new zealand with pre-diabetes and type 2 diabetes. *Revista Espanola de Nutricion Humana y Dietetica* 2016; 20(Supplement 1): 498-499. Conference Abstract.

83. Zywiak WH, Stout RL, Braciszewski JM, et al. Test-retest reliability and descriptive analyses of the modified important people and activities (MIPA) interview. *Heroin Addiction and Related Clinical Problems* 2016; 18(2): 41-48.

84. Bennett-Levy J, Singer J, DuBois S, et al. Translating E-Mental Health Into Practice: What Are the Barriers and Enablers to E-Mental Health Implementation by Aboriginal and Torres Strait Islander Health Professionals? *Journal of medical Internet research* 2017; 19(1): e1.

85. Bensemann C. A whole-of-system MH&A integration transformation agenda: Challenges and early successes. *Australian and New Zealand Journal of Psychiatry* 2017; 51(1 Supplement 1): 97. Conference Abstract.

86. Garcia A, Baethke L and Kaur JS. Lessons Learned from Native C.I.R.C.L.E., a Culturally Specific Resource. *Journal of cancer education : the official journal of the American Association for Cancer Education* 2017; 32(4): 740-744.

87. George SA, Krishnamurthi R, Feigin V, et al. E-health for primary stroke prevention in New Zealand: A novel technological approach for holistic risk management? *International Journal of Stroke* 2017; 12(3 Supplement 1): 46. Conference Abstract.

88. Holt CL and Chambers DA. Opportunities and challenges in conducting community-engaged dissemination/implementation research. *Translational Behavioral Medicine* 2017; 7(3): 389-392. Editorial.

89. Janca A, Balaratnasingam S and Lee J. Advancing the social and emotional well-being of aboriginal and Torres Strait Islander people. *Australian and New Zealand Journal of Psychiatry* 2017; 51(1 Supplement 1): 47-48. Conference Abstract.

90. Janca A, Lyons Z and Gaspar J. *Australasian Psychiatry* 2017; 25(3): 288-289.

91. Kwan P, Sabado-Liwag M, Lee C, et al. Development of an Online Smoking Cessation Curriculum for Pacific Islanders: A Community-Based Participatory Research Approach. *Progress in community health partnerships : research, education, and action* 2017; 11(3): 263-274.

92. Macniven R, Elwell M, Ride K, et al. A snapshot of physical activity programs targeting Aboriginal and Torres Strait Islander people in Australia. *Health promotion journal of Australia : official journal of Australian Association of Health Promotion Professionals* 2017; 28(3): 185-206.

93. Nguyen AB, Robinson J, O'Brien EK, et al. Racial and Ethnic Differences in Tobacco Information Seeking and Information Sources: Findings From the 2015 Health Information National Trends Survey. *Journal of health communication* 2017; 22(9): 743-752.

94. Patel I, Srivastava J, Chang J, et al. Diabetes mellitus self-management interventions in Latino adults in The United States-the role of pharmacists. *Value in Health* 2017; 20(9): A485. Conference Abstract.

95. Perkes S, Gruppetta M, Bonevski B, et al. Mothers aunties maternal aboriginal smokers (mamas) study. *Asia-Pacific Journal of Clinical Oncology* 2017; 13(Supplement 5): 31. Conference Abstract.

96. Rosenbaum DL, Piers AD, Schumacher LM, et al. Racial and ethnic minority enrollment in randomized clinical trials of behavioural weight loss utilizing technology: a systematic review. *Obesity Reviews* 2017; 18(7): 808-817. Review.

97. Salsberg J, Macridis S, Bengoechea EG, et al. Engagement strategies that foster community self-determination in participatory research: Insider ownership through outsider championship. *Family Practice* 2017; 34(3): 336-340.

98. Salsberg J, Macridis S, Bengoechea EG, et al. The shifting dynamics of social roles and project ownership over the lifecycle of a communitybased participatory research project. *Family Practice* 2017; 34(3): 305-312.

99. Schwinn T, Hopkins J, Schinke SP, et al. Using Facebook ads with traditional paper mailings to recruit adolescent girls for a clinical trial. *Addictive Behaviors* 2017; 65: 207-213.

100. Tomayko EJ, Prince RJ, Cronin KA, et al. Healthy Children, Strong Families 2: A randomized controlled trial of a healthy lifestyle intervention for American Indian families designed using community-based approaches. *Clinical Trials* 2017; 14(2): 152-161.

101. Towns S, DiFranza JR, Jayasuriya G, et al. Smoking Cessation in Adolescents: targeted approaches that work. *Paediatric Respiratory Reviews* 2017; 22: 11-22. Review.

102. Winch S, Ahmed N, Rissel C, et al. The reach and flow of health information in two Aboriginal communities: A social network analysis. *Australian Journal of Primary Health* 2017; 23(2): 189-195.

103. Anonymous. *Women and Birth Conference: The Australian College of Midwives* 2018; 31. Conference Review.

104. Aschbrenner KA, Bobak C, Schneider EJ, et al. Egocentric social networks and smoking among adults with serious mental illness. *Translational Behavioral Medicine* 2018; 8(4): 531-539.

105. Black KJ, Morse B, Tuitt N, et al. Beyond Content: Cultural Perspectives on Using the Internet to Deliver a Sexual Health Intervention to American Indian Youth. *The journal of primary prevention* 2018; 39(1): 59-70.

106. Broad K, Petrakis I, Edens E, et al. Digital natives in a tele-medical world: Evaluation of a pilot educational elective in teleaddiction and role of tele-medical education in addiction fellowship. *American Journal on Addictions* 2018; 27(4): 278-279. Conference Abstract.

107. Chedid RA, Terrell RM and Phillips KP. Best practices for online Canadian prenatal health promotion: A public health approach. *Women and Birth* 2018; 31(4): e223-e231.

108. Dockery J, Langner R and WhiteWilliams C. Nursing knowledge and attitudes regarding pain in a tertiary hospital in the southeastern United States. *Postgraduate Medicine* 2018; 130(Supplement 1): 29-30. Conference Abstract.

109. Eagle M. Street shot: Preventing hepatitis through art. *Journal of Viral Hepatitis* 2018; 25(Supplement 2): 186. Conference Abstract.

110. Hamilton SJ, Mills B, Birch EM, et al. Smartphones in the secondary prevention of cardiovascular disease: A systematic review. *BMC Cardiovascular Disorders* 2018; 18(1) (no pagination).

111. Hefler M, Kerrigan V and Thomas DP. Can Facebook help reduce smoking? A qualitative study to investigate how to use social media for tobacco control among Australian Indigenous people. *Tobacco Induced Diseases* 2018; 16(Supplement 1): 183-184. Conference Abstract.

112. Kariippanon K and Senior K. Re-thinking knowledge landscapes in the context of grounded aboriginal theory and online health communication. *Croatian Medical Journal* 2018; 59(1): 33-38.

113. Lombard C, Brennan L, Reid M, et al. Communicating health-Optimising young adults' engagement with health messages using social media: Study protocol. *Nutrition & dietetics: the journal of the Dietitians Association of Australia* 2018; 75(5): 509-519.

114. Mai Y and Soulakova JN. Retrospective reports of former smokers: Receiving doctor's advice to quit smoking and using behavioral interventions for smoking cessation in the United States. *Preventive Medicine Reports* 2018; 11: 290-296.

115. McFarlane KA, Judd J, Wapau H, et al. How primary health care staff working in rural and remote areas access skill development and expertise to support health promotion practice. *Rural and remote health* 2018; 18(2): 4413.

116. Narcisse MR, Felix H, Long CR, et al. Frequency and predictors of health services use by Native Hawaiians and Pacific Islanders: evidence from the U.S. National Health Interview Survey. *BMC health services research* 2018; 18(1): 575.

117. Pereira-Salgado A, Mader P and Boyd LM. Advance care planning, culture and religion: an environmental scan of Australian-based online resources. *Australian health review : a publication of the Australian Hospital Association* 2018; 42(2): 152-163. Review.

118. Rao VG, Muniyandi M, Bhat J, et al. Research on tuberculosis in tribal areas in India: A systematic review. *Indian Journal of Tuberculosis* 2018; 65(1): 8-14. Review.

119. Regenstein M and Seyoum S. *Pediatric Pulmonology* 2018; 53(Supplement 2): 71-72. Conference Abstract.

120. Rushing SNC, Stephens D and Dog TLG. We r native: Harnessing technology to improve health outcomes for american indian and alaska native youth. *Journal of Adolescent Health* 2018; 62(2 Supplement 1): S83-S84. Conference Abstract.

121. Sarfati D, McLeod M, Stanley J, et al. BetaMe: Impact of a comprehensive digital health programme on HbA1c and weight at 12 months for people with diabetes and pre-diabetes: Study protocol for a randomised controlled trial. *Trials* 2018; 19(1) (no pagination).

122. Wong G, Glover M, McPherson M, et al. Boosting efficacy of nurse-led stop smoking interventions with a quit and win contest: pilot study results. *Contemporary nurse* 2018; 54(4-5): 395-408.

123. Arrom-Suhurt CM, Arrom-Suhurt CH, Arrom-Suhurt MA, et al. Socioeconomic profile and perceptions of Chagas disease in indigenous communities of the Paraguayan Chaco. *Journal of Public Health (Germany)* 2019; 27(6): 723-732.

124. Banerji S, Melvin K, Rooks K, et al. Prolonged toxicity following selfadministration of a kambo cleansing ritual. *Clinical Toxicology* 2019; 57(6): 561-562. Conference Abstract.

125. Beekman J, Keisler A, Pedraza O, et al. Neuromyelitis optica spectrum disorder: Patient experience and quality of life. *Neurology: Neuroimmunology and NeuroInflammation* 2019; 6(4) (no pagination).

126. Bryan JM. Letters: Public trust in the information age. *Cmaj* 2019; 191(32): E899. Letter.

127. Dimitropoulos Y, Holden A and Sohn W. In-school toothbrushing programs in Aboriginal communities in New South Wales, Australia: A thematic analysis of teachers' perspectives. *Community dental health* 2019; 36(2): 106-110.

128. Dingwall KM, Nagel T, Hughes JT, et al. Wellbeing intervention for chronic kidney disease (WICKD): a randomised controlled trial study protocol. *BMC psychology* 2019; 7(1): 2.

129. Elman A, Etter M, Fairman K, et al. Mental health services in the Northwest Territories: a scoping review. *International journal of circumpolar health* 2019; 78(1): 1629783.

130. Hefler M, Kerrigan V, Freeman B, et al. Using Facebook to reduce smoking among Australian Aboriginal and Torres Strait Islander people: a participatory grounded action study. *BMC public health* 2019; 19(1): 615.

131. Hefler M, Kerrigan V, Henryks J, et al. Social media and health information sharing among Australian Indigenous people. *Health promotion international* 2019; 34(4): 706-715.

132. Hirchak KA, Herron J, Murphy SM, et al. Contingency management as an intervention for alcohol misuse among American Indian young adults: Interest and considerations for cultural acceptability. *Alcoholism: Clinical and Experimental Research* 2019; 43(Supplement 1): 194A. Conference Abstract.

133. Hutt-MacLeod D, Rudderham H, Sylliboy A, et al. Eskasoni First Nation's transformation of youth mental healthcare: Partnership between a Mi'kmaq community and the ACCESS Open Minds research project in implementing innovative practice and service evaluation. *Early Intervention in Psychiatry* 2019; 13(S1): 42-47.

134. Kirkham R, MacKay D, Barzi F, et al. Improving postpartum screening after diabetes in pregnancy: Results of a pilot study in remote Australia. *Australian and New Zealand Journal of Obstetrics and Gynaecology* 2019; 59(3): 430-435.

135. Lee KSK, Conigrave JH, Callinan S, et al. Asking about the last four drinking occasions on a tablet computer as a way to record alcohol consumption in Aboriginal and Torres Strait Islander Australians: a validation. *Addiction science & clinical practice* 2019; 14(1): 15.

136. McCrabb S, Twyman L, Palazzi K, et al. A cross sectional survey of internet use among a highly socially disadvantaged population of tobacco smokers. *Addiction science & clinical practice* 2019; 14(1): 38.

137. Min J and Park J. Relying on pharmacy students as digital natives in practice: Surprising gaps in digital health literacy. *Canadian Pharmacists Journal* 2019; 152(5): S42. Conference Abstract.

138. Nghiem N, Leung W, Cleghorn C, et al. Mass media promotion of a smartphone smoking cessation app: modelled health and cost-saving impacts. *BMC public health* 2019; 19(1): 283.

139. Ni Mhurchu C, Te Morenga L, Tupai-Firestone R, et al. A co-designed mHealth programme to support healthy lifestyles in Maori and Pasifika peoples in New Zealand (OL@-OR@): a cluster-randomised controlled trial. *The Lancet* 2019; Digital health. 1(6): e298-e307.

140. Ni Mhurchu C, Te Morenga L, Tupai-Firestone R, et al. A co-designed mHealth programme to support healthy lifestyles in Maori and Pasifika peoples in New Zealand (OL@-OR@): a cluster-randomised controlled trial. *The Lancet Digital Health* 2019; 1(6): e298-e307.

141. Pedroni C, Dujeu M, Lebacq T, et al. Alcohol consumption in early adolescence is differently associated with sociodemographic and psychosocial factors according to gender (HBSC 2014, Wallonia, Belgium). *Proceedings of the Nutrition Society Conference: 13th European Nutrition Conference, FENS* 2019; 79. Conference Abstract.

142. Peiris D, Wright L, News M, et al. Community-Based Chronic Disease Prevention and Management for Aboriginal People in New South Wales, Australia: Mixed Methods Evaluation of the 1 Deadly Step Program. *JMIR mHealth and uHealth* 2019; 7(10): e14259.

143. Regan EA, Vaidya A, Margulies PL, et al. Primary adrenal insufficiency in the United States: Diagnostic error and patient satisfaction with treatment. *Diagnosis* 2019; 6(4): 343-350.

144. Robards F, Kang M, Steinbeck K, et al. *International Journal for Equity in Health* 2019; 18(1) (no pagination).

145. Saxton P, Hammoud M, Andrews S, et al. 'flux NZ': An online national cohort investigating HIV, STI and drug-related practices among New Zealand gay and bisexual men. *Sexually Transmitted Infections* 2019; 95(Supplement 1): A251. Conference Abstract.

146. Shand F, MacKinnon A, O'Moore K, et al. The iBobbly Aboriginal and Torres Strait Islander app project: Study protocol for a randomised controlled trial. *Trials* 2019; 20(1) (no pagination).

147. Sinka V. Mon-333 Walking through This Great Southern Land: Mapping the Existing Indigenous Health Prevention Programs in Chronic Disease in Nsw Australia. *Kidney International Reports* 2019; 4(7 Supplement): S436-S437. Conference Abstract.

148. Small S, Porr C and Murray C. Smoking in pregnancy in aboriginal women: Not simply a "selfish Choice". *Canadian Journal of Respiratory, Critical Care, and Sleep Medicine* 2019; 3(Supplement 1): 48. Conference Abstract.

149. Starblanket D and Legare M. Testing Locally Developed Language Apps to Reduce Caregiver Stress and Promote "Aging in Place" as It Relates to Dementia in Indigenous Populations. *Alzheimer's and Dementia* 2019; 15(7 Supplement): P1450. Conference Abstract.

150. Starblanket D and Legare M. Testing Locally Developed Language Apps to Reduce Caregiver Stress and Aging in Place as It Relates to Dementia in Indigenous Populations. *Alzheimer's and Dementia* 2019; 15(7 Supplement): P156. Conference Abstract.

151. Stotz SA, Brega AG and Moore KR. Online diabetes nutrition education for american indian and alaska native adults with type 2 diabetes: Stakeholder perspectives. *Diabetes Conference: 79th Scientific Sessions of the American Diabetes Association, ADA* 2019; 68. Conference Abstract.

152. Thomas DP, Davey ME, van der Sterren AE, et al. Social networks and quitting in a national cohort of Australian Aboriginal and Torres Strait Islander smokers. *Drug and alcohol review* 2019; 38(1): 82-91.

153. Venner KL, Hagler K, Cloud V, et al. Native americans resolve alcohol use disorder: "whatever it takes or all that it takes". *Cultural Diversity and Ethnic Minority Psychology* 2019; 25(3): 350-358.

154. Verbiest MEA, Corrigan C, Dalhousie S, et al. Using codesign to develop a culturally tailored, behavior change mHealth intervention for indigenous and other priority communities: A case study in New Zealand. *Translational Behavioral Medicine* 2019; 9(4): 720-736.

155. Walker N, Smith B, Barnes J, et al. Cytisine versus varenicline for smoking cessation for Maori (the indigenous people of New Zealand) and their extended family: protocol for a randomized non-inferiority trial. *Addiction (Abingdon, England)* 2019; 114(2): 344-352.

156. Wyeth EH, Lambert M, Samaranayaka A, et al. Subsequent injuries experienced by maori: Results from a 24-month prospective study in New Zealand. *New Zealand Medical Journal* 2019; 132(1499): 23-35.

157. Ali-Hassan H, Eloulabi R and Keethakumar A. Internet non-use among Canadian indigenous older adults: Aboriginal Peoples Survey (APS). *BMC public health* 2020; 20(1): 1554.

158. Arnaudova I, Jin H and Amaro H. Pretreatment social network characteristics relate to increased risk of dropout and unfavorable outcomes among women in a residential treatment setting for substance use. *Journal of Substance Abuse Treatment* 2020; 116 (no pagination).

159. Blessing E, Virani S and Rotrosen J. Clinical trials for opioid use disorder. *Handbook of Experimental Pharmacology* 2020; 258: 167-202. Chapter.

160. Boyd AD, Fyfe-Johnson AL, Noonan C, et al. Communication With American Indians and Alaska Natives About Cardiovascular Disease. *Preventing chronic disease* 2020; 17: E160.

161. Carroll DM, Soto C, Baezconde-Garbanati L, et al. Tobacco Industry Marketing Exposure and Commercial Tobacco Product Use Disparities among American Indians and Alaska Natives. *Substance use & misuse* 2020; 55(2): 261-270.

162. Cubillo B, McCartan J, West C, et al. A qualitative analysis of the accessibility and connection to traditional food for aboriginal chronic maintenance hemodialysis Patients. *Current Developments in Nutrition* 2020; 4(4) (no pagination).

163. Der Vartanian C, Milch V, Anderiesz C, et al. Cancer and COVID-19: Delivering strategic health communication during a pandemic. *Asia-Pacific Journal of Clinical Oncology* 2020; 16(SUPPL 8): 75-76. Conference Abstract.

164. Diaz de Leon-Martinez L, de la Sierra-de la Vega L, Palacios-Ramirez A, et al. Critical review of social, environmental and health risk factors in the Mexican indigenous population and their capacity to respond to the COVID-19. *Science of the Total Environment* 2020; 733 (no pagination).

165. Dooley DG, Bandealy A and Tschudy MM. *JAMA Pediatrics* 2020; 174(10): 924-925. Note.

166. Firestone R, Cheng S, Dalhousie S, et al. Exploring Pasifika wellbeing: findings from a large cluster randomised controlled trial of a mobile health intervention programme. *The New Zealand medical journal* 2020; 133(1524): 82-101.

167. Gould GS, LaHera Fuentes G, Bovill M, et al. Aligning tobacco control and smoking cessation messages with the needs of pregnant Aboriginal and Torres Strait Islander women: A rapid review. *Asia-Pacific Journal of Clinical Oncology* 2020; 16(SUPPL 6): 22. Conference Abstract.

168. Hanson JD, Weber TL, Shrestha U, et al. Acceptability of an eHealth Intervention to Prevent Alcohol-Exposed Pregnancy Among American Indian/Alaska Native Teens. *Alcoholism: Clinical and Experimental Research* 2020; 44(1): 196-202.

169. Hefler M, Kerrigan V, Grunseit A, et al. Facebook-based social marketing to reduce smoking in Australia's first nations communities: An analysis of reach, shares, and likes. *Journal of Medical Internet Research* 2020; 22(12) (no pagination).

170. Hood AM, Nwankwo C, McTate E, et al. Mobile health use predicts self-efficacy and self-management in adolescents with sickle cell disease. *Blood* 2020; 136(SUPPL 1): 57-58. Conference Abstract.

171. Hood AM, Nwankwo C, McTate E, et al. Mobile Health Use Predicts Self-Efficacy and Self-Management in Adolescents with Sickle Cell Disease. *Blood* 2020; 136(Supplement 1): 57-58. Conference Abstract.

172. Hummel B, Bierstone D, Jetty R, et al. Child health promotion through community educational sessions in an urban inuit community: A needs assessment. *Paediatrics and Child Health (Canada)* 2020; 25(SUPPL 2): e39-e40. Conference Abstract.

173. Knox M, Skan J, Benowitz NL, et al. Recruitment best practices of a cardiovascular risk reduction randomised control trial in rural Alaska Native communities. *International journal of circumpolar health* 2020; 79(1): 1806639.

174. McGovern TF, Bundoc-Baronia R and Bergeson SE. Alcoholism Treatment Quarterly 2020, 38-4. *Alcoholism Treatment Quarterly* 2020; 38(4): 401-402. Editorial.

175. McLachlan R. Plenary lecture 1: The healthy male - Evolution of Australia's men's health programme. *Andrology* 2020; 8(SUPPL 2): 27. Conference Abstract.

176. Nagel T, Sweet M, Dingwall KM, et al. *BMC Nephrology* 2020; 21(1) (no pagination).

177. Povey J, Sweet M, Nagel T, et al. Drafting the Aboriginal and Islander Mental Health Initiative for Youth (AIMhi-Y) App: Results of a formative mixed methods study. *Internet Interventions* 2020; 21 (no pagination).

178. Preis H, Mahaffey B, Heiselman C, et al. Vulnerability and resilience to pandemic-related stress among U.S. women pregnant at the start of the COVID-19 pandemic. *Social Science and Medicine* 2020; 266 (no pagination).

179. Smith JA, Merlino A, Christie B, et al. 'Dudes Are Meant to be Tough as Nails': The Complex Nexus Between Masculinities, Culture and Health Literacy From the Perspective of Young Aboriginal and Torres Strait Islander Males - Implications for Policy and Practice. *American journal of men's health* 2020; 14(3): 1557988320936121.

180. Tighe J, Shand F, McKay K, et al. Usage and acceptability of the iBobbly app: Pilot trial for suicide prevention in aboriginal and torres strait islander youth. *JMIR Mental Health* 2020; 7(12) (no pagination).

181. Backholer K, Baum F, Finlay SM, et al. Australia in 2030: what is our path to health for all? *Medical Journal of Australia* 2021; 214(S8): S5-S40.

182. Bovill M, Chamberlain C, Bennett J, et al. Building an indigenous-led evidence base for smoking cessation care among aboriginal and torres strait islander women during pregnancy and beyond: Research protocol for the which way? project. *International Journal of Environmental Research and Public Health* 2021; 18(3): 1-11.

183. Burness C, Corbet C, Beyene K, et al. Factors predicting high-dose and combined antipsychotic prescribing in New Zealand: High-dose antipsychotic prescribing. *Psychiatry Research* 2021; 302 (no pagination).

184. Carlson B, Frazer R and Farrelly T. "That makes all the difference": Aboriginal and Torres Strait Islander health-seeking on social media. *Health promotion journal of Australia : official journal of Australian Association of Health Promotion Professionals* 2021; 32(3): 523-531.

185. D'Amico EJ, Dickerson DL, Rodriguez A, et al. *Addiction science & clinical practice* 2021; 16(1): 56.

186. Der Vartanian C, Milch V, Garvey G, et al. COVID-19 and cancer: Strategic health promotion for indigenous Australians during a pandemic. *Journal of Clinical Oncology Conference: Annual Meeting of the American Society of Clinical Oncology, ASCO* 2021; 39. Conference Abstract.

187. Dick A, Holyk T, Taylor D, et al. Highlighting strengths and resources that increase ownership of cervical cancer screening for Indigenous communities in Northern British Columbia: Community-driven approaches. *International Journal of Gynecology and Obstetrics* 2021; 155(2): 211-219.

188. El-Bassel N, Shoptaw S, Goodman-Meza D, et al. Addressing long overdue social and structural determinants of the opioid epidemic. *Drug and Alcohol Dependence* 2021; 222 (no pagination). Editorial.

189. Flemington T, La Hera-Fuentes G, Bovill M, et al. Smoking cessation messages for pregnant aboriginal and torres strait islander women: A rapid review of peer-reviewed literature and assessment of research translation of media content. *International Journal of Environmental Research and Public Health* 2021; 18(17) (no pagination). Review.

190. Grout L, Telfer K, Wilson N, et al. Prescribing Smartphone Apps for Physical Activity Promotion in Primary Care: Modeling Study of Health Gain and Cost Savings. *Journal of Medical Internet Research* 2021; 23(12) (no pagination).

191. Hale JW, Pacheco JA, Lewis CS, et al. Everyday discrimination for American Indian tribal college students enrolled in the Internet All Nations Breath of Life program. *Journal of American college health : J of ACH* 2021: 1-7.

192. Hale L, Devan H, Davies C, et al. Clinical and cost-effectiveness of an online-delivered group-based pain management programme in improving pain-related disability for people with persistent pain - Protocol for a non-inferiority randomised controlled trial (iSelf-help trial). *BMJ Open* 2021; 11(2) (no pagination).

193. Immanuel J, Eagleton C, Baker J, et al. Pregnancy outcomes among multi-ethnic women with different degrees of hyperglycaemia during pregnancy in an urban New Zealand population and their association with postnatal HbA1c uptake. *Australian and New Zealand Journal of Obstetrics and Gynaecology* 2021; 61(1): 69-77.

194. Irfan A, Lever JM, Fouad MN, et al. Does health literacy impact technological comfort in cancer patients? *American Journal of Surgery* 2021.

195. Kennedy M, Kumar R, Ryan NM, et al. Codeveloping a multibehavioural mobile phone app to enhance social and emotional well-being and reduce health risks among Aboriginal and Torres Strait Islander women during preconception and pregnancy: A three-phased mixed-methods study. *BMJ Open* 2021; 11(11) (no pagination).

196. Lane-Fall MB, Butler PD and Mahoney KB. Promoting Racial Justice and Equity in Academic Medicine: Moving beyond the Honeymoon Period. *JAMA - Journal of the American Medical Association* 2021; 326(7): 603-604. Note.

197. Lee KK, Conigrave JH, Al Ansari M, et al. Acceptability and feasibility of a computer-based application to help Aboriginal and Torres Strait Islander Australians describe their alcohol consumption. *Journal of Ethnicity in Substance Abuse* 2021; 20(1): 16-33.

198. Merculieff ZT, Koller KR, Sinicrope PS, et al. Developing a Social Media Intervention to Connect Alaska Native People Who Smoke with Resources and Support to Quit Smoking: The Connecting Alaska Native Quit Study. *Nicotine and Tobacco Research* 2021; 23(6): 1002-1009.

199. Norris P, Cousins K, Churchward M, et al. Recruiting people facing social disadvantage: the experience of the Free Meds study. *International Journal for Equity in Health* 2021; 20(1) (no pagination).

200. Robles B, Kuo T and Tobin CST. What are the relationships between psychosocial community characteristics and dietary behaviors in a racially/ethnically diverse urban population in los angeles county? *International Journal of Environmental Research and Public Health* 2021; 18(18) (no pagination).

201. Rushing SC, Kelley A, Bull S, et al. *JMIR Mental Health* 2021; 8(9) (no pagination).

202. Scholes-Robertson N, Blazek K, Tong A, et al. The financial burden of chronic kidney disease for rural Australian families. *Nephrology* 2021; 26(SUPPL 2): 36. Conference Abstract.

203. Serlachius A, Boggiss A, Lim D, et al. Pilot study of a well-being app to support New Zealand young people during the COVID-19 pandemic. *Internet Interventions* 2021; 26 (no pagination).

204. Shaaban A, Abecasis A, Fronteira I, et al. Socioeconomic impact of COVID-19 among immigrants in Lisbon Region. *Tropical Medicine and International Health* 2021; 26(SUPPL 1): 14. Conference Abstract.

205. Sifuentes JE, York E and Fultineer C. Social resilience and climate change: findings from community listening sessions. *The Lancet Planetary Health* 2021; 5(Supplement 1): S3. Conference Abstract.

206. Sinka V, Lopez-Vargas P, Tong A, et al. Chronic disease prevention programs offered by Aboriginal Community Controlled Health Services in New South Wales, Australia. *Australian and New Zealand journal of public health* 2021; 45(1): 59-64.

207. Skinner J, Dimitropoulos Y, Moir R, et al. A graduate oral health therapist program to support dental service delivery and oral health promotion in Aboriginal communities in New South Wales, Australia. *Rural and remote health* 2021; 21(1): 5789.

208. Smith JA, Merlino A, Christie B, et al. Using social media in health literacy research: A promising example involving Facebook with young Aboriginal and Torres Strait Islander males from the Top End of the Northern Territory. *Health promotion journal of Australia : official journal of Australian Association of Health Promotion Professionals* 2021; 32(Supplement 1): 186-191.

209. Snijder M, Stapinski L, Ward J, et al. Strong and deadly futures: Co-development of a web-based wellbeing and substance use prevention program for aboriginal and torres strait islander and non-aboriginal adolescents. *International Journal of Environmental Research and Public Health* 2021; 18(4): 1-25.

210. Sofija E, Harris N, Sebar B, et al. Who are the flourishing emerging adults on the urban east coast of Australia? *International Journal of Environmental Research and Public Health* 2021; 18(3): 1-15.

211. Strudwick G, Sockalingam S, Kassam I, et al. Digital interventions to support population mental health in canada during the covid-19 pandemic: Rapid review. *JMIR Mental Health* 2021; 8(3) (no pagination). Review.

212. Sussman S, Unger JB, Begay C, et al. Prevalence, Co-Occurrence, and Correlates of Substance and Behavioral Addictions Among American Indian Adolescents in California. *Journal of Drug Education* 2021; 50(1-2): 31-44.

213. Sze Cheng VW, Piper SE, Ottavio A, et al. Recommendations for designing health information technologies for mental health drawn from self-determination theory and co-design with culturally diverse populations: Template analysis. *Journal of Medical Internet Research* 2021; 23(2) (no pagination).

214. Tam MT, Dosso JA and Robillard JM. The Impact of a Global Pandemic on People Living with Dementia and Their Care Partners: Analysis of 417 Lived Experience Reports. *Journal of Alzheimer's Disease* 2021; 80(2): 865-875.

215. Tomayko EJ, Webber EJ, Cronin KA, et al. Use of Text Messaging and Facebook Groups to Support the Healthy Children, Strong Families 2 Healthy Lifestyle Intervention for American Indian Families. *Current Developments in Nutrition* 2021; 5: 32-39.

216. Van Hout MC. Foreword. *Journal of Ethnicity in Substance Abuse* 2021; 20(1): 1-2. Editorial.

217. Weatherall TJ, Conigrave JH, Conigrave KM, et al. Alcohol dependence in a community sample of Aboriginal and Torres Strait Islander Australians: harms, getting help and awareness of local treatments. *Addiction science & clinical practice* 2021; 16(1): 65.

218. Wood S and Schulman K. When Vaccine Apathy, Not Hesitancy, Drives Vaccine Disinterest. *JAMA - Journal of the American Medical Association* 2021; 325(24): 2435-2436. Note.

219. Bell R, Strom C, Shore K, et al. Engaging the American Indian community in North Carolina to assess cancer research and training opportunities. *Cancer Epidemiology Biomarkers and Prevention Conference: 14th AACR Conference on the Science of Cancer Health Disparities in Racial/Ethnic Minorities and the Medically Underserved Virtual* 2022; 31. Conference Abstract.

220. Sieving RE, Mehus C, Gewirtz O'Brien JR, et al. Correlates of Sexual and Reproductive Health Discussions During Preventive Visits: Findings From a National Sample of U.S. Adolescents. *Journal of Adolescent Health* 2022; 70(3): 421-428.

221. Sinicrope PS, Young CD, Resnicow K, et al. Lessons Learned From Beta-Testing a Facebook Group Prototype to Promote Treatment Use in the "Connecting Alaska Native People to Quit Smoking" (CAN Quit) Study. *Journal of Medical Internet Research* 2022; 24(2) (no pagination).

222. Weatherall TJ, Conigrave JH, Conigrave KM, et al. Prevalence and correlates of alcohol dependence in an Australian Aboriginal and Torres Strait Islander representative sample: Using the Grog Survey App. *Drug and alcohol review* 2022; 41(1): 125-134.

223. Wright JH, Owen J, Eells TD, et al. Effect of Computer-Assisted Cognitive Behavior Therapy vs Usual Care on Depression among Adults in Primary Care: A Randomized Clinical Trial. *JAMA Network Open* 2022; 5(2) (no pagination).

# Medline Ovid 2022.03.08

## Notes about this database search

## Actual search terms for Medline Ovid

Ovid MEDLINE(R) ALL <1946 to March 04, 2022>

1 Oceanic Ancestry Group/ or alaskan natives/ or inuits/ or Indigenous Peoples/ 16723

2 (Aboriginal or First Nation).tw. 9532

3 Torres Strait.tw. 2148

4 maori.tw. 3719

5 exp American Native Continental Ancestry Group/ 22625

6 sami.tw. 478

7 Ainu.tw. 154

8 1 or 2 or 3 or 4 or 5 or 6 or 7 41106

9 blogging/ or social media/ or electronic mail/ 16260

10 internet/ or internet access/ or "internet use"/ or internet-based intervention/ 79637

11 social networking/ or online social networking/ 5000

12 social media/ 12726

13 (smartphone or "mobile application" or App or e-health or ehealth or internet or email or Facebook or SnapChat or Instagram or Twitter or TicToc or Pinterest or youtube).tw. 124478

14 9 or 10 or 11 or 12 or 13 187656

15 consumer health information/ or health literacy/ or health promotion/ or healthy people programs/ 90775

16 weight reduction programs/ or sex education/ or smoking prevention/ 30193

17 "Quality of Life"/ 234459

18 (wellbeing or well-being or wellness).tw. 125057

19 Self Care/ 35092

20 neoplasms/ or lung diseases/ or chronic disease/ or multiple chronic conditions/ or emphysema/ or Cardiovascular Diseases/ or diabetes mellitus/ or diabetes mellitus, type 1/ or diabetes mellitus, type 2/ 1274115

21 Stress, Psychological/ 129953

22 Alcoholism/ 78130

23 "tobacco use disorder"/ 11957

24 smoking/ or smoking reduction/ or tobacco smoking/ or vaping/ or "tobacco use"/ 151690

25 Behavior, Addictive/ or addiction disorder.mp. or Substance-Related Disorders/ 110564

26 15 or 16 or 17 or 18 or 19 or 20 or 21 or 22 or 23 or 24 or 25 2087282

27 8 and 14 and 26 129

28 limit 27 to (english language and yr="2005 -Current") 126

## Link to saved search for Medline Ovid

<https://simsrad.net.ocs.mq.edu.au/login?url=http://ovidsp.ovid.com/ovidweb.cgi?T=JS&NEWS=N&PAGE=main&SHAREDSEARCHID=B5Bb9U88glC7i81M3Z9rvEAijgyLbC8swYsAXTY9tuRpGP1bDMPn7N9Rq6mn13NP>

## References found by Medline_Ovid 2022.03.08

1. Bannink L, Wells S, Broad J, et al. Web-based assessment of cardiovascular disease risk in routine primary care practice in New Zealand: the first 18,000 patients (PREDICT CVD-1). *New Zealand Medical Journal* 2006; 119: U2313.

2. Whittaker R, Bramley D, Wells S, et al. Will a web-based cardiovascular disease (CVD) risk assessment programme increase the assessment of CVD risk factors for Maori? *New Zealand Medical Journal* 2006; 119: U2077. Comparative Study

Evaluation Study

Research Support, Non-U.S. Gov't.

3. Hoffman-Goetz L and Friedman DB. A qualitative study of Canadian Aboriginal women's beliefs about "credible" cancer information on the internet. *Journal of Cancer Education* 2007; 22: 124-128. Multicenter Study

Research Support, Non-U.S. Gov't.

4. Riddell T, Jackson RT, Wells S, et al. Assessing Maori/non-Maori differences in cardiovascular disease risk and risk management in routine primary care practice using web-based clinical decision support: (PREDICT CVD-2). *New Zealand Medical Journal* 2007; 120: U2445. Research Support, Non-U.S. Gov't.

5. Wells S, Kerr A, Broad J, et al. The impact of New Zealand CVD risk chart adjustments for family history and ethnicity on eligibility for treatment (PREDICT CVD-5). *New Zealand Medical Journal* 2007; 120: U2712. Research Support, Non-U.S. Gov't.

6. Donelle L and Hoffman-Goetz L. An exploratory study of canadian aboriginal online health care forums. *Health Communication* 2008; 23: 270-281. DOI: https://dx.doi.org/10.1080/10410230802056388.

7. Case S, Jernigan V, Gardner A, et al. Content and frequency of writing on diabetes bulletin boards: does race make a difference? *Journal of Medical Internet Research* 2009; 11: e22. Research Support, N.I.H., Extramural

Research Support, Non-U.S. Gov't. DOI: https://dx.doi.org/10.2196/jmir.1153.

8. Levine BA, Turner JW, Robinson JD, et al. Communication plays a critical role in web-based monitoring. *Journal of Diabetes Science & Technology* 2009; 3: 461-467. Multicenter Study

Research Support, U.S. Gov't, Non-P.H.S.

9. Peiris DP, Joshi R, Webster RJ, et al. An electronic clinical decision support tool to assist primary care providers in cardiovascular disease risk management: development and mixed methods evaluation. *Journal of Medical Internet Research* 2009; 11: e51. Research Support, Non-U.S. Gov't. DOI: https://dx.doi.org/10.2196/jmir.1258.

10. Burhansstipanov L, Krebs LU, Seals BF, et al. Native American breast cancer survivors' physical conditions and quality of life. *Cancer* 2010; 116: 1560-1571. Research Support, N.I.H., Extramural

Research Support, Non-U.S. Gov't. DOI: https://dx.doi.org/10.1002/cncr.24924.

11. Lemelin H, Matthews D, Mattina C, et al. Climate change, wellbeing and resilience in the Weenusk First Nation at Peawanuck: the Moccasin Telegraph goes global. *Rural & Remote Health* 2010; 10: 1333.

12. Maar MA, Seymour A, Sanderson B, et al. Reaching agreement for an Aboriginal e-health research agenda: the Aboriginal Telehealth Knowledge Circle consensus method. *Rural & Remote Health* 2010; 10: 1299.

13. Macnab A, Radziminski N, Budden H, et al. Brighter Smiles Africa--translation of a Canadian community-based health-promoting school program to Uganda. *Education for Health* 2010; 23: 241. Research Support, Non-U.S. Gov't.

14. Steinfeldt JA, Foltz BD, Kaladow JK, et al. Racism in the electronic age: Role of online forums in expressing racial attitudes about American Indians. *Cultural Diversity & Ethnic Minority Psychology* 2010; 16: 362-371. DOI: https://dx.doi.org/10.1037/a0018692.

15. Goebert D, Else I, Matsu C, et al. The impact of cyberbullying on substance use and mental health in a multiethnic sample. *Maternal & Child Health Journal* 2011; 15: 1282-1286. Research Support, N.I.H., Extramural

Research Support, Non-U.S. Gov't

Research Support, U.S. Gov't, P.H.S. DOI: https://dx.doi.org/10.1007/s10995-010-0672-x.

16. Hashim MJ. Access-literacy gap in online health education for diabetes--Internet access is just one link. *Journal of Primary Health Care* 2011; 3: 333. Letter

Comment.

17. Jernigan VB and Lorig K. The internet diabetes self-management workshop for American Indians and Alaska Natives. *Health Promotion Practice* 2011; 12: 261-270. DOI: https://dx.doi.org/10.1177/1524839909335178.

18. Kay-Lambkin FJ, White A, Baker AL, et al. Assessment of function and clinical utility of alcohol and other drug web sites: an observational, qualitative study. *BMC Public Health* 2011; 11: 277. Research Support, Non-U.S. Gov't. DOI: https://dx.doi.org/10.1186/1471-2458-11-277.

19. Okamoto SK, Helm S, Delp JA, et al. A community stakeholder analysis of drug resistance strategies of rural native Hawaiian youth. *Journal of Primary Prevention* 2011; 32: 185-193. Research Support, N.I.H., Extramural

Research Support, Non-U.S. Gov't. DOI: https://dx.doi.org/10.1007/s10935-011-0247-7.

20. Reti SR, Feldman HJ and Safran C. Online access and literacy in Maori New Zealanders with diabetes. *Journal of Primary Health Care* 2011; 3: 190-191.

21. Robinson JD, Turner JW, Levine B, et al. Expanding the walls of the health care encounter: support and outcomes for patients online. *Health Communication* 2011; 26: 125-134. DOI: https://dx.doi.org/10.1080/10410236.2010.541990.

22. Rushing SC and Stephens D. Use of media technologies by Native American teens and young adults in the Pacific Northwest: exploring their utility for designing culturally appropriate technology-based health interventions. *Journal of Primary Prevention* 2011; 32: 135-145. DOI: https://dx.doi.org/10.1007/s10935-011-0242-z.

23. Burhansstipanov L, Clark RE, Watanabe-Galloway S, et al. Online evaluation programs: benefits and limitations. *Journal of Cancer Education* 2012; 27: S24-31. Research Support, N.I.H., Extramural

Research Support, Non-U.S. Gov't. DOI: https://dx.doi.org/10.1007/s13187-012-0320-9.

24. Geana MV, Greiner KA, Cully A, et al. Improving health promotion to American Indians in the midwest United States: preferred sources of health information and its use for the medical encounter. *Journal of Community Health* 2012; 37: 1253-1263. DOI: https://dx.doi.org/10.1007/s10900-012-9564-x.

25. Henderson JA, Chubak J, O'Connell J, et al. Design of a randomized controlled trial of a web-based intervention to reduce cardiovascular disease risk factors among remote reservation-dwelling American Indian adults with type 2 diabetes. *Journal of Primary Prevention* 2012; 33: 209-222. Randomized Controlled Trial

Research Support, N.I.H., Extramural. DOI: https://dx.doi.org/10.1007/s10935-012-0276-x.

26. Samuel KA, Ribisl KM and Williams RS. Internet cigarette sales and Native American sovereignty: political and public health contexts. *Journal of Public Health Policy* 2012; 33: 173-187. Research Support, Non-U.S. Gov't

Review. DOI: https://dx.doi.org/10.1057/jphp.2012.4.

27. Cueva M, Kuhnley R, Revels LJ, et al. Bridging storytelling traditions with digital technology. *International Journal of Circumpolar Health* 2013; 72. Research Support, Non-U.S. Gov't

Research Support, U.S. Gov't, P.H.S. DOI: https://dx.doi.org/10.3402/ijch.v72i0.20717.

28. Denny S, Farrant B, Cosgriff J, et al. Forgone health care among secondary school students in New Zealand. *Journal of Primary Health Care* 2013; 5: 11-18. Research Support, Non-U.S. Gov't.

29. Filippi MK, McCloskey C, Williams C, et al. Perceptions, barriers, and suggestions for creation of a tobacco and health website among American Indian/Alaska Native college students. *Journal of Community Health* 2013; 38: 486-491. Research Support, N.I.H., Extramural

Research Support, Non-U.S. Gov't. DOI: https://dx.doi.org/10.1007/s10900-012-9634-0.

30. Gould GS, McEwen A, Watters T, et al. Should anti-tobacco media messages be culturally targeted for Indigenous populations? A systematic review and narrative synthesis. *Tobacco Control* 2013; 22: e7. Meta-Analysis

Research Support, Non-U.S. Gov't

Review

Systematic Review. DOI: https://dx.doi.org/10.1136/tobaccocontrol-2012-050436.

31. Minde GT and Saeterstrand TM. What is important in the surroundings in order to extend the healthy life period? A regional study of 19 older women in a northern part of Norway. *International Journal of Circumpolar Health* 2013; 72. DOI: https://dx.doi.org/10.3402/ijch.v72i0.21189.

32. Watanabe-Galloway S, Duran T, Stimpson JP, et al. Gaps in survey data on cancer in American Indian and Alaska Native populations: examination of US population surveys, 1960-2010. *Preventing Chronic Disease* 2013; 10: E39. Comparative Study

Research Support, N.I.H., Extramural

Research Support, U.S. Gov't, P.H.S.

Review. DOI: https://dx.doi.org/10.5888/pcd10.120258.

33. Albright CL, Steffen AD, Wilkens LR, et al. Effectiveness of a 12-month randomized clinical trial to increase physical activity in multiethnic postpartum women: results from Hawaii's Na Mikimiki Project. *Preventive Medicine* 2014; 69: 214-223. Randomized Controlled Trial

Research Support, U.S. Gov't, P.H.S. DOI: https://dx.doi.org/10.1016/j.ypmed.2014.09.019.

34. Anderson G, Gleeson S, Rissel C, et al. Twitter tweets and twaddle: twittering at AHPA. National Health Promotion Conference. *Health Promotion Journal of Australia* 2014; 25: 143-146. DOI: https://dx.doi.org/10.1071/HE13112.

35. Blinkhorn F, Wallace J, Smith L, et al. Developing leaflets to give dental health advice to Aboriginal families with young children. *International Dental Journal* 2014; 64: 195-199. Research Support, Non-U.S. Gov't. DOI: https://dx.doi.org/10.1111/idj.12108.

36. Brusse C, Gardner K, McAullay D, et al. Social media and mobile apps for health promotion in Australian Indigenous populations: scoping review. *Journal of Medical Internet Research* 2014; 16: e280. Review. DOI: https://dx.doi.org/10.2196/jmir.3614.

37. Currie LM, Ronquillo C and Dick T. Access to internet in rural and remote Canada. *Studies in Health Technology & Informatics* 2014; 201: 407-412. Comparative Study.

38. Glover M, Kira A, Gentles D, et al. The WERO group stop smoking competition: main outcomes of a pre- and post- study. *BMC Public Health* 2014; 14: 599. Evaluation Study

Research Support, Non-U.S. Gov't. DOI: https://dx.doi.org/10.1186/1471-2458-14-599.

39. Hume A, Wetten A, Feeney C, et al. Remote school gardens: exploring a cost-effective and novel way to engage Australian Indigenous students in nutrition and health. *Australian & New Zealand Journal of Public Health* 2014; 38: 235-240. Research Support, Non-U.S. Gov't. DOI: https://dx.doi.org/10.1111/1753-6405.12236.

40. Miller BL and Stogner JM. Not-so-clean fun: a profile of bath salt users among a college sample in the United States. *Journal of Psychoactive Drugs* 2014; 46: 147-153. DOI: https://dx.doi.org/10.1080/02791072.2013.876520.

41. Ram FS and McNaughton W. Giving Asthma Support to Patients (GASP): a novel online asthma education, monitoring, assessment and management tool. *Journal of Primary Health Care* 2014; 6: 238-244. Research Support, Non-U.S. Gov't.

42. Redfern J, Usherwood T, Harris MF, et al. A randomised controlled trial of a consumer-focused e-health strategy for cardiovascular risk management in primary care: the Consumer Navigation of Electronic Cardiovascular Tools (CONNECT) study protocol. *BMJ Open* 2014; 4: e004523. Randomized Controlled Trial

Research Support, Non-U.S. Gov't. DOI: https://dx.doi.org/10.1136/bmjopen-2013-004523.

43. Bradford D, Hansen D and Karunanithi M. Making an APPropriate Care Program for Indigenous Cardiac Disease: Customization of an Existing Cardiac Rehabilitation Program. *Studies in Health Technology & Informatics* 2015; 216: 343-347.

44. Campbell AN, Turrigiano E, Moore M, et al. Acceptability of a web-based community reinforcement approach for substance use disorders with treatment-seeking American Indians/Alaska Natives. *Community Mental Health Journal* 2015; 51: 393-403. Clinical Trial

Research Support, N.I.H., Extramural. DOI: https://dx.doi.org/10.1007/s10597-014-9764-1.

45. Chambers RA, Rosenstock S, Neault N, et al. A Home-Visiting Diabetes Prevention and Management Program for American Indian Youth: The Together on Diabetes Trial. *Diabetes Educator* 2015; 41: 729-747. Research Support, N.I.H., Extramural

Research Support, Non-U.S. Gov't. DOI: https://dx.doi.org/10.1177/0145721715608953.

46. Cooley ME, Blonquist TM, Catalano PJ, et al. Feasibility of using algorithm-based clinical decision support for symptom assessment and management in lung cancer. *Journal of Pain & Symptom Management* 2015; 49: 13-26. Evaluation Study

Multicenter Study

Research Support, N.I.H., Extramural. DOI: https://dx.doi.org/10.1016/j.jpainsymman.2014.05.003.

47. Jones EJ, Peercy M, Woods JC, et al. Identifying postpartum intervention approaches to reduce cardiometabolic risk among American Indian women with prior gestational diabetes, Oklahoma, 2012-2013. *Preventing Chronic Disease* 2015; 12: E45. Research Support, N.I.H., Extramural

Research Support, Non-U.S. Gov't. DOI: https://dx.doi.org/10.5888/pcd12.140566.

48. Sweet M, Geia L, Dudgeon P, et al. #IHMayDay: tweeting for empowerment and social and emotional wellbeing. *Australasian Psychiatry* 2015; 23: 636-640. DOI: https://dx.doi.org/10.1177/1039856215609762.

49. Bartgis J and Albright G. Online role-play simulations with emotionally responsive avatars for the early detection of Native youth psychological distress, including depression and suicidal ideation. *American Indian & Alaska Native Mental Health Research (Online)* 2016; 23: 1-27. DOI: https://dx.doi.org/10.5820/aian.2302.2016.1.

50. Gu Y, Kennely J, Warren J, et al. Identifying eHealth Opportunities to Support Medication Adherence - Findings of a Focus Group Study. *Studies in Health Technology & Informatics* 2016; 223: 150-157.

51. Nathan S, Rawstorne P, Hayen A, et al. Examining the pathways for young people with drug and alcohol dependence: a mixed-method design to examine the role of a treatment programme. *BMJ Open* 2016; 6: e010824. Research Support, Non-U.S. Gov't. DOI: https://dx.doi.org/10.1136/bmjopen-2015-010824.

52. Povey J, Mills PP, Dingwall KM, et al. Acceptability of Mental Health Apps for Aboriginal and Torres Strait Islander Australians: A Qualitative Study. *Journal of Medical Internet Research* 2016; 18: e65. Research Support, Non-U.S. Gov't. DOI: https://dx.doi.org/10.2196/jmir.5314.

53. Young NL, Wabano MJ, Usuba K, et al. Reliability of the Aboriginal Children's Health and Well-Being Measure (ACHWM). *Springerplus* 2016; 5: 2082. DOI: https://dx.doi.org/10.1186/s40064-016-3776-y.

54. Bennett-Levy J, Singer J, DuBois S, et al. Translating E-Mental Health Into Practice: What Are the Barriers and Enablers to E-Mental Health Implementation by Aboriginal and Torres Strait Islander Health Professionals? *Journal of Medical Internet Research* 2017; 19: e1. DOI: https://dx.doi.org/10.2196/jmir.6269.

55. Coorey GM, Neubeck L, Usherwood T, et al. Implementation of a consumer-focused eHealth intervention for people with moderate-to-high cardiovascular disease risk: protocol for a mixed-methods process evaluation. *BMJ Open* 2017; 7: e014353. Research Support, Non-U.S. Gov't. DOI: https://dx.doi.org/10.1136/bmjopen-2016-014353.

56. Dotson JA, Nelson LA, Young SL, et al. Use of cell phones and computers for health promotion and tobacco cessation by American Indian college students in Montana. *Rural & Remote Health* 2017; 17: 4014.

57. Garcia A, Baethke L and Kaur JS. Lessons Learned from Native C.I.R.C.L.E., a Culturally Specific Resource. *Journal of Cancer Education* 2017; 32: 740-744. DOI: https://dx.doi.org/10.1007/s13187-016-1001-x.

58. Gibson O, Reilly R, Harfield S, et al. Web-based therapeutic interventions for assessing, managing and treating health conditions in Indigenous people: a scoping review protocol. *JBI Database Of Systematic Reviews And Implementation Reports* 2017; 15: 2487-2494. Review. DOI: https://dx.doi.org/10.11124/JBISRIR-2016-003324.

59. Janca A, Lyons Z and Gaspar J. Here and Now Aboriginal Assessment (HANAA): a follow-up survey of users. *Australasian Psychiatry* 2017; 25: 288-289. DOI: https://dx.doi.org/10.1177/1039856217700806.

60. Kwan P, Sabado-Liwag M, Lee C, et al. Development of an Online Smoking Cessation Curriculum for Pacific Islanders: A Community-Based Participatory Research Approach. *Progress in Community Health Partnerships* 2017; 11: 263-274. Research Support, N.I.H., Extramural. DOI: https://dx.doi.org/10.1353/cpr.2017.0031.

61. Macniven R, Elwell M, Ride K, et al. A snapshot of physical activity programs targeting Aboriginal and Torres Strait Islander people in Australia. *Health Promotion Journal of Australia* 2017; 28: 185-206. DOI: https://dx.doi.org/10.1071/HE16036.

62. Nguyen AB, Robinson J, O'Brien EK, et al. Racial and Ethnic Differences in Tobacco Information Seeking and Information Sources: Findings From the 2015 Health Information National Trends Survey. *Journal of Health Communication* 2017; 22: 743-752. DOI: https://dx.doi.org/10.1080/10810730.2017.1347216.

63. Tighe J, Shand F, Ridani R, et al. Ibobbly mobile health intervention for suicide prevention in Australian Indigenous youth: a pilot randomised controlled trial. *BMJ Open* 2017; 7: e013518. Randomized Controlled Trial. DOI: https://dx.doi.org/10.1136/bmjopen-2016-013518.

64. Tomayko EJ, Prince RJ, Cronin KA, et al. Healthy Children, Strong Families 2: A randomized controlled trial of a healthy lifestyle intervention for American Indian families designed using community-based approaches. *Clinical Trials* 2017; 14: 152-161. Randomized Controlled Trial. DOI: https://dx.doi.org/10.1177/1740774516685699.

65. Volkova E, Michie J, Corrigan C, et al. Effectiveness of recruitment to a smartphone-delivered nutrition intervention in New Zealand: analysis of a randomised controlled trial. *BMJ Open* 2017; 7: e016198. Randomized Controlled Trial. DOI: https://dx.doi.org/10.1136/bmjopen-2017-016198.

66. Winch S, Ahmed N, Rissel C, et al. The reach and flow of health information in two Aboriginal communities: a social network analysis. *Australian Journal of Primary Health* 2017; 23: 189-195. DOI: https://dx.doi.org/10.1071/PY16024.

67. Black KJ, Morse B, Tuitt N, et al. Beyond Content: Cultural Perspectives on Using the Internet to Deliver a Sexual Health Intervention to American Indian Youth. *Journal of Primary Prevention* 2018; 39: 59-70. Research Support, Non-U.S. Gov't. DOI: https://dx.doi.org/10.1007/s10935-017-0497-0.

68. Conner TS, McFarlane KG, Choukri M, et al. The Oxytocin Receptor Gene (OXTR) Variant rs53576 Is Not Related to Emotional Traits or States in Young Adults. *Frontiers in Psychology* 2018; 9: 2548. DOI: https://dx.doi.org/10.3389/fpsyg.2018.02548.

69. Lombard C, Brennan L, Reid M, et al. Communicating health-Optimising young adults' engagement with health messages using social media: Study protocol. *Nutrition & Dietetics* 2018; 75: 509-519. Research Support, Non-U.S. Gov't. DOI: https://dx.doi.org/10.1111/1747-0080.12448.

70. McFarlane KA, Judd J, Wapau H, et al. How primary health care staff working in rural and remote areas access skill development and expertise to support health promotion practice. *Rural & Remote Health* 2018; 18: 4413. DOI: https://dx.doi.org/10.22605/RRH4413.

71. McPhail-Bell K, Appo N, Haymes A, et al. Deadly Choices empowering Indigenous Australians through social networking sites. *Health Promotion International* 2018; 33: 770-780. DOI: https://dx.doi.org/10.1093/heapro/dax014.

72. Narcisse MR, Felix H, Long CR, et al. Frequency and predictors of health services use by Native Hawaiians and Pacific Islanders: evidence from the U.S. National Health Interview Survey. *BMC Health Services Research* 2018; 18: 575. DOI: https://dx.doi.org/10.1186/s12913-018-3368-3.

73. Pereira-Salgado A, Mader P and Boyd LM. Advance care planning, culture and religion: an environmental scan of Australian-based online resources. *Australian Health Review* 2018; 42: 152-163. Review. DOI: https://dx.doi.org/10.1071/AH16182.

74. Verbiest M, Borrell S, Dalhousie S, et al. A Co-Designed, Culturally-Tailored mHealth Tool to Support Healthy Lifestyles in Maori and Pasifika Communities in New Zealand: Protocol for a Cluster Randomized Controlled Trial. *JMIR Research Protocols* 2018; 7: e10789. DOI: https://dx.doi.org/10.2196/10789.

75. Wilkins C, Romeo JS, Rychert M, et al. Determinants of high availability of methamphetamine, cannabis, LSD and ecstasy in New Zealand: Are drug dealers promoting methamphetamine rather than cannabis? *International Journal of Drug Policy* 2018; 61: 15-22. DOI: https://dx.doi.org/10.1016/j.drugpo.2018.09.007.

76. Wong G, Glover M, McPherson M, et al. Boosting efficacy of nurse-led stop smoking interventions with a quit and win contest: pilot study results. *Contemporary Nurse* 2018; 54: 395-408. DOI: https://dx.doi.org/10.1080/10376178.2018.1539337.

77. Desrosiers A, Vine V and Kershaw T. "R U Mad?": Computerized text analysis of affect in social media relates to stress and substance use among ethnic minority emerging adult males. *Anxiety, Stress, & Coping* 2019; 32: 109-123. Research Support, N.I.H., Extramural. DOI: https://dx.doi.org/10.1080/10615806.2018.1539964.

78. Dimitropoulos Y, Holden A and Sohn W. In-school toothbrushing programs in Aboriginal communities in New South Wales, Australia: A thematic analysis of teachers' perspectives. *Community Dental Health* 2019; 36: 106-110. DOI: https://dx.doi.org/10.1922/CDH_4443Dimitropoulos05.

79. Dingwall KM, Nagel T, Hughes JT, et al. Wellbeing intervention for chronic kidney disease (WICKD): a randomised controlled trial study protocol. *BMC psychology* 2019; 7: 2. Randomized Controlled Trial. DOI: https://dx.doi.org/10.1186/s40359-018-0264-x.

80. Elman A, Etter M, Fairman K, et al. Mental health services in the Northwest Territories: a scoping review. *International Journal of Circumpolar Health* 2019; 78: 1629783. Systematic Review. DOI: https://dx.doi.org/10.1080/22423982.2019.1629783.

81. Hefler M, Kerrigan V, Freeman B, et al. Using Facebook to reduce smoking among Australian Aboriginal and Torres Strait Islander people: a participatory grounded action study. *BMC Public Health* 2019; 19: 615. DOI: https://dx.doi.org/10.1186/s12889-019-6918-7.

82. Hefler M, Kerrigan V, Henryks J, et al. Social media and health information sharing among Australian Indigenous people. *Health Promotion International* 2019; 34: 706-715. DOI: https://dx.doi.org/10.1093/heapro/day018.

83. Lee KSK, Conigrave JH, Callinan S, et al. Asking about the last four drinking occasions on a tablet computer as a way to record alcohol consumption in Aboriginal and Torres Strait Islander Australians: a validation. *Addiction Science & Clinical Practice* 2019; 14: 15. Research Support, Non-U.S. Gov't. DOI: https://dx.doi.org/10.1186/s13722-019-0148-2.

84. McCrabb S, Twyman L, Palazzi K, et al. A cross sectional survey of internet use among a highly socially disadvantaged population of tobacco smokers. *Addiction Science & Clinical Practice* 2019; 14: 38. Research Support, Non-U.S. Gov't. DOI: https://dx.doi.org/10.1186/s13722-019-0168-y.

85. McElfish PA, Rowland B, Riklon S, et al. Development and Evaluation of a Blood Glucose Monitoring YouTube Video for Marshallese Patients Using a Community-Based Participatory Research Approach. *Policy, Politics, & Nursing Practice* 2019; 20: 205-215. DOI: https://dx.doi.org/10.1177/1527154419872834.

86. Nghiem N, Leung W, Cleghorn C, et al. Mass media promotion of a smartphone smoking cessation app: modelled health and cost-saving impacts. *BMC Public Health* 2019; 19: 283. DOI: https://dx.doi.org/10.1186/s12889-019-6605-8.

87. Ni Mhurchu C, Te Morenga L, Tupai-Firestone R, et al. A co-designed mHealth programme to support healthy lifestyles in Maori and Pasifika peoples in New Zealand (OL@-OR@): a cluster-randomised controlled trial. *The Lancet Digital Health* 2019; 1: e298-e307. Randomized Controlled Trial

Research Support, Non-U.S. Gov't. DOI: https://dx.doi.org/10.1016/S2589-7500(19)30130-X.

88. Peiris D, Wright L, News M, et al. Community-Based Chronic Disease Prevention and Management for Aboriginal People in New South Wales, Australia: Mixed Methods Evaluation of the 1 Deadly Step Program. *JMIR MHealth and UHealth* 2019; 7: e14259. DOI: https://dx.doi.org/10.2196/14259.

89. Verbiest MEA, Corrigan C, Dalhousie S, et al. Using codesign to develop a culturally tailored, behavior change mHealth intervention for indigenous and other priority communities: A case study in New Zealand. *Translational Behavioral Medicine* 2019; 9: 720-736. DOI: https://dx.doi.org/10.1093/tbm/iby093.

90. Webb Hooper M, Carpenter KM and Salmon EE. Web-Based Tobacco Cessation Interventions and Digital Inequality across US Racial/Ethnic Groups. *Ethnicity & Disease* 2019; 29: 495-504. DOI: https://dx.doi.org/10.18865/ed.29.3.495.

91. Ali-Hassan H, Eloulabi R and Keethakumar A. Internet non-use among Canadian indigenous older adults: Aboriginal Peoples Survey (APS). *BMC Public Health* 2020; 20: 1554. DOI: https://dx.doi.org/10.1186/s12889-020-09659-5.

92. Anastario M, FireMoon P, Ricker A, et al. Self-reported Exposure to Sexual and Reproductive Health Information among American Indian Youth: Implications for Technology Based Intervention. *Journal of Health Communication* 2020; 25: 412-420. Research Support, N.I.H., Extramural

Research Support, Non-U.S. Gov't. DOI: https://dx.doi.org/10.1080/10810730.2020.1777599.

93. Boyd AD, Fyfe-Johnson AL, Noonan C, et al. Communication With American Indians and Alaska Natives About Cardiovascular Disease. *Preventing Chronic Disease* 2020; 17: E160. Research Support, N.I.H., Extramural. DOI: https://dx.doi.org/10.5888/pcd17.200189.

94. Callander D, Schneider JA, Radix A, et al. Longitudinal cohort of HIV-negative transgender women of colour in New York City: protocol for the TURNNT ('Trying to Understand Relationships, Networks and Neighbourhoods among Transgender women of colour') study. *BMJ Open* 2020; 10: e032876. Research Support, N.I.H., Extramural

Research Support, Non-U.S. Gov't. DOI: https://dx.doi.org/10.1136/bmjopen-2019-032876.

95. Carroll DM, Soto C, Baezconde-Garbanati L, et al. Tobacco Industry Marketing Exposure and Commercial Tobacco Product Use Disparities among American Indians and Alaska Natives. *Substance Use & Misuse* 2020; 55: 261-270. Research Support, N.I.H., Extramural

Research Support, U.S. Gov't, P.H.S. DOI: https://dx.doi.org/10.1080/10826084.2019.1664589.

96. Firestone R, Cheng S, Dalhousie S, et al. Exploring Pasifika wellbeing: findings from a large cluster randomised controlled trial of a mobile health intervention programme. *New Zealand Medical Journal* 2020; 133: 82-101. Randomized Controlled Trial.

97. Hefler M, Kerrigan V, Grunseit A, et al. Facebook-Based Social Marketing to Reduce Smoking in Australia's First Nations Communities: An Analysis of Reach, Shares, and Likes. *Journal of Medical Internet Research* 2020; 22: e16927. Research Support, Non-U.S. Gov't. DOI: https://dx.doi.org/10.2196/16927.

98. Jongbloed K, Pearce ME, Thomas V, et al. The Cedar Project - Mobile Phone Use and Acceptability of Mobile Health Among Young Indigenous People Who Have Used Drugs in British Columbia, Canada: Mixed Methods Exploratory Study. *JMIR MHealth and UHealth* 2020; 8: e16783. Research Support, Non-U.S. Gov't. DOI: https://dx.doi.org/10.2196/16783.

99. Knox M, Skan J, Benowitz NL, et al. Recruitment best practices of a cardiovascular risk reduction randomised control trial in rural Alaska Native communities. *International Journal of Circumpolar Health* 2020; 79: 1806639. Randomized Controlled Trial

Research Support, N.I.H., Extramural. DOI: https://dx.doi.org/10.1080/22423982.2020.1806639.

100. Nagel T, Sweet M, Dingwall KM, et al. Adapting wellbeing research tools for Aboriginal and Torres Strait Islander people with chronic kidney disease. *BMC Nephrology* 2020; 21: 130. Research Support, Non-U.S. Gov't. DOI: https://dx.doi.org/10.1186/s12882-020-01776-y.

101. Newby JM, O'Moore K, Tang S, et al. Acute mental health responses during the COVID-19 pandemic in Australia. *PLoS ONE [Electronic Resource]* 2020; 15: e0236562. Research Support, Non-U.S. Gov't. DOI: https://dx.doi.org/10.1371/journal.pone.0236562.

102. Povey J, Sweet M, Nagel T, et al. Drafting the Aboriginal and Islander Mental Health Initiative for Youth (AIMhi-Y) App: Results of a formative mixed methods study. *Internet Interventions* 2020; 21: 100318. DOI: https://dx.doi.org/10.1016/j.invent.2020.100318.

103. Serlachius A, Schache K, Boggiss A, et al. Coping Skills Mobile App to Support the Emotional Well-Being of Young People During the COVID-19 Pandemic: Protocol for a Mixed Methods Study. *JMIR Research Protocols* 2020; 9: e23716. DOI: https://dx.doi.org/10.2196/23716.

104. Smith JA, Merlino A, Christie B, et al. 'Dudes Are Meant to be Tough as Nails': The Complex Nexus Between Masculinities, Culture and Health Literacy From the Perspective of Young Aboriginal and Torres Strait Islander Males - Implications for Policy and Practice. *American Journal of Mens Health* 2020; 14: 1557988320936121. Research Support, Non-U.S. Gov't. DOI: https://dx.doi.org/10.1177/1557988320936121.

105. Tighe J, Shand F, McKay K, et al. Usage and Acceptability of the iBobbly App: Pilot Trial for Suicide Prevention in Aboriginal and Torres Strait Islander Youth. *JMIR Mental Health* 2020; 7: e14296. DOI: https://dx.doi.org/10.2196/14296.

106. Backholer K, Baum F, Finlay SM, et al. Australia in 2030: what is our path to health for all? *Medical Journal of Australia* 2021; 214 Suppl 8: S5-S40. Research Support, Non-U.S. Gov't. DOI: https://dx.doi.org/10.5694/mja2.51020.

107. Britt RK, Britt BC, Anderson J, et al. "Sharing Hope and Healing": A Culturally Tailored Social Media Campaign to Promote Living Kidney Donation and Transplantation Among Native Americans. *Health Promotion Practice* 2021; 22: 786-795. Research Support, N.I.H., Extramural. DOI: https://dx.doi.org/10.1177/1524839920974580.

108. Carlson B, Frazer R and Farrelly T. "That makes all the difference": Aboriginal and Torres Strait Islander health-seeking on social media. *Health Promotion Journal of Australia* 2021; 32: 523-531. DOI: https://dx.doi.org/10.1002/hpja.366.

109. D'Amico EJ, Dickerson DL, Rodriguez A, et al. Integrating traditional practices and social network visualization to prevent substance use: study protocol for a randomized controlled trial among urban Native American emerging adults. *Addiction Science & Clinical Practice* 2021; 16: 56. Clinical Trial Protocol

Research Support, N.I.H., Extramural. DOI: https://dx.doi.org/10.1186/s13722-021-00265-3.

110. Dingwall KM, Sweet M, Cass A, et al. Effectiveness of Wellbeing Intervention for Chronic Kidney Disease (WICKD): results of a randomised controlled trial. *BMC Nephrology* 2021; 22: 136. Randomized Controlled Trial

Research Support, Non-U.S. Gov't. DOI: https://dx.doi.org/10.1186/s12882-021-02344-8.

111. Hale L, Devan H, Davies C, et al. Clinical and cost-effectiveness of an online-delivered group-based pain management programme in improving pain-related disability for people with persistent pain-protocol for a non-inferiority randomised controlled trial (iSelf-help trial). *BMJ Open* 2021; 11: e046376. Clinical Trial Protocol

Research Support, Non-U.S. Gov't. DOI: https://dx.doi.org/10.1136/bmjopen-2020-046376.

112. Immanuel J, Eagleton C, Baker J, et al. Pregnancy outcomes among multi-ethnic women with different degrees of hyperglycaemia during pregnancy in an urban New Zealand population and their association with postnatal HbA1c uptake. *Australian & New Zealand Journal of Obstetrics & Gynaecology* 2021; 61: 69-77. Research Support, Non-U.S. Gov't. DOI: https://dx.doi.org/10.1111/ajo.13231.

113. Kennedy M, Kumar R, Ryan NM, et al. Codeveloping a multibehavioural mobile phone app to enhance social and emotional well-being and reduce health risks among Aboriginal and Torres Strait Islander women during preconception and pregnancy: a three-phased mixed-methods study. *BMJ Open* 2021; 11: e052545. Research Support, Non-U.S. Gov't. DOI: https://dx.doi.org/10.1136/bmjopen-2021-052545.

114. Koziol-McLain J, Wilson D, Vandal AC, et al. Evaluation of a Healthy Relationship Smartphone App With Indigenous Young People: Protocol for a Co-designed Stepped Wedge Randomized Trial. *JMIR Research Protocols* 2021; 10: e24792. DOI: https://dx.doi.org/10.2196/24792.

115. Kyoon-Achan G, Schroth RJ, Sanguins J, et al. Early childhood oral health promotion for First Nations and Metis communities and caregivers in Manitoba. *Health Promotion and Chronic Disease Prevention in Canada* 2021; 41: 14-24. DOI: https://dx.doi.org/10.24095/hpcdp.41.1.02.

116. Laws R, Love P, Hesketh KD, et al. Protocol for an Effectiveness-Implementation Hybrid Trial to Evaluate Scale up of an Evidence-Based Intervention Addressing Lifestyle Behaviours From the Start of Life: INFANT. *Frontiers in Endocrinology* 2021; 12: 717468. Clinical Trial Protocol

Research Support, Non-U.S. Gov't. DOI: https://dx.doi.org/10.3389/fendo.2021.717468.

117. Meno M, Abe J, Fukui J, et al. Telehealth amid the COVID-19 pandemic: perception among Asian, Native Hawaiian and Pacific Islander cancer patients. *Future Oncology* 2021; 17: 3077-3085. DOI: https://dx.doi.org/10.2217/fon-2021-0136.

118. Serlachius A, Boggiss A, Lim D, et al. Pilot study of a well-being app to support New Zealand young people during the COVID-19 pandemic. *Internet Interventions* 2021; 26: 100464. DOI: https://dx.doi.org/10.1016/j.invent.2021.100464.

119. Sinka V, Lopez-Vargas P, Tong A, et al. Chronic disease prevention programs offered by Aboriginal Community Controlled Health Services in New South Wales, Australia. *Australian & New Zealand Journal of Public Health* 2021; 45: 59-64. DOI: https://dx.doi.org/10.1111/1753-6405.13069.

120. Skinner J, Dimitropoulos Y, Moir R, et al. A graduate oral health therapist program to support dental service delivery and oral health promotion in Aboriginal communities in New South Wales, Australia. *Rural & Remote Health* 2021; 21: 5789. DOI: https://dx.doi.org/10.22605/RRH5789.

121. Smith JA, Merlino A, Christie B, et al. Using social media in health literacy research: A promising example involving Facebook with young Aboriginal and Torres Strait Islander males from the Top End of the Northern Territory. *Health Promotion Journal of Australia* 2021; 32 Suppl 1: 186-191. DOI: https://dx.doi.org/10.1002/hpja.421.

122. Snijder M, Stapinski L, Ward J, et al. Strong and Deadly Futures: Co-Development of a Web-Based Wellbeing and Substance Use Prevention Program for Aboriginal and Torres Strait Islander and Non-Aboriginal Adolescents. *International Journal of Environmental Research & Public Health [Electronic Resource]* 2021; 18: 23. Research Support, Non-U.S. Gov't. DOI: https://dx.doi.org/10.3390/ijerph18042176.

123. Stotz S, Brega AG, Lockhart S, et al. An online diabetes nutrition education programme for American Indian and Alaska Native adults with type 2 diabetes: perspectives from key stakeholders. *Public Health Nutrition* 2021; 24: 1449-1459. Research Support, N.I.H., Extramural

Research Support, Non-U.S. Gov't. DOI: https://dx.doi.org/10.1017/S1368980020001743.

124. Sussman S, Unger JB, Begay C, et al. Prevalence, Co-Occurrence, and Correlates of Substance and Behavioral Addictions Among American Indian Adolescents in California. *Journal of Drug Education* 2021; 50: 31-44. Research Support, Non-U.S. Gov't. DOI: https://dx.doi.org/10.1177/00472379211017038.

125. Walker R, Usher K, Jackson D, et al. Connection to... Addressing Digital Inequities in Supporting the Well-Being of Young Indigenous Australians in the Wake of COVID-19. *International Journal of Environmental Research & Public Health [Electronic Resource]* 2021; 18: 22. Research Support, Non-U.S. Gov't

Review. DOI: https://dx.doi.org/10.3390/ijerph18042141.

126. Weatherall TJ, Conigrave JH, Conigrave KM, et al. Alcohol dependence in a community sample of Aboriginal and Torres Strait Islander Australians: harms, getting help and awareness of local treatments. *Addiction Science & Clinical Practice* 2021; 16: 65. Research Support, Non-U.S. Gov't. DOI: https://dx.doi.org/10.1186/s13722-021-00274-2.

# Informit 2022.03.14

## Notes about this database search

This required the most tedious process of exporting each citation 1 by 1 to endnote.

This database uses stemming automatically unless you turn it off

3 data bases searched.

- Health collection
- Indigenous collection
- New Zealand collection

## Actual search terms for Informit

Indigenous OR aboriginal OR ('Torres Strait'~1) OR ('alaskan natives'~1) OR inuits OR ('American natives'~1) OR maori OR sami OR Ainu OR ('First Nation'~1)

AND

Blog OR ('social media'~1) OR ('electronic mail'~1) OR email OR ('social network'~1) OR internet OR ('internet access'~1) OR ('internet use'~1) OR ('internet-based intervention'~1) OR smartphone OR ('e-health'~1) OR (‘ehealth’~1) OR ('mobile application'~1) OR App OR Facebook OR SnapChat OR Instagram OR Twitter OR TicToc OR Pinterest OR youtube

AND

('consumer health'~1) OR ('health literacy'~1) OR ('health promotion'~1) OR ('weight reduction'~1) OR (‘sex education’~1) OR (‘psychological stress’~1) OR (‘Quality of Life’~1) OR wellbeing OR (‘well-being’~1) OR wellness OR (“Self Care’~1) OR disease OR (‘cardiovascular disease’~1) OR (‘malignant neoplasm’~1) OR neoplasm OR (‘lung disease’~1) OR (‘respiratory tract disease’~1) OR (‘chronic disease’~1) OR (‘chronic condition’~1) OR (‘multiple chronic conditions’~1) OR emphysema OR diabetes OR alcoholism OR smoking OR (‘smoking reduction’~1) OR vaping OR (‘tobacco use’~1) OR (‘smoking prevention’~1) OR addiction OR (‘addiction disorder’~1)

[All Fields: indigenous OR All Fields: aboriginal OR All Fields: 'torres strait'~ OR All Fields: 'alaskan natives'~ OR All Fields: inuits OR All Fields: 'american natives'~ OR All Fields: maori OR All Fields: sami OR All Fields: ainu OR All Fields: 'first nation'~] AND [All Fields: blog OR All Fields: 'social media'~ OR All Fields: 'electronic mail'~ OR All Fields: email OR All Fields: 'social network'~ OR All Fields: internet OR All Fields: 'internet access'~ OR All Fields: 'internet use'~ OR All Fields: 'internet-based intervention'~ OR All Fields: smartphone OR All Fields: 'e-health'~ OR All Fields: ‘ehealth’~ OR All Fields: 'mobile application'~ OR All Fields: app OR All Fields: facebook OR All Fields: snapchat OR All Fields: instagram OR All Fields: twitter OR All Fields: tictoc OR All Fields: pinterest OR All Fields: youtube] AND [All Fields: 'consumer health'~ OR All Fields: 'health literacy'~ OR All Fields: 'health promotion'~ OR All Fields: 'weight reduction'~ OR All Fields: ‘sex education’~ OR All Fields: ‘psychological stress’~ OR All Fields: ‘quality of life’~ OR All Fields: wellbeing OR All Fields: ‘well-being’~ OR All Fields: wellness OR All Fields: “self care’~ OR All Fields: disease OR All Fields: ‘cardiovascular disease’~ OR All Fields: ‘malignant neoplasm’~ OR All Fields: neoplasm OR All Fields: ‘lung disease’~ OR All Fields: ‘respiratory tract disease’~ OR All Fields: ‘chronic disease’~ OR All Fields: ‘chronic condition’~ OR All Fields: ‘multiple chronic conditions’~ OR All Fields: emphysema OR All Fields: diabetes OR All Fields: alcoholism OR All Fields: smoking OR All Fields: ‘smoking reduction’~ OR All Fields: vaping OR All Fields: ‘tobacco use’~ OR All Fields: ‘smoking prevention’~ OR All Fields: addiction OR All Fields: ‘addiction disorder’~] AND Language: English AND Databases: Health Collection AND Databases: New Zealand Collection AND Databases: Indigenous Collection AND Resource Type: Other Journal Article AND Resource Type: Journal AND Publication Date: (01/01/2005 TO 31/12/2022) AND Resource Type: Conference OR Report OR Journal AND Language: English

## Link to saved search for Informit

<https://search.informit.org/search/saved>

## References found by Informit 2022.03.07

1. Genevieve RC, Paula F, Michael PA, et al. Indigenous standpoint theory as a theoretical framework for decolonizing social science health research with American Indian communities. AlterNative. DOI: 10.3316/informit.278389556005084.

2. Mapuana CKA, Kuaiwi Laka M, Samantha K, et al. The study of life and food systems for Native Hawaiians based on their environment. AlterNative. DOI: 10.3316/informit.278445454918859.

3. Helen B, Trevor I, Kelsey T, et al. Amxsame’ su sa “Nawalakw (the supernatural spirit wraps around us): impacts of Kwakwaka”wakw regalia making on identity, wellness and belonging in ’Yalis, British Columbia. AlterNative. DOI: 10.3316/informit.278501353832633.

4. Laurie-Ann L, Casadaya M, Shaun A, et al. Indigenizing forum theatre through a strength-based approach. AlterNative. DOI: 10.3316/informit.278538619775149.

5. Juan Jaime Loera G. Indigenous people’s self-determination in the context of covid-19 in Northern Mexico. AlterNative. DOI: 10.3316/informit.278575885717666.

6. Rusaslina I, Zanisah M, Anthony W-H, et al. Indigenous resilience and the COVID-19 response: A situation report on the Orang Asli in Peninsular Malaysia. AlterNative. DOI: 10.3316/INFORMIT.096345426812470.

7. Marama M-L, Hilary L and Tia D. Ko nga kaumatua nga poupou o to ratou ao: Kaumatua and kuia, the pillars of our understanding. AlterNative. DOI: 10.3316/informit.931890809697147.

8. Grace K-A, Robert S, Daniella D, et al. Healthy Smile, Happy Child: Partnering with Manitoba First Nations and Metis communities for better early childhood oral health. AlterNative. DOI: 10.3316/informit.931946708610922.

9. Crista W, Ibis MA and Natalia S. Designing a deep intercultural curriculum in higher education: Co-constructing knowledge with Indigenous women. AlterNative. DOI: 10.3316/informit.932095772380988.

10. Jerry Degollacion I. “Bungkalan” and the Manobo- Pulangihon tribe’s resistance to corporate land-grab in Bukidnon, Mindanao. AlterNative. DOI: 10.3316/informit.788864122319204.

11. Sacha M, Helen L and Catherine S. An indigenous self-determination social movement response to covid-19. AlterNative. DOI: 10.3316/informit.579354993957665.

12. Renee M, Janet S and Cheryllee B. “It’s not like I’m more indigenous there and I’m less indigenous here.”: Urban Metis women’s identity and access to health and social services in Toronto, Canada. AlterNative. DOI: 10.3316/informit.579448158813956.

13. Jeff C, Robynne E, Renee M, et al. Everyday indigenous resurgence during covid-19: A social media situation report. AlterNative. DOI: 10.3316/informit.579578589612763.

14. Onowa M, Kari ABC and Kahtehronni Iris S. Indigenous language learning impacts, challenges and opportunities in covid-19 times. AlterNative. DOI: 10.3316/informit.579615855555279.

15. Onowa M, Andrea S and William C. i-kiyohkatoyahk (we visit): Adapting nehiyawewin/nihithawiwin (Cree) language learning to the COVID-19 reality. AlterNative. DOI: 10.3316/informit.579634488526538.

16. Andre M, Suzanne P and Simon Justin A. Kia whakatomuri te haere whakamua: Engaging Maori rural communities in health and social service care. AlterNative. DOI: 10.3316/informit.446427377001666.

17. Mercy B. Perspectives of my lived experiences for addressing suicides among Aboriginal communities in the North Queensland tropics. AlterNative. DOI: 10.3316/informit.446446009972924.

18. Reena T and John Richard S. Trauma and healing at Western Australia’s former native missions. AlterNative. DOI: 10.3316/informit.446520541857957.

19. Anna KR, Shemana C, Jacquie K, et al. Seeing the unseen: Evidence of kaupapa Maori health interventions. AlterNative. DOI: 10.3316/informit.234700924594739.

20. Shawn W, Anna Lydia S, Harald G, et al. Double perspective narrating time, life and health. AlterNative. DOI: 10.3316/informit.234719557565997.

21. Molly G, Reremoana T, Rosalina R, et al. Moe Kitenga: A qualitative study of perceptions of infant and child sleep practices among Maori whanau. AlterNative. DOI: 10.3316/informit.234756823508514.

22. Esther W, Sarah-Jane P, Emma W, et al. Indigenous voices on measuring and valuing health states. AlterNative. DOI: 10.3316/informit.094133789422878.

23. Rachel W, Chellie S, Ella H, et al. A culturally derived framework of values-driven transformation in Maori economies of well-being (Nga hono ohanga oranga). AlterNative. DOI: 10.3316/informit.094226954279169.

24. Anna Corrigal F, Janice Cindy G and Leah Marie D. Metis women gathering: Visiting together and voicing wellness for ourselves. AlterNative. DOI: 10.3316/informit.094487815876784.

25. Margaret K. Indigenous evaluation frameworks: Can the convention for the safeguarding of the intangible cultural heritage be a guide for recognizing indigenous scholarship within tenure and promotion standards? AlterNative. DOI: 10.3316/informit.953305779862953.

26. Pirjo Kristiina V. Ancestors’ times and protection of Amazonian Indigenous biocultural heritage. AlterNative. DOI: 10.3316/informit.953454843633019.

27. Merete S and Else Malfrid B. Young Sami men on the move: Actors, activities, and aims for the future. AlterNative. DOI: 10.3316/informit.953566641460568.

28. Renee M, Janet S, Cheryllee B, et al. “I would prefer to have my health care provided over a cup of tea any day”: Recommendations by urban metis women to improve access to health and social services in toronto for the metis community. AlterNative. DOI: 10.3316/informit.702468721250726.

29. Jacqueline O-C, Melissa Medina P and Markus M. Indigenous Amazonians on air: “Shipibo - Konibo” radio broadcasters and their social influence in Peru. AlterNative. DOI: 10.3316/informit.583907125134800.

30. Teah C, Helen Moewaka B and Tim M. Health literacy in action: Kaupapa Maori evaluation of a cardiovascular disease medications health literacy intervention. AlterNative. DOI: 10.3316/informit.583925758106058.

31. Jessica Saniguq U. For the love of our children: An indigenous connectedness framework. AlterNative. DOI: 10.3316/informit.583963024048574.

32. Julie G, Melody Morton N, Kathryn G, et al. The rationale for developing a programme of services by and for indigenous men in a first nations community. AlterNative. DOI: 10.3316/informit.584037555933607.

33. Ihirangi H, David R, Boyd S, et al. Systems thinking and indigenous systems: Native contributions to obesity prevention. AlterNative. DOI: 10.3316/informit.299158058366987.

34. Sweeney W and Kenneth ER. The sharing of indigenous knowledge through academic means by implementing self-reflection and story. AlterNative. DOI: 10.3316/informit.299251223223278.

35. Laura C, Dave C, Sabina I, et al. The Turtle Lodge: Sustainable self-determination in practice. AlterNative. DOI: 10.3316/informit.299325755108311.

36. Sarah H, Christine S and Margaret F. It’s all about Whanaungatanga: Alcohol use and older Maori in Aotearoa. AlterNative. DOI: 10.3316/informit.298654968143016.

37. Reremoana T, Mele T, Karen T, et al. Pacific university graduates in New Zealand: What helps and hinders completion. AlterNative. DOI: 10.3316/informit.296623974275872.

38. Annah Anikie M and Dipotso G. Child rearing practices of the San communities in Botswana: Potential lessons for educators. AlterNative. DOI: 10.3316/informit.296642607247130.

39. Belinda B, Helen Moewaka B and Tim M. Conceptualising historical privilege: The flip side of historical trauma, a brief examination. AlterNative. DOI: 10.3316/informit.298953095683147.

40. Kendall S. A consultation journey: Developing a Kaupapa Maori research methodology to explore Maori whanau experiences of harm and loss around birth. AlterNative. DOI: 10.3316/informit.298971728654405.

41. Lisa Te M, Crystal P, Callie C, et al. Co-designing an mHealth tool in the New Zealand Maori community with a “Kaupapa Maori” approach. AlterNative. DOI: 10.3316/informit.299064893510696.

42. Pita K and Neville R. Maori men, relationships, and everyday practices: Towards broadening domestic violence research. AlterNative. DOI: 10.3316/informit.296754405074680.

43. Sarah de L and Margo G. Turning a new page: Cultural safety, critical creative literary interventions, truth and reconciliation, and the crisis of child welfare. AlterNative. DOI: 10.3316/informit.297667420666331.

44. Shaun A, Jason M, Maui H, et al. Whakatipu rawa ma nga uri whakatipu: optimising the “Maori” in Maori economic development. AlterNative. DOI: 10.3316/informit.297015266672294.

45. Melanie S-W and Belinda B. Gi-gikinomaage-min (we are all teachers): Using history to give voice to urban native American communities. AlterNative. DOI: 10.3316/informit.297052532614811.

46. Reremoana T, Megan G, Karen T, et al. Maori University success: What helps and hinders qualification completion. AlterNative. DOI: 10.3316/informit.297220229356135.

47. Tiffany SL. Native American studies: A place of community. AlterNative. DOI: 10.3316/informit.297499723925008.

48. Tarapuhi B-B and Catherine T. Indigenizing military citizenship: Remaking state responsibility and care towards Maori veterans’ health through the treaty of Waitangi. AlterNative. DOI: 10.3316/informit.297555622838782.

49. Rachel F, Lee-Anne T, Jennifer R, et al. PATU: Fighting fit, fighting fat! The Hinu Wero approach. AlterNative. DOI: 10.3316/informit.326168606100388.

50. Paerau W. A cause for nervousness: The proposed Maori land reforms in New Zealand. AlterNative. DOI: 10.3316/informit.469567952903495.

51. Karen A, Jennifer B, Claire P, et al. Experiences of urban Australian Indigenous peer mentors in a non-communicable disease prevention program. AlterNative. DOI: 10.3316/informit.469903346386143.

52. Te Kahu R. When the media asks about the Maori. AlterNative. DOI: 10.3316/informit.523361340925918.

53. Bronwyn C. Striking the right chord: Indigenous people and the love of country. AlterNative. DOI: 10.3316/informit.523771266293599.

54. Hilary NW. Where wounded knee meets wounded knees: Skate parks and Native American youth. AlterNative. DOI: 10.3316/informit.523827165207373.

55. Jeff B. “I’m just as Indian standing before you with no feathers popping out of my head”: Critiquing Indigenous performativity in the YouTube performances of the 1491s. AlterNative. DOI: 10.3316/informit.524050760862472.

56. Ricardo G. Embodying an imagined other through rebellion, resistance and joy: Mardi Gras Indians and Black indigeneity. AlterNative. DOI: 10.3316/informit.524143925718763.

57. Morna M. Development or devastation?: Epistemologies of Mayan women’s resistance to an open-pit goldmine in Guatemala. AlterNative. DOI: 10.3316/informit.894107255774797.

58. Tiopira M. Taua nakahi nui: Maori, liquor and land loss in the 19th century. AlterNative. DOI: 10.3316/informit.242046427059749.

59. Juanita S, Stacey L, Kay D, et al. Who are the experts here?: Recognition of Aboriginal women and community workers in research and beyond. AlterNative. DOI: 10.3316/informit.242530884312462.

60. Erica N. Rooted in the land: Taino identity, oral history and stories of reclamation in contemporary contexts. AlterNative. DOI: 10.3316/informit.666393714493996.

61. Janet J, Audrey G, Yvonne B, et al. Shared decision making with Aboriginal women facing health decisions: A qualitative study identifying needs, supports, and barriers. AlterNative. DOI: 10.3316/informit.666449613407770.

62. Cash A, Vanessa de Oliveira A, Garrick C, et al. Beyond epistemic provincialism: De-provincializing indigenous resistance. AlterNative. DOI: 10.3316/informit.571278082411019.

63. Leonie P, Paul R, Cherryl S, et al. Positioning historical trauma theory within Aotearoa New Zealand. AlterNative. DOI: 10.3316/informit.571464412123601.

64. Mere K, Corinthia AK, Betty M, et al. E kore e ngaro nga kakano i ruia mai i Rangiatea: The language and culture from Rangiatea will never be lost in health and ageing research. AlterNative. DOI: 10.3316/informit.571650741836183.

65. Loriene R. Commentary: Leading a fulfilled life as an indigenous academic. AlterNative. DOI: 10.3316/informit.571762539663732.

66. John B. Perceptions of landscape and the interplay between rainfall and vegetation. AlterNative. DOI: 10.3316/informit.808457173090986.

67. Deborah M. Lessons for collaboration involving traditional knowledge and environmental governance in Ontario, Canada. AlterNative. DOI: 10.3316/informit.816748845300885.

68. Lone Elizabeth K, Philip B and Onalenna Tiny K. Transforming literacy research for the indigenous San of Botswana: Adopting appropriate research methods. AlterNative. DOI: 10.3316/informit.191232740037782.

69. Ruth M-A and Ina KB. Songlines and touchstones: A study of perinatal health and culture in Greenland. AlterNative. DOI: 10.3316/informit.191419069750364.

70. Greg B. Rum and corn pipes. AlterNative. DOI: 10.3316/informit.202911501227395.

71. Melissa W, Bronwyn F, Kyly M, et al. Perspectives on a decolonizing approach to research about Indigenous women’s health: The Indigenous Women’s Wellness Study. AlterNative. DOI: 10.3316/informit.534358905757535.

72. Jacquie K, Veronique G, Erena K, et al. A whanau ora journey of Maori men with chronic illness: A Te Korowai analysis. AlterNative. DOI: 10.3316/informit.536017240199515.

73. Erica N. “Chattling the indigenous other”: A historical examination of the enslavement of aboriginal peoples in Canada. AlterNative. DOI: 10.3316/informit.006380718375314.

74. Galumalemana AH. “To let die”: The state of the Samoan language in New Zealand. AlterNative. DOI: 10.3316/informit.429479596735262.

75. Derek P and Kay D. Isomorphism and organizational culture: A First Nation’s housing initiative. AlterNative. DOI: 10.3316/informit.740072594172536.

76. Kisiku Sa’qawei Paq’tism Randolph B. From little things big things grow, from big things little things manifest: An indigenous human ecology discussing issues of conflict, peace and relational sustainability. AlterNative. DOI: 10.3316/informit.740165759028827.

77. Litea M-S and Wheturangi W-T. Global declaration and village discourses: Social policy and indigenous wellbeing. AlterNative. DOI: 10.3316/informit.740203024971344.

78. Karen L and Audrey RG. An analysis of the evacuation policy for pregnant First Nations women in Canada. AlterNative. DOI: 10.3316/informit.740258923885118.

79. Amanda G, Belinda B, Tim M, et al. Reading News about Maori: Responses from Non-maori Media Audiences. AlterNative. DOI: 10.3316/informit.042702065547681.

80. Lisa C. Whanau ora: Hauora Maori models for kotahitanga/co-operative co-existence with non-Maori. AlterNative. DOI: 10.3316/informit.704973762977883.

81. Christine MK. Midwives, women and their families: A Maori gaze: Towards partnerships for maternity care in Aotearoa New Zealand. AlterNative. DOI: 10.3316/informit.705029661891658.

82. Paul W. The Treaty and “treating” Maori health: Politics, policy and partnership. AlterNative. DOI: 10.3316/informit.705066927834174.

83. Darrin H, Linda WN and Mohi R. Maori men and the indirect procurement and sharing of prescription medications. AlterNative. DOI: 10.3316/informit.705085560805432.

84. Rawiri T, Annemarie G and Rachael T. Homai to hono: Connecting customary, conventional and spiritual healing practices within a rural-based Maori community. AlterNative. DOI: 10.3316/informit.705141459719207.

85. Meaola A-T. To Each a Language: Addressing the Challenges of Language and Cultural Loss for Samoans. AlterNative. DOI: 10.3316/informit.480079486081543.

86. Sione Ti. Kafataha: Strategies to Preserve Pacific Languages. AlterNative. DOI: 10.3316/informit.480303081736642.

87. Tagaloatele Peggy F-D. “He”s Won, But He’s Lost It’: Applying a Samoa Gender Lens to Education Outcomes. AlterNative. DOI: 10.3316/informit.480321714707900.

88. Andrejs K, Dan Roronhiakewen L and Kelly Y. Re-indigenizing Curriculum: An Eco-hermeneutic Approach to Learning. AlterNative. DOI: 10.3316/informit.493830618870095.

# Screening History

## 1164 total retrieved

Notes about duplicate removal process

359 duplicates removed (endnote removed 343; Rayyan removed an additional 16) = 805 papers to screen.

## 805 papers screened via title only

Excluded 687 based on title (Rayyan. Review: 2022:03:16 What is the evidence for how First Nations women use digital health technology to enhance health of their communities) = 118

## 118 Papers reviewed abstract or full text

All papers reviewed listed below (115 were excluded with reasons listed in (Rayyan Review 2022-03-22: 2022-03-22 First Nations Women Tech Full article screen (118 articles)

1. Wright JH, Owen J, Eells TD, Antle B, Bishop LB, Girdler R, et al. Effect of Computer-Assisted Cognitive Behavior Therapy vs Usual Care on Depression among Adults in Primary Care: A Randomized Clinical Trial. JAMA Network Open.5.

2. Wong G, Glover M, McPherson M, Garrett N, McLeod S. Boosting efficacy of nurse-led stop smoking interventions with a quit and win contest: pilot study results. Contemporary Nurse.54(4):395-408.

3. Winch S, Ahmed N, Rissel C, Maxwell M, Coutts J, Lucas K. The reach and flow of health information in two Aboriginal communities: a social network analysis. Australian Journal of Primary Health.23(2):189-95.

4. Whitacre B, Brooks L. Do broadband adoption rates impact a community's health? Behaviour & Information Technology. 2014;33(7):767-79.

5. Webb Hooper M, Carpenter KM, Salmon EE. Web-Based Tobacco Cessation Interventions and Digital Inequality across US Racial/Ethnic Groups. Ethnicity & Disease. 2019;29(3):495-504.

6. Walsh L, Hyett N, Juniper N, Li C, Rodier S, Hill S. The use of social media as a tool for stakeholder engagement in health service design and quality improvement: A scoping review. DIGITAL HEALTH.7.

7. Walker T, Palermo C, Klassen K. Considering the Impact of Social Media on Contemporary Improvement of Australian Aboriginal Health: Scoping Review. JMIR PUBLIC HEALTH AND SURVEILLANCE.5(1):64-71.

8. Verbiest MEA, Corrigan C, Dalhousie S, Firestone R, Funaki T, Goodwin D, et al. Using codesign to develop a culturally tailored, behavior change mHealth intervention for indigenous and other priority communities: A case study in New Zealand. Translational Behavioral Medicine.9(4):720-36.

9. Venter A, Burns R, Hefford M, Ehrenberg N. Results of a telehealth-enabled chronic care management service to support people with long-term conditions at home. Journal of Telemedicine & Telecare. 2012;18(3):172-5.

10. Tran JH, Mouttapa M, Ichinose TY, Pang JK, Ueda D, Tanjasiri SP. Sources of information that promote breast and cervical cancer knowledge and screening among native Hawaiians in Southern California. J Cancer Educ.25(4):588-94.

11. Tonkin E, Jeffs L, Wycherley TP, Maher C, Smith R, Hart J, et al. A Smartphone App to Reduce Sugar-Sweetened Beverage Consumption Among Young Adults in Australian Remote Indigenous Communities: Design, Formative Evaluation and User-Testing. JMIR MHEALTH AND UHEALTH.5(12).

12. Tobe SW, Yeates K, Campbell NRC, Maar MA, Perkins N, Liu PP, et al. Diagnosing hypertension in Indigenous Canadians (DREAM-GLOBAL): A randomized controlled trial to compare the effectiveness of short message service messaging for management of hypertension: Main results. Journal of Clinical Hypertension. 2019;21(1):29-36.

13. Titov N, Dear BF, Staples LG, Bennett-Levy J, Klein B, Rapee RM, et al. The first 30 months of the MindSpot Clinic: Evaluation of a national e-mental health service against project objectives. Aust N Z J Psychiatry.51(12):1227-39.

14. Sweet MA. Social media: New links for indigenous health. Medical Journal of Australia. 2013;199(1):18.

15. Sweet M, Geia L, Dudgeon P, McCallum K. #IHMayDay: tweeting for empowerment and social and emotional wellbeing. Australasian Psychiatry.23(6):636-40.

16. Stotz SA, Brega AG, Moore KR. Online diabetes nutrition education for american indian and alaska native adults with type 2 diabetes: Stakeholder perspectives. Diabetes Conference: 79th Scientific Sessions of the American Diabetes Association, ADA. 2019;68.

17. Stewart J, Krows ML, Schaafsma TT, Heller KB, Brown ER, Boonyaratanakornkit J, et al. Comparison of Racial, Ethnic, and Geographic Location Diversity of Participants Enrolled in Clinic-Based vs 2 Remote COVID-19 Clinical Trials. JAMA Netw Open.5(2):e2148325.

18. Snijder M, Stapinski L, Ward J, Lees B, Chapman C, Champion K, et al. Strong and deadly futures: Co-development of a web-based wellbeing and substance use prevention program for aboriginal and torres strait islander and non-aboriginal adolescents. International Journal of Environmental Research and Public Health.18:1-25.

19. Sinicrope PS, Young CD, Resnicow K, Merritt ZT, McConnell CR, Hughes CA, et al. Lessons Learned From Beta-Testing a Facebook Group Prototype to Promote Treatment Use in the “Connecting Alaska Native People to Quit Smoking” (CAN Quit) Study. Journal of Medical Internet Research. 2022;24(2).

20. Shibasaki S, Gardner K, Sibthorpe B. Using knowledge translation to craft “sticky” social media health messages that provoke interest, raise awareness, impart knowledge, and inspire change. JMIR mHealth and uHealth. 2016;4(4).

21. Shaw RJ, Ferranti J. Patient-provider internet portals -- patient outcomes and use. CIN: Computers, Informatics, Nursing. 2011;29(12):714-20.

22. Santiago-Torres M, Mull KE, Sullivan BM, Kwon DM, Nez Henderson P, Nelson LA, et al. Efficacy and Utilization of Smartphone Applications for Smoking Cessation Among American Indians and Alaska Natives: Results From the iCanQuit Trial. Nicotine & tobacco research : official journal of the Society for Research on Nicotine and Tobacco. 2022;24(4):544-54.

23. Sacha M, Helen L, Catherine S. An indigenous self-determination social movement response to covid-19. AlterNative.

24. Roubidoux MA, Hilmes M, Abate S, Burhansstipanov L, Trapp MA. Development of computer games to teach breast cancer screening to Native American patients and their healthcare providers. Journal of Women's Imaging. 2005;7(2):77-95.

25. Rosenbaum DL, Piers AD, Schumacher LM, Kase CA, Butryn ML. Racial and ethnic minority enrollment in randomized clinical trials of behavioural weight loss utilizing technology: a systematic review. Obesity Reviews.18:808-17.

26. Robinson RF, Dillard DA, Hiratsuka VY, Smith JJ, Tierney S, Avey JP, et al. Formative Evaluation to Assess Communication Technology Access and Health Communication Preferences of Alaska Native People. INTERNATIONAL JOURNAL OF INDIGENOUS HEALTH.10(2):88-101.

27. Robinson JD, Warisse Turner J, Levine B, Tian Y. Expanding the walls of the health care encounter: Support and outcomes for patients online. Health Communication.26:125-34.

28. Robertson C, Kattelmann K, Ren C. Control of type 2 diabetes mellitus using interactive internet-based support on a Northern Plains Indian reservation: a pilot study. Topics in Clinical Nutrition. 2007;22(2):185-93.

29. Rieke JL. Linking Native Americans to quality health information on the Internet: a tribal college library collaboration. Journal of Consumer Health on the Internet. 2005;9(2):27-42.

30. Reti SR, Feldman HJ, Safran C. Online access and literacy in Maori New Zealanders with diabetes. Journal of Primary Health Care. 2011;3(3):190-1.

31. Reena T, John Richard S. Trauma and healing at Western Australia’s former native missions. AlterNative.

32. Ram FS, McNaughton W. Giving Asthma Support to Patients (GASP): a novel online asthma education, monitoring, assessment and management tool. Journal of Primary Health Care.6(3):238-44.

33. Rachel F, Lee-Anne T, Jennifer R, Maria P, Donna F, Sue S-C. PATU: Fighting fit, fighting fat! The Hinu Wero approach. AlterNative.

34. Power JM, Braun KL, Bersamin A. Exploring the Potential for Technology-Based Nutrition Education Among WIC Recipients in Remote Alaska Native Communities. Journal of Nutrition Education & Behavior. 2017;49:S186-S91.e1.

35. Povey J, Mills PP, Dingwall KM, Lowell A, Singer J, Rotumah D, et al. Acceptability of Mental Health Apps for Aboriginal and Torres Strait Islander Australians: A Qualitative Study. Journal of Medical Internet Research.18(3):e65.

36. Peiris D, Wright L, News M, Rogers K, Redfern J, Chow C, et al. A smartphone app to assist smoking cessation among aboriginal Australians: Findings from a pilot randomized controlled trial. JMIR mHealth and uHealth. 2019;7(4).

37. Peiris D, Wright L, News M, Corcoran K. Community-Based Chronic Disease Prevention and Management for Aboriginal People in New South Wales, Australia: Mixed Methods Evaluation of the 1 Deadly Step Program. JMIR MHealth and UHealth.7(10):e14259.

38. Peake RM, Jackson D, Lea J, Usher K. Investigating the processes used to develop and evaluate the effectiveness of health education resources for adult Indigenous people: A literature review. Contemporary Nurse: A Journal for the Australian Nursing Profession. 2019;55(4):421-49.

39. Ni Mhurchu C, Te Morenga L, Tupai-Firestone R, Grey J, Jiang Y, Jull A, et al. A co-designed mHealth programme to support healthy lifestyles in Maori and Pasifika peoples in New Zealand (OL@-OR@): a cluster-randomised controlled trial. The Lancet Digital Health.1(6):e298-e307.

40. Nguyen AB, Robinson J, O'Brien EK, Zhao X. Racial and Ethnic Differences in Tobacco Information Seeking and Information Sources: Findings From the 2015 Health Information National Trends Survey. Journal of Health Communication.22(9):743-52.

41. Nghiem N, Leung W, Cleghorn C, Blakely T, Wilson N. Mass media promotion of a smartphone smoking cessation app: modelled health and cost-saving impacts. BMC Public Health.19(1):283.

42. Nagel T, Sweet M, Dingwall KM, Puszka S, Hughes JT, Kavanagh DJ, et al. Adapting wellbeing research tools for Aboriginal and Torres Strait Islander people with chronic kidney disease. BMC Nephrology.21(1):130.

43. Morey OT. Digital disparities: the persistent digital divide as related to health information access on the Internet. Journal of Consumer Health on the Internet. 2007;11(4):23-41.

44. Mesch G, Mano R, Tsamir J. Minority status and health information search: A test of the social diversification hypothesis. Social Science & Medicine.75(5):854-8.

45. Melissa W, Bronwyn F, Kyly M, Debra A. Perspectives on a decolonizing approach to research about Indigenous women’s health: The Indigenous Women’s Wellness Study. AlterNative.

46. McPhail-Bell K, Appo N, Haymes A, Bond C, Brough M, Fredericks B. Deadly Choices empowering Indigenous Australians through social networking sites. Health Promotion International.33(5):770-80.

47. McElfish PA, Rowl, B, Riklon S, Aitaoto N, Sinclair KA, et al. Development and Evaluation of a Blood Glucose Monitoring YouTube Video for Marshallese Patients Using a Community-Based Participatory Research Approach. Policy, Politics, & Nursing Practice.20(4):205-15.

48. McCrabb S, Twyman L, Palazzi K, Guillaumier A, Paul C, Bonevski B. A cross sectional survey of internet use among a highly socially disadvantaged population of tobacco smokers. Addiction Science & Clinical Practice.14(1):38.

49. Mathieson K, Leafman JS, Horton MB. Access to Digital Communication Technology and Perceptions of Telemedicine for Patient Education among American Indian Patients with Diabetes. Journal of Health Care for the Poor & Underserved. 2017;28(4):1522-36.

50. Maar MA, Seymour A, erson B, Boesch L. Reaching agreement for an Aboriginal e-health research agenda: the Aboriginal Telehealth Knowledge Circle consensus method. Rural & Remote Health. 2010;10(1):10p-p.

51. Lorig K, Ritter PL, Laurent DD, Plant K, Green M, Jernigan VB, et al. Online diabetes self-management program: a randomized study. Diabetes Care.33(6):1275-81.

52. Lombard C, Brennan L, Reid M, Klassen KM, Palermo C, Walker T, et al. Communicating health-Optimising young adults' engagement with health messages using social media: Study protocol. Nutrition & Dietetics.75(5):509-19.

53. Lofti-Jam KL, O'Reilly CL, Feng CS, Wakefield MA, Durkin S, Broun KH. Increasing bowel cancer screening participation: integrating population-wide, primary care and more targeted approaches. Public Health Res Pract.29(2).

54. Lisa Te M, Crystal P, Callie C, Leonie M, Rangimarie M, Debbie G, et al. Co-designing an mHealth tool in the New Zealand Maori community with a “Kaupapa Maori” approach. AlterNative.

55. Li J, Brar A. The use and impact of digital technologies for and on the mental health and wellbeing of Indigenous people: A systematic review of empirical studies. Computers in Human Behavior. 2022;126:N.PAG-N.PAG.

56. Levine BA, Turner JW, Robinson JD, Angelus P, Hu TM. Communication plays a critical role in web-based monitoring. Journal of Diabetes Science & Technology.3(3):461-7.

57. Lee KK, Conigrave JH, Al Ansari M, Wilson S, Perry J, Zheng C, et al. Acceptability and feasibility of a computer-based application to help Aboriginal and Torres Strait Islander Australians describe their alcohol consumption. Journal of Ethnicity in Substance Abuse. 2021;20:16-33.

58. Laurie-Ann L, Casadaya M, Shaun A, Philip S, Kelly S, Cindy J. Indigenizing forum theatre through a strength-based approach. AlterNative.

59. Koziol-McLain J, McLean C, Rohan M, Sisk R, Dobbs T, Nada-Raja S, et al. Participant Recruitment and Engagement in Automated eHealth Trial Registration: Challenges and Opportunities for Recruiting Women Who Experience Violence. J Med Internet Res.18(10):e281.

60. Kerrigan V, Herdman RM, Thomas DP, Hefler M. 'I still remember your post about buying smokes': A case study of a remote Aboriginal community-controlled health service using Facebook for tobacco control. Australian Journal of Primary Health. 2019;25(5):443-8.

61. Kennedy M, Kumar R, Ryan NM, Bennett J, La Hera Fuentes G, Gould GS. Codeveloping a multibehavioural mobile phone app to enhance social and emotional well-being and reduce health risks among Aboriginal and Torres Strait Islander women during preconception and pregnancy: a three-phased mixed-methods study. BMJ Open.11(11):e052545.

62. Kearns C, Baggott C, Harwood M, Reid A, Fingleton J, Levack W, et al. Engaging Māori with qualitative healthcare research using an animated comic. Health Promotion International. 2021;36(4):1170-7.

63. Jones EJ, Peercy M, Woods JC, Parker SP, Jackson T, Mata SA, et al. Identifying postpartum intervention approaches to reduce cardiometabolic risk among American Indian women with prior gestational diabetes, Oklahoma, 2012-2013. Preventing Chronic Disease.12:E45.

64. Johnson VB, Lorig K. The internet diabetes self-management workshop for American Indians and Alaska Natives. Health promotion practice.12:261-70.

65. Jeff C, Robynne E, Renee M, Carey N. Everyday indigenous resurgence during covid-19: A social media situation report. AlterNative.

66. Hui A, Philips-Beck W, Campbell R, Sinclair S, Kuzdak C, Courchene E, et al. Impact of remote prenatal education on program participation and breastfeeding of women in rural and remote Indigenous communities. EClinicalMedicine. 2021;35.

67. Hoffman-Goetz L, Friedman DB. A qualitative study of Canadian Aboriginal women's beliefs about "credible" cancer information on the internet. Journal of Cancer Education. 2007;22(2):124-8.

68. Hoffman-Goetz L, Donelle L. Chat room computer-mediated support on health issues for aboriginal women. Health Care Women Int.28(4):397-418.

69. Henderson JA, Chubak J, O'Connell J, Ramos MC, Jensen J, Jobe JB, et al. Design of a randomized controlled trial of a web-based intervention to reduce cardiovascular disease risk factors among remote reservation-dwelling American Indian adults with type 2 diabetes. Journal of Primary Prevention.33(4):209-22.

70. Hefler M, Kerrigan V, Thomas DP. Can Facebook help reduce smoking? A qualitative study to investigate how to use social media for tobacco control among Australian Indigenous people. Tobacco Induced Diseases. 2018;16:183-4.

71. Hefler M, Kerrigan V, Henryks J, Freeman B, Thomas DP. Social media and health information sharing among Australian Indigenous people. Health Promotion International.34(4):706-15.

72. Hefler M, Kerrigan V, Grunseit A, Freeman B, Kite J, Thomas DP. Facebook-Based Social Marketing to Reduce Smoking in Australia's First Nations Communities: An Analysis of Reach, Shares, and Likes. Journal of Medical Internet Research.22(12):e16927.

73. Hefler M, Kerrigan V, Freeman B, Boot GR, Thomas DP. Using Facebook to reduce smoking among Australian Aboriginal and Torres Strait Islander people: a participatory grounded action study. BMC Public Health.19(1):615.

74. Heaney E, Hunter L, Clulow A, Bowles D, Vardoulakis S. Efficacy of Communication Techniques and Health Outcomes of Bushfire Smoke Exposure: A Scoping Review. INTERNATIONAL JOURNAL OF ENVIRONMENTAL RESEARCH AND PUBLIC HEALTH.18(20).

75. Harris R, Van Dyke ER, Ton TGN, Nass CA, Buchwald D. Assessing Needs for Cancer Education and Support in American Indian and Alaska Native Communities in the Northwestern United States. Health Promotion Practice. 2016;17(6):891-8.

76. Hammitt LL, Vigil DE, Reid R. Tribal Sovereignty in Research and Community Engagement for a COVID-19 Vaccine Clinical Trial on the Navajo Nation: Beyond a Facebook Town Hall. American Journal of Public Health. 2021;111(8):1431-2.

77. Hamilton SJ, Mills B, Birch EM, Thompson SC. Smartphones in the secondary prevention of cardiovascular disease: A systematic review. BMC Cardiovascular Disorders. 2018;18(1).

78. Grout L, Telfer K, Wilson N, Cleghorn C, Mizdrak A. Prescribing Smartphone Apps for Physical Activity Promotion in Primary Care: Modeling Study of Health Gain and Cost Savings. Journal of Medical Internet Research.23(12).

79. Grigg M, Waa A, Bradbrook SK. Response to an indigenous smoking cessation media campaign - it's about whānau. Aust N Z J Public Health.32(6):559-64.

80. Gould GS, ra, McEwen A, Watters T, Clough AR, van der Zwan R. Should anti-tobacco media messages be culturally targeted for Indigenous populations? A systematic review and narrative synthesis. Tobacco Control: An International Journal.22(4):1-10.

81. Gould GS, LaHera Fuentes G, Bovill M, Bennett J, Hart A, Ryan N. Aligning tobacco control and smoking cessation messages with the needs of pregnant Aboriginal and Torres Strait Islander women: A rapid review. Asia-Pacific Journal of Clinical Oncology.16:22.

82. Gould GS, Holder C, Oldmeadow C, Gruppetta M. Supports Used by Aboriginal and Torres Strait Islander Women for Their Health, including Smoking Cessation, and a Baby's Health: A Cross-Sectional Survey in New South Wales, Australia. INTERNATIONAL JOURNAL OF ENVIRONMENTAL RESEARCH AND PUBLIC HEALTH.17(21).

83. Gorman JR, Clapp JD, Calac D, Kol, er C, Nyquist C, et al. Creating a culturally appropriate web-based behavioral intervention for American Indian/Alaska Native women in Southern California: the healthy women healthy Native nation study. Am Indian Alsk Native Ment Health Res. 2013;20(1):1-15.

84. Glover M, Kira A, Gentles D, Cowie N, Paton C, Moetara W. The WERO group stop smoking competition: main outcomes of a pre- and post- study. BMC Public Health.14:599.

85. Gittelsohn J, Jock B, Redmond L, Fleischhacker S, Eckmann T, Bleich SN, et al. OPREVENT2: Design of a multi-institutional intervention for obesity control and prevention for American Indian adults. BMC Public Health. 2017;17(1):1-9.

86. Gittelsohn J, Jock B, Poirier L, Wensel C, Pardilla M, Fleischhacker S, et al. Implementation of a multilevel, multicomponent intervention for obesity control in Native American communities (OPREVENT2): challenges and lessons learned. Health Educ Res.35(3):228-42.

87. Gibbons M, Fleisher L, Slamon RE, Bass S, adai V, Beck J. Exploring the potential of Web 2.0 to address health disparities. Journal of Health Communication.16:77-89.

88. George SA, Krishnamurthi R, Feigin V, Barker-Collo S. E-health for primary stroke prevention in New Zealand: A novel technological approach for holistic risk management? International Journal of Stroke.12:46.

89. Geana MV, Greiner K, Cully A, Talawyma M, Daley CM. Improving health promotion to American Indians in the Midwest United States: Preferred sources of health information and its use for the medical encounter. Journal of Community Health: The Publication for Health Promotion and Disease Prevention.37(6):1253-63.

90. Geana MV, Daley CM, Nazir N, Cully L, Etheridge J, Bledowski C, et al. Use of online health information resources by American Indians and Alaska Natives. Journal of Health Communication.17(7):820-35.

91. Friedman DB, Laditka JN, Hunter R, Ivey SL, Wu B, Laditka SB, et al. Getting the message out about cognitive health: a cross-cultural comparison of older adults' media awareness and communication needs on how to maintain a healthy brain. Gerontologist.49:S50-60.

92. Friedman DB, Hoffman-Goetz L, Friedman DB, Hoffman-Goetz L. Assessing cultural sensitivity of breast cancer information for older Aboriginal women. Journal of Cancer Education. 2007;22(2):112-8.

93. Flemington T, La Hera-Fuentes G, Bovill M, Hart A, Bennett J, Ryan NM, et al. Smoking cessation messages for pregnant aboriginal and torres strait islander women: A rapid review of peer-reviewed literature and assessment of research translation of media content. International Journal of Environmental Research and Public Health.18.

94. Firestone R, Cheng S, Dalhousie S, Hughes E, Funaki T, Henry A, et al. Exploring Pasifika wellbeing: findings from a large cluster randomised controlled trial of a mobile health intervention programme. New Zealand Medical Journal.133(1524):82-101.

95. Finlay S, Wenitong M. Aboriginal Community Controlled Health Organisations are taking a leading role in COVID‐19 health communication. Australian & New Zealand Journal of Public Health. 2020;44(4):251-2.

96. Donelle L, Hoffman-Goetz L. An exploratory study of Canadian Aboriginal online health care forums. Health Communication. 2008;23(3):270-81.

97. Dingwall KM, Sweet M, Cass A, Hughes JT, Kavanagh D, Howard K, et al. Effectiveness of Wellbeing Intervention for Chronic Kidney Disease (WICKD): results of a randomised controlled trial. BMC Nephrology.22(1):136.

98. Dingwall KM, Puszka S, Sweet M, Nagel T. "Like drawing into sand": Acceptability, feasibility, and appropriateness of a new e-mental health resource for service providers working with Aboriginal and Torres Strait Islander people. Australian Psychologist.50(1):60-9.

99. Davies J, Bukulatjpi S, Sharma S, Caldwell L, Johnston V, Davis JS. Development of a Culturally Appropriate Bilingual Electronic App About Hepatitis B for Indigenous Australians: Towards Shared Understandings. JMIR RESEARCH PROTOCOLS.4(2).

100. Cueva M, Kuhnley R, Revels LJ, Cueva K, Dignan M, Lanier AP. Bridging storytelling traditions with digital technology. International Journal of Circumpolar Health. 2013;72.

101. Clark RA, Fredericks B, Adams M, Atherton J, Howie-Esquivel J, Dracup K, et al. Addressing health literacy and cultural teaching issues in australian indigenous and non-indigenous heart failure patients using avatars: Technology development and pilot testing. Journal of Cardiac Failure.1:S115.

102. Choukou MA, Sanchez-Ramirez DC, Pol M, Uddin M, Monnin C, Syed-Abdul S. COVID-19 infodemic and digital health literacy in vulnerable populations: A scoping review. DIGITAL HEALTH.8.

103. Carlson B, Frazer R, Farrelly T. "That makes all the difference": Aboriginal and Torres Strait Islander health-seeking on social media. Health Promotion Journal of Australia.32(3):523-31.

104. Campbell AN, Turrigiano E, Moore M, Miele GM, Rieckmann T, Hu MC, et al. Acceptability of a web-based community reinforcement approach for substance use disorders with treatment-seeking American Indians/Alaska Natives. Community Mental Health Journal.51(4):393-403.

105. Buller DB, Woodall WG, Zimmerman DE, Slater MD, Heimendinger J, Waters E, et al. Randomized trial on the 5 a day, the Rio Grande way website, a web-based program to improve fruit and vegetable consumption in rural communities. Journal of Health Communication.13:230-49.

106. Buchthal OV, Doff AL, Hsu LA, Silbanuz A, Heinrich KM, Maddock JE. Avoiding a knowledge gap in a multiethnic statewide social marketing campaign: is cultural tailoring sufficient? J Health Commun.16(3):314-27.

107. Brusse C, Gardner K, McAullay D, Dowden M. Social media and mobile apps for health promotion in Australian Indigenous populations: scoping review. Journal of Medical Internet Research.16(12):e280.

108. Britt RK, Britt BC, Anderson J, Fahrenwald N, Harming S. "Sharing Hope and Healing": A Culturally Tailored Social Media Campaign to Promote Living Kidney Donation and Transplantation Among Native Americans. Health Promotion Practice.22(6):786-95.

109. Bradford D, Hansen D, Karunanithi M. Making an APPropriate Care Program for Indigenous Cardiac Disease: Customization of an Existing Cardiac Rehabilitation Program. Studies in Health Technology & Informatics. 2015;216:343-7.

110. Boyd AD, Fyfe-Johnson AL, Noonan C, Muller C, Buchwald D. Communication With American Indians and Alaska Natives About Cardiovascular Disease. Preventing Chronic Disease.17:E160.

111. Bonner A, Gillespie K, Campbell KL, Corones-Watkins K, Hayes B, Harvie B, et al. Evaluating the prevalence and opportunity for technology use in chronic kidney disease patients: a cross-sectional study. BMC NEPHROLOGY.19.

112. Berends L, Halliday R. Capacity building and social marketing promotes healthy lifestyle behaviour in an australian aboriginal community. Australian Journal of Rural Health. 2018;26(4):279-83.

113. Benatar J, Evile T, Wihongi H. Hui: a partnership in practice in familial hypercholesterolemia. The New Zealand medical journal. 2020;133(1522):63-70.

114. Bar-Zeev Y, Bovill M, Bonevski B, Gruppetta M, Oldmeadow C, Palazzi K, et al. Improving smoking cessation care in pregnancy at Aboriginal Medical Services: 'ICAN QUIT in Pregnancy' step-wedge cluster randomised study. BMJ Open.9(6):e025293.

115. Atlas A, Muru-Lanning M, Moyes S, Kerse N, Jatrana S. Cell phone and technology use by octogenarians. J Prim Health Care.12(1):35-40.

116. Alex, er J, Kwon HT, Strecher R, Bartholomew J. Multicultural media outreach: Increasing cancer information coverage in minority communities. Journal of Cancer Education. 2013;28(4):744-7.

117. Albright CL, Steffen AD, Wilkens LR, White KK, Novotny R, Nigg CR, et al. Effectiveness of a 12-month randomized clinical trial to increase physical activity in multiethnic postpartum women: results from Hawaii's Na Mikimiki Project. Preventive Medicine.69:214-23.

118. Abbass-Dick J, Brolly M, Huizinga J, Newport A, Xie F, George S, et al. Designing an ehealth breastfeeding resource with indigenous families using a participatory design. Journal of Transcultural Nursing.29(5):480-8.
